# Identification of Novel Targeting Sites of Calcineurin and CaMKII in Human Ca_V_3.2 T-Type Calcium Channel

**DOI:** 10.3390/biomedicines11112891

**Published:** 2023-10-25

**Authors:** Yu-Wang Chang, Yong-Cyuan Chen, Chien-Chang Chen

**Affiliations:** Institute of Biomedical Sciences, Academia Sinica, Taipei 11529, Taiwan; joyoyo@ibms.sinica.edu.tw

**Keywords:** phosphorylation, dephosphorylation, Ca_V_3.2 T-type calcium channel, calcineurin, CaMKII

## Abstract

The Cav3.2 T-type calcium channel is implicated in various pathological conditions, including cardiac hypertrophy, epilepsy, autism, and chronic pain. Phosphorylation of Cav3.2 by multiple kinases plays a pivotal role in regulating its calcium channel function. The calcium/calmodulin-dependent serine/threonine phosphatase, calcineurin, interacts physically with Cav3.2 and modulates its activity. However, it remains unclear whether calcineurin dephosphorylates Cav3.2, the specific spatial regions on Cav3.2 involved, and the extent of the quantitative impact. In this study, we elucidated the serine/threonine residues on Cav3.2 targeted by calcineurin using quantitative mass spectrometry. We identified six serine residues in the N-terminus, II–III loop, and C-terminus of Cav3.2 that were dephosphorylated by calcineurin. Notably, a higher level of dephosphorylation was observed in the Cav3.2 C-terminus, where calcineurin binds to this channel. Additionally, a previously known CaMKII-phosphorylated site, S1198, was found to be dephosphorylated by calcineurin. Furthermore, we also discovered that a novel CaMKII-phosphorylated site, S2137, underwent dephosphorylation by calcineurin. In CAD cells, a mouse central nervous system cell line, membrane depolarization led to an increase in the phosphorylation of endogenous Cav3.2 at S2137. Mutation of S2137 affected the calcium channel function of Cav3.2. Our findings advance the understanding of Cav3.2 regulation not only through kinase phosphorylation but also via calcineurin phosphatase dephosphorylation.

## 1. Introduction

Calcium entry through voltage-gated calcium channels depolarizes the membrane potential, facilitating the transmission of electrical signals in nerve and muscle tissues [1]. Additionally, intracellular calcium serves as a crucial secondary messenger, governing diverse cell signaling pathways and biological processes [2]. The regulation of intracellular calcium concentration involves both high-voltage-activated calcium channels (Cav1 and Cav2 subtypes) and low-voltage-activated calcium channels (Cav3 subtypes). The low-voltage-activated calcium channels, known as T-type (T for transient or tiny) channels, exhibit rapid inactivation kinetics and are capable of opening near the resting membrane potential, contributing to membrane depolarization. Vertebrates express three different T-type calcium channels: Cav3.1, Cav3.2, and Cav3.3 [3,4]. Dysfunctions in T-type calcium channels are linked to various disease conditions, including epilepsy, autism, neuromuscular disorders, and chronic pain [5]. Cav3.2 exhibits high expression levels in dorsal root ganglion sensory neurons and plays an important role in the development of chronic pain [6,7].

The pore-forming α1-subunit of T-type calcium channels consists of four homologous transmembrane domains connected by cytoplasmic N-terminus, interdomain loops, and C-terminus. These cytoplasmic regions of T-type calcium channels serve as sites of posttranslational modifications by intracellular enzymes, thereby fine-tuning the channel functions [8,9]. Deubiquitination of Cav3.2 by USP5 promotes channel stability and function, thus mediating the development of neuropathic and inflammatory pain in rodents [7]. Additionally, various kinases modulate the functions of Cav3.2 through phosphorylation. Phosphorylation of Cav3.2 at the S1107 residue in the II–III loop by PKA is required for Gβγ-mediated inhibition of Cav3.2 [10]. Phosphorylation of Cav3.2 at the S1198 residue in the II–III loop by CaMKII causes a leftward shift in the activation threshold and facilitates channel opening near the resting membrane potential [11,12]. Moreover, phosphorylation of Cav3.2 at S561 in the I–II loop and S1987 in the C-terminus by Cdk5 upregulates the channel current density [13]. Although activation of kinases, including ROCK and PKC, facilitates the Cav3.2 current, the precise phosphorylation sites at Cav3.2 remain unclear [14,15].

The activity of Cav3.2 is further influenced by specific proteins that interact with its cytoplasmic regions. Syntaxin-1A, for instance, binds to the C-terminus of Cav3.2 channels, regulating both channel function and low-threshold exocytosis [16]. Additionally, calcineurin also binds to the C-terminus of Cav3.2 channels, resulting in a reduction in the channel current density [17]. This interaction between Cav3.2 and calcineurin is dependent on calmodulin and calcium concentration. The NFAT-binding domain of calcineurin is essential for its binding to Cav3.2. Moreover, the PCISVE (2190–2195) and LTVP (2261–2264) motifs in the C-terminus of Cav3.2 are crucial for the channels’ interaction with calcineurin. The 9A-Cav3.2 mutant form, which cannot bind to calcineurin, also exhibits a higher current density [17]. 

Calcineurin is a serine/threonine phosphatase known for dephosphorylating various target proteins, including transcription factors, receptors, and channels [18]. Notably, the dephosphorylation of the transcription factor NF-AT3 by calcineurin is implicated in pathological cardiac hypertrophy [19,20]. Similarly, Cav3.2 is also involved in the development of pathological cardiac hypertrophy [21]. While it is established that calcineurin interacts with and modulates Cav3.2, the specific dephosphorylation of Cav3.2 by calcineurin has remained unclear. In this study, we aimed to identify the serine/threonine residues of Cav3.2 channels targeted by calcineurin for dephosphorylation. Additionally, we discovered that CaMKII phosphorylates one of the calcineurin-targeted residues, namely S2137. Interestingly, we observed that membrane depolarization increased the S2137 phosphorylation, as confirmed by its specific antibody. The functional implications of S2137 phosphorylation were also investigated in this study.

## 2. Materials and Methods

### 2.1. Plasmid cDNA Construction and Mutagenesis

The QuikChange site-directed mutagenesis kit from Agilent Technologies (Santa Clara, CA, USA) was employed to generate mutant plasmid constructs. Following mutagenesis, the integrity of the constructs was confirmed through sequencing. PCR was employed to amplify the C-terminus of human Cav3.2 prior to its cloning into the pGEX-4T-1 vector obtained from Thermo Fisher Scientific (Waltham, MA, USA).

### 2.2. Cell Culture and Transient Expression

Human embryonic kidney (HEK) 293 cells and mouse catecholaminergic neuronal CAD cells were cultivated in Dulbecco’s modified Eagle’s medium (DMEM), supplemented with 5% fetal bovine serum and penicillin/streptomycin, at 37 °C in a 5% CO_2_ incubator. The calcium-phosphate method was employed to transiently transfect HEK293 cells with 5 μg of plasmid DNA. Plasmids encoding Flag-tagged Cav3.2, 9A-Cav3.2, S1198A-Cav3.2, S2137A-Cav3.2, S2137D-Cav3.2, S1198A-9A-Cav3.2, S2137A-9A-Cav3.2, and S1198AS2137A-9A-Cav3.2 were individually transfected into HEK-293 cells. The GST-fusion C-terminus of Cav3.2 (GST-CII) was transfected into HEK-293 cells. Following a 48 h transfection period, the cells underwent processes including cell lysis, immunoprecipitation, and whole-cell voltage-clamp recordings. To administer cyclosporine A (CSA), the cells were subjected to a 24 h incubation with CSA following 1 day of transfection. 

### 2.3. Cell Lysis and Immunoprecipitation 

Transfected cells were lysed and homogenized using an immunoprecipitation buffer composed of the following components: (in mM) 50 Tris HCl, pH 8.0, 150 sodium chloride, 1% Triton-X100, 1 mM EDTA, protease inhibitors, and phosphatase inhibitors. The resulting lysates were incubated on ice for 30 min. Subsequently, undissolved pellets were separated by centrifugation at 13,000 rpm for 30 min at a temperature of 4 °C. For immunoprecipitation, the cell lysates were subjected to incubation with anti-FLAG antibody-conjugated beads (Sigma–Aldrich, Saint Louis, MO, USA) at 4 °C overnight, utilizing rotation. For the in vitro calcineurin reaction, the pulled-down Flag-Cav3.2 was washed successively with lysis buffer, PBS, and calcineurin reaction buffer. The Flag-Cav3.2 was eluted by the 3xFlag peptide (Sigma–Aldrich). For the GST pull-down procedure, the GST-fusion protein consisting of the C-terminus of Cav3.2 (GST-CII) was extracted using beads conjugated with glutathione (GE Healthcare, Chicago, IL, USA). Following a thorough wash, the pulled-down proteins were eluted using an excess amount of reduced glutathione [17].

### 2.4. In Vitro Calcineurin and CaMKII reactions

In the context of the in vitro calcineurin reaction, the pulled-down Flag-Cav3.2 was subjected to incubation with active calcineurin enzyme, human recombinant calmodulin, and calcineurin reaction buffer (Abcam, Cambridge, UK). For the control sample, incubation was performed without the active calcineurin enzyme. Following a 1 h incubation at 37 °C, the Flag-Cav3.2 samples were either prepared for gel-assisted digestion or immunoblotting. For the CaMKII reaction, the C-terminus of Cav3.2 (GST-CII) or Flag-Cav3.2 were incubated with CaMKII, calmodulin, and NEB buffer for protein kinases from New England Biolabs (Ipswich, MA, USA). After a 1 h incubation at 37 °C, the GST-CII samples were subjected to SDS-PAGE and stained with Coomassie blue. The resulting gel bands were excised for in-gel digestion. The CaMKII-treated Falg-Cav3.2 was prepared for immunoblotting.

### 2.5. Gel-Assisted Digestion, In-Gel Digestion, and Immobilized Metal Affinity Chromatography

To improve the digestion efficiency of membrane proteins, gel-assisted digestion was employed. [22]. To assess variations in digestion efficiency, 0.2 μg of bovine α-casein and 0.05 μg bovine β-casein were added into eluted Flag-Cav3.2 samples. Protein reduction was carried out using 5 mM TCEP, followed by room temperature alkylation with 2 mM MMTS for 30 min. For the direct incorporation of proteins into a gel within a micro tube, acrylamide/bisacrylamide solution, APS, and TEMED were added. The protein-incorporated gel was fragmented and subjected to multiple washes with 0.5 mL of 50% (*v*/*v*) ACN in TEABC. Subsequently, the dehydration of gel samples was achieved using 100% ACN and the samples were thoroughly dried using a vacuum centrifuge. Next, overnight trypsin digestion was carried out in 25 mm TEABC at 37 °C. Peptide extraction was performed by sequentially adding 0.2 mL of 25 mM TEABC, 0.2 mL of 0.1% (*v*/*v*) TFA in water, 0.2 mL of 0.1% (*v*/*v*) TFA in ACN, and 0.2 mL of 100% ACN. The collected solutions were pooled and dehydrated using a vacuum centrifuge. For the in-gel digestion of GST-CII, the gel bands were fragmented, washed with 0.5 mL of 50% (*v*/*v*) ACN in 25 mM TEABC, completely dried by 100% ACN and vacuum centrifuge, followed by trypsin digestion. The resulting peptides were then extracted. Phosphopeptide enrichment using immobilized metal affinity chromatography (IMAC) was performed following previously reported procedures [23]. The IMAC eluate and tryptic peptides were subsequently purified using a C18 Ziptip (Millipore, Bedford, MA, USA) for cleaning.

### 2.6. Mass Spectrometry (MS), Database Searching, and Phosphopeptide Quantification

The phosphopeptides enriched through IMAC and the tryptic peptides of different variants of Cav-3.2 were subjected to analysis using RP-UPLC (nanoACQUITY UPLC, Waters, Milford, MA, USA) in conjunction with Q-TOF MS (QTOF Premier, Waters), following the established procedure [23]. The MS peak lists were generated in Mascot generic format (mgf) using Mascot Distiller with default parameters. The mgf files were employed for searching against the UniProt human protein database using Mascot (Matrix Science, London, UK). The database search parameters included trypsin as the protease, allowance for up to 2 missed cleavages, and tolerances of 0.07 Da for both precursor and fragment ion measurements. Variable modifications were set to include methylthio of cysteine, oxidation of methionine, and phosphorylation of serine, threonine, and tyrosine. Proteins were considered identified if they met the significance threshold of *p* < 0.05. Peptides were considered identified if they had a peptide score of 30 or higher. The Mascot delta score was utilized for the phosphorylation site assignment [23,24]. Peptide and phosphopeptide quantification was achieved using IDEAL-Q [25]. Bovine α-casein and β-casein, added externally, served as internal references for the quantification of Cav3.2 phosphopeptides. To achieve this, each mgf file was subjected to a Mascot search against the UniProt bovine protein database. The quantities of bovine casein peptides or phosphopeptides were determined using IDEAL-Q and employed for the normalization of peptides or phosphopeptides of Cav3.2.

### 2.7. Generation of Phospho-S2137 Cav3.2 Antibody and Immunoblotting

The preparation of the rabbit polyclonal phospho-S2137 Cav3.2 antibody included the synthesis of a corresponding peptide with phosphoserine at the indicated site. This synthetic peptide was then conjugated to BSA before being used as the peptide antigen for immunizing the host rabbits. For immunoblotting, the pulled-down GST-CII or Flag-Cav3.2, the cell lysates of transfected HEK293 cells, and the cell lysates of CAD cells were separated using SDS-PAGE. Subsequently, they were transferred onto a PVDF membrane for immunostaining using specific antibodies. The following antibodies were utilized: anti-Cav3.2 (H-300, Santa Cruz Biotechnologies, Dallas, TX, USA), anti-Flag (M2-HRP, Sigma-Aldrich), anti-β actin (Proteintech, Chicago, IL, USA), and a homemade anti-phospho-S2137 Cav3.2 antibody.

### 2.8. Electrophysiological Recording

Borosilicate glass capillary tubes (Warner Instruments, Holliston, MA, USA) were utilized to shape patch pipettes, achieving a tip resistance of 2.8–3.5 MΩ using a P-97 Flaming/Brown type micropipette puller (Sutter Instrument, Novato, CA, USA). An Axon Multiclamp 700B microelectrode amplifier (Molecular Devices, San Jose, CA, USA) was employed for measuring the ionic currents. Data acquisition was performed with a sampling frequency of 50 kHz and a low-pass filter set at 2 kHz. Digidata 1440A interfaced with Clampex 10.4 (Molecular Devices, San Jose, CA, USA) controlled voltage and current commands as well as the digitization of membrane voltages and currents. Data analysis was carried out using pCLAMP 10.4 software (Molecular Devices, San Jose, CA, USA). For the measurement of Cav3.2 currents, cells were immersed in a 300 mOsm bath solution comprising 145 mM TEA-Cl, 5 mM CaCl_2_, 3 mM CsCl, 1 mM MgCl_2_, 5 mM glucose, and 10 mM HEPES, pH-adjusted to 7.4 with TEA-OH. The 310 mOsm pipette solution was composed of 130 mM CsCl, 20 mM HEPES, 10 mM EGTA, 5 mM MgCl_2_, 3 mM Mg-ATP, and 0.3 mM Tris-GTP, pH-adjusted to 7.3 with CsOH. For the measurement of voltage-dependent calcium current, the cell membrane potentials were initially held at −90 mV for 20 ms, followed by a depolarization of 10 mV for 150 ms. A 15 s waiting period was employed for channel recovery before the subsequent additional 10 mV depolarization. For the measurement of steady-state inactivation current, transfected cells were initially held at −90 mV before stepping to conditioning potentials for 1500 ms. A 10 s waiting period was employed for channel recovery before the next step. For the inhibition of calcineurin, cyclosporine A (CSA, 10 μM) was added to the bath solution.

## 3. Results

### 3.1. Identification of Amino Acid Residues on Cav3.2 Dephosphorylated by Calcineurin

Calcineurin, a calcium/calmodulin-dependent protein phosphatase, interacts with and modulates the functions of Cav3.2 T-type calcium channels [17]. To investigate whether calcineurin regulates Cav3.2 through dephosphorylation of specific serine or threonine residues, we expressed Flag-tagged human Cav3.2 in HEK293 cells, where significant phosphorylation of Cav3.2 and its phosphorylation regulation have been previously documented [12,26]. For the identification of the exact amino acid residues of Cav3.2 dephosphorylated by calcineurin, we performed mass spectrometry-based identification and label-free quantification of IMAC-enriched phosphopeptides (Supplementary Figure S1). Flag-tagged Cav3.2 was immunoprecipitated (IP) using an anti-Flag antibody and subsequently eluted with 3xFlag peptide. The pulled-down Cav3.2 channels were then reacted with or without calcineurin and digested into tryptic peptides using gel-assisted digestion [22]. The phosphopeptides were enriched through immobilized metal affinity chromatography (IMAC). The identities and quantities of peptides or phosphopeptides were revealed by LC-MS/MS analysis (Waters Q-TOF Premier) and IDEAL-Q software (V1.063) [25]. To account for quantification bias resulting from different digestion and purification efficiencies, we incorporated the standard phosphoproteins bovine α- and β-casein as spike-in controls [23]. In total, we identified 39 phosphopeptides matching Cav3.2 (Supplementary Figure S2). Among these phosphopeptides, 30 had single phosphorylation sites, 7 had double phosphorylation sites, and 2 had more than three phosphorylation sites (Table 1).

**Figure 1:**
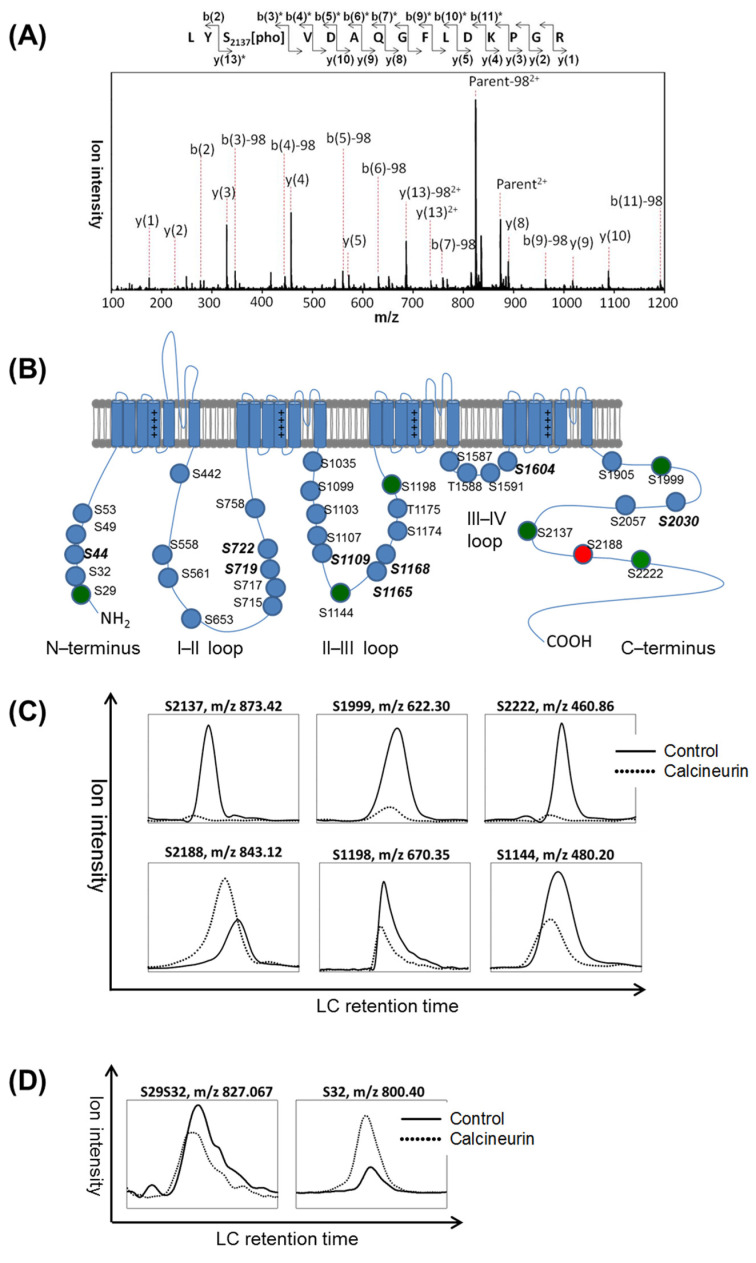
Amino acid residues on human Cav3.2 dephosphorylated by calcineurin in vitro. (**A**) MSMS spectrum of a doubly charged ion with *m*/*z* 873.41 matched a phosphopeptide belonging to human Cav3.2, spanning residues 2135 to 2149, with phosphorylation on S2137. The fragment ions experiencing a neutral loss are denoted by an asterisk (*). (**B**) Cav3.2 phosphorylation sites identified in this study. Phosphorylation of residues highlighted in green indicates a decrease, while phosphorylation of residues highlighted in red indicates an increase in response to calcineurin treatment. The phosphorylation sites emphasized in bold italic font were previously unidentified, to our knowledge. The experiment was repeated twice. The numbers of unique ions/matched spectra were 4/8, 1/3, 3/5, 4/8, 4/19, and 2/6 for S2137, S1999, S2222, S2188, S1198, and S1144, respectively. (**C**) Selective ion chromatograms (XICs) of ions with indicated *m*/*z* at specific liquid chromatography (LC) retention times corresponding to the elution times of the identified phosphopeptides in Table 1. XICs of Flag-Cav3.2 treated with or without calcineurin were compared. (**D**) A decrease in the phosphorylation of a di-phosphorylated peptide was accompanied by an increase in the signal of its singly phosphorylated counterpart. The experiment was repeated twice. The numbers of unique ions/matched spectra were 2/6 and 2/6 for S29S32 and S32, respectively.

The assignment of phosphorylation sites in a peptide was based on the Mascot delta score of each MSMS spectrum [24]. For example, the MSMS spectrum of a doubly charged phosphopeptide with a mass-to-charge ratio (*m*/*z*) of 873.41 corresponded to the amino acid residues from 2135 to 2149 of human Cav3.2. The phosphorylation site was determined as S2137, relying on the Mascot delta score difference between the first and second hits of potential candidate sequences (Figure 1A and Table 1). In this study, 36 phosphosites were assigned, with 34 phosphoserine and 2 phosphothreonine residues. These phosphosites were distributed as follows: 5 in the N-terminus, 9 in the I–II loop, 11 in the II–III loop, 4 in the III–IV loop, and 7 in the C-terminus of Cav3.2 (Figure 1B). Comparing our findings with the results of Blesneac et al. [26] and the PhosphoSitePlus database [27], we identified eight novel phosphorylation sites at S44, S719, S722, S1109, S1165, S1168, S1604, and S2030.

To identify the amino acid residues dephosphorylated by calcineurin, we compared the ion signal intensities of phosphopeptides from Cav3.2 channels treated with or without calcineurin. In Figure 1C, the selective ion chromatograms (XICs) of indicated *m*/*z* ratios matched to phosphopeptides with single phosphorylation sites were considered candidates with higher priority. We observed a decrease in ion intensities for 5 single-phosphorylated peptides upon treatment with calcineurin. Specifically, S1999, S2137, and S2222 were located in the C-terminus of Cav3.2, while S1144 and S1198 were in the II–III loop. Therefore, we suggest that calcineurin dephosphorylates Cav3.2 at S1144, S1198, S1999, S2137, and S2222.

Interestingly, the ion intensity of the S2188 single-phosphorylated peptide increased upon incubation with calcineurin. It should be noted that S2188 is located close to the calcineurin-binding motif PCISVE (amino acid 2190–2195) of Cav3.2 [17]. One possibility is that the binding of calcineurin may stabilize the phosphorylation at S2188. Another possibility is that a di-phosphorylated peptide might have undergone dephosphorylation in one residue, leading to an increased level of single-phosphorylated peptide phosphorylated in another residue. However, we did not find the corresponding di-phosphorylated peptide of S2188. Conversely, we found an S29S32 di-phosphorylated peptide whose signal intensity was decreased by calcineurin, while the corresponding S32 single-phosphorylated peptide showed an increase (Figure 1D). These results suggest calcineurin dephosphorylates Cav3.2 at N-terminus S29.

**Figure 2:**
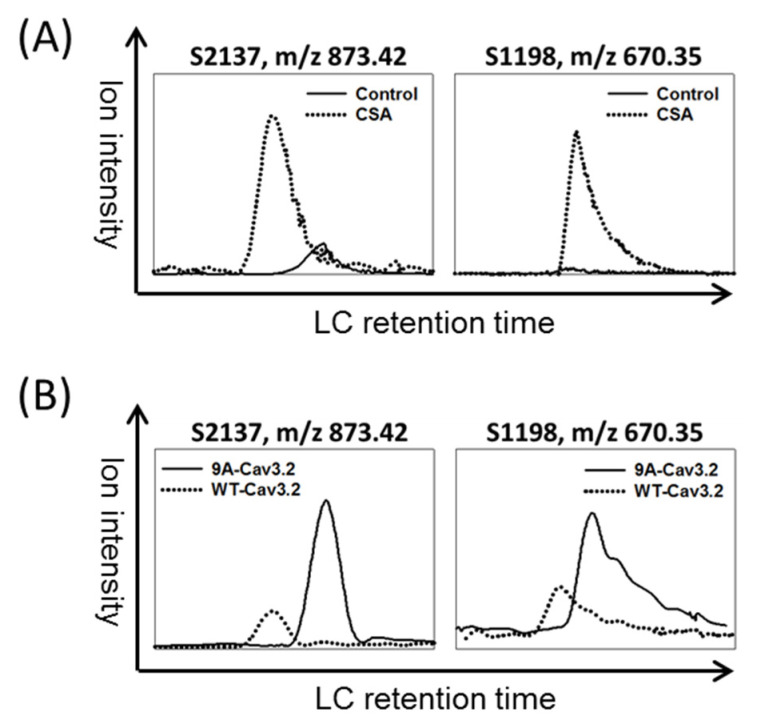
Regulation of Cav3.2 phosphorylation by calcineurin enzyme activity and binding function in HEK293 cells. (**A**) Effect of calcineurin inhibition on Cav3.2 phosphorylation. HEK293 cells were transfected with Flag-Cav3.2 for 24 h followed by a 24 h inhibition of calcineurin using cyclosporine A (CSA). The experiment was repeated twice. The numbers of unique ions/matched spectra were 3/10 and 2/4 for S2137 and S1198, respectively. (**B**) Disruption of calcineurin binding function and Cav3.2 phosphorylation. HEK293 cells were transfected with Flag-tagged wildtype Cav3.2 or a calcineurin-binding deficient mutant, 9A-Cav3.2, for 48 h. XICs of peptides with indicated phosphorylation sites were compared. The experiment was repeated twice. The numbers of unique ions/matched spectra were 3/8 and 2/4 for S2137 and S1198, respectively.

To validate the calcineurin-mediating dephosphorylation in culture cells, the Flag-Cav3.2 transfected HEK293 cells were treated with the calcineurin inhibitor cyclosporine A (CSA) for 24 h. The XICs of S1198 and S2137 phosphorylated peptides showed increased ion intensities upon CSA treatment (Figure 2A). Furthermore, the phosphorylation signals of S1198 and S2137 in a calcineurin-binding deficient mutant of Cav3.2, 9A-Cav3.2, were also increased compared to wild-type Cav3.2 (Figure 2B). These results suggest that calcineurin dephosphorylates Cav3.2 at S1198 and S2137 in HEK293 cells.

### 3.2. CaMKII Kinase Phosphorylates S2137 of Cav3.2

**Figure 3:**
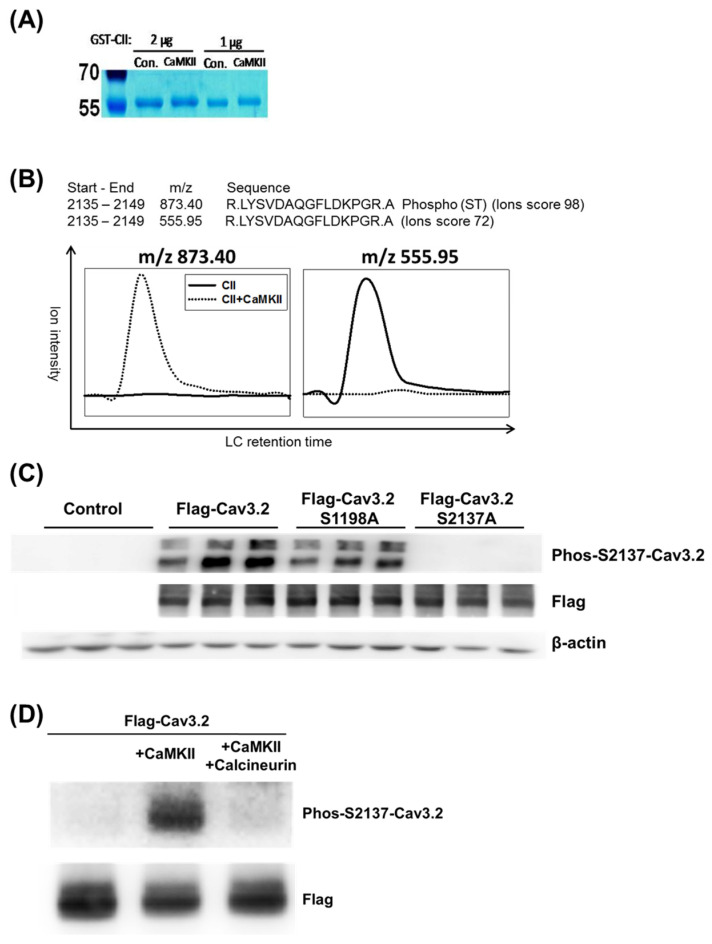
Identified S2137 as a novel CaMKII phosphorylation site on human Cav3.2. (**A**) CaMKII triggered a mobility shift in the SDS-PAGE gel for GST-CII, the GST-fusion protein derived from the C-terminus of Cav3.2. Incubation of GST-CII (1 or 2μg) with or without CaMKII was conducted. Molecular weight markers were designated in kD. *n* = 3 for control and CaMKII. (**B**) CaMKII increased the ion signal of the phospho-S2137 peptide. Tryptic peptides extracted from GST-CII gel bands were analyzed by LC-MS/MS. An ion with *m*/*z* 873.40 was identified as the phospho-S2137 peptide, while another ion with *m*/*z* 555.95 corresponded to the unmodified peptide counterpart. The experiment was repeated twice. The numbers of unique ions/matched spectra were 4/12 for S2137. (**C**) Verification of phospho-S2137 antibody specificity. The specificity of the antibody was confirmed by the signals generated from wild-type or mutant forms of Flag-tagged Cav3.2 expressed in HEK293 cells. *n* = 3 for each group. (**D**) Cav3.2 S2137 phosphorylation by CaMKII and dephosphorylation by calcineurin in the full-length Cav3.2 were confirmed using phospho-S2137 antibody. *n* = 3 for each group.

The potential kinases responsible for the identified phosphorylation sites in Cav3.2 were predicted based on kinase recognition motifs [28]. Table 2 reveals that 32 phosphorylation sites are associated with at least one potential kinase. Notably, the calcineurin-dephosphorylated sites S1198 and S2137 were both predicted to be substrates of CaMKII, PKD, or CHK1/2 kinases. Previous studies have revealed that S1198 of Cav3.2 can be phosphorylated by CaMKII [11,12]. Therefore, we sought to investigate whether S2137 is also a substrate of CaMKII. To explore this possibility, we incubated the GST-fusion C-terminus of Cav3.2 (GST-CII) with or without CaMKII. In CaMKII-treated samples, a subtle mobility shift of GST-CII bands was observed (Figure 3A). The SDS-PAGE gel bands containing GST-CII were subjected to trypsin digestion. The resulting tryptic peptides were then analyzed by LC-MS/MS and matched to the amino acid residues from 2135 to 2149 of human Cav3.2. We observed that the MSMS spectra of ions with *m*/*z* 873.40 corresponded to the S2137 phosphopeptide, while the MSMS spectra of ions with *m*/*z* 555.95 corresponded to the unphosphorylated peptide (Figure 3B). The S2137 phosphopeptide ion signal was exclusively found in the CaMKII-treated GST-CII, while the unphosphorylated peptide signal was almost absent. These results suggest that S2137 of full-length Cav3.2 can indeed be phosphorylated by CaMKII.

To further investigate the phosphorylation regulation of Cav3.2 at S2137, we generated an antibody specifically targeting the phosphorylation at this site. To confirm the antibody’s specificity for phospho-S2137, we expressed Flag-tagged wild-type, S1198A mutant, and S2137A mutant constructs of Cav3.2 into HEK293 cells. Compared with the untransfected control, the wild-type and S1198A mutant generated phospho-S2137 antibody signals. However, similar to the untransfected control, the S2137A mutant failed to generate signals using the phospho-S2137 antibody (Figure 3C). In alignment with the outcomes from our LC-MS/MS analysis, the phospho-S2137 antibody exhibited robust reactivity with the Flag-tagged full-length Cav3.2 following CaMKII treatment (Figure 3D). Furthermore, co-incubation with calcineurin led to a reduction in the phospho-S2137 antibody signal (Figure 3D). Our findings suggest that S2137 of full-length Cav3.2 undergoes phosphorylation by CaMKII and dephosphorylation by calcineurin.

**Figure 4:**
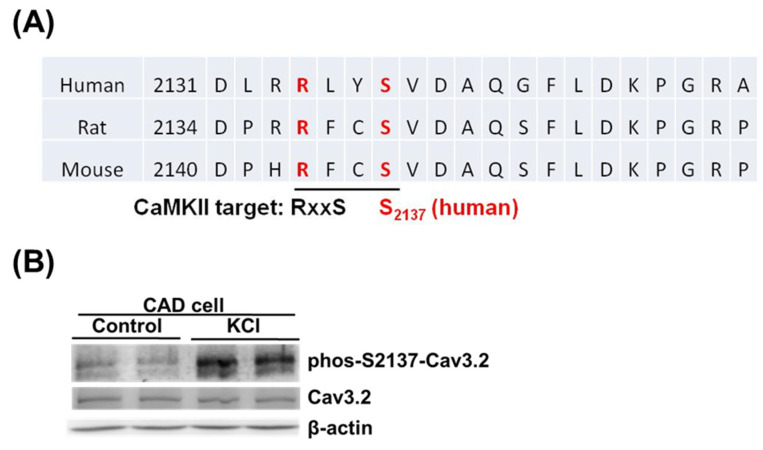
Phosphorylation of S2137 in endogenous Cav3.2 induced by membrane depolarization through KCl stimulation. (**A**) Alignment of Cav3.2 sequences from human, rat, and mouse around human Cav3.2 S2137. (**B**) KCl-induced membrane depolarization led to phosphorylation of S2137 in the native Cav3.2 of mouse CAD cells. Cells were stimulated with 50 mM KCl for 5 min. *n* = 3 for control and KCl.

To investigate the native phosphorylation of S2137 of Cav3.2, we used mouse CAD cells, which express endogenous Cav3.2 [7,29]. The homolog sequences of human, rat, and mouse were aligned in the regions around human Cav3.2 S2137. In mice and rats, the homologous sites of human Cav3.2 S2137 are also serine residues and have CaMKII recognition motifs (Figure 4A). To detect the endogenous Cav3.2 S2137 phosphorylation, we employed the phospho-S2137 antibody. We used KCl depolarization to increase the intracellular calcium concentration and CaMKII activity of mouse CAD cells [7,30]. A basal phospho-S2137 Cav3.2 signal in control CAD cells was detected by the antibody. When CAD cells were depolarized by KCl, the phosphorylation of Cav3.2 S2137 increased (Figure 4B). Our results suggest that there is endogenous phosphorylation of Cav3.2 S2137, and membrane depolarization of the neuronal cell line enhances the phosphorylation of Cav3.2 S2137.

### 3.3. Effect of S2137 Phosphorylation on the Functional Properties of Cav3.2

**Figure 5:**
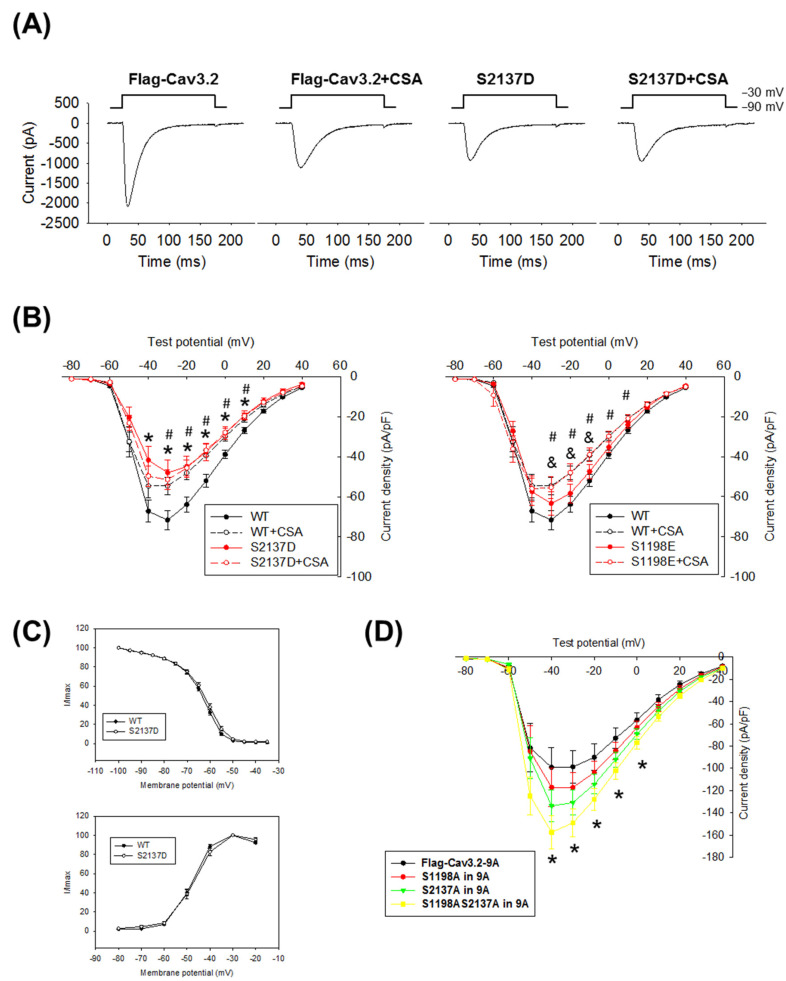
Mutation of S2137 modulated the Cav3.2 calcium channel function. To mimic phosphorylation, S2137 of Cav3.2 was mutated to aspartic acid, denoted as S2137D; and S1198 of Cav3.2 was mutated to glutamic acid, denoted as S1198E. Whole-cell voltage clamp was performed on HEK293 cells expressing either wild-type (WT) or mutant Cav3.2. The calcineurin inhibitor cyclosporine A (CSA, 10 μM) was added to the bath solution. The representative current traces elicited by the above voltage-clamp protocols are shown in (**A**). The current density–voltage plots are presented in (**B**). * *p* < 0.05 in WT vs. S2137D; # *p* < 0.05 in WT vs. WT + CSA; and & *p* < 0.05 in WT vs. S1198E + CSA. *n* = 25, 14, 13, 14, 14, 17 for WT, WT + CSA, S2137D, S2137D + CSA, S1198E, S1198E + CSA. (**C**) Steady-state inactivation curves and voltage-dependent curves of wild-type and S2137D Cav3.2. (**D**) Phosphorylation of S1198 and S2137 directly impacted the Cav3.2 calcium current. Whole-cell voltage clamp was performed on HEK293 cells expressing 9A-mutant (*n* = 14), S1198A in 9A (*n* = 12), S2137A in 9A (*n* = 18), or S1198AS2137A in 9A (*n* = 13). * *p* < 0.05 compared to Flag-Cav3.2-9A.

To investigate the impact of Cav3.2 S2137 phosphorylation on the calcium current properties of Cav3.2, we expressed the S2137D phosphorylation mimic mutant in HEK293 cells. Additionally, we validated the functional regulation of Cav3.2 through calcineurin-mediated dephosphorylation using the specific phosphatase inhibitor CSA. The voltage-gated channel properties of wild-type and phosphorylation-mimicking mutants of Cav3.2 were compared using a whole-cell voltage clamp. Transfected cells were held at −90 mV and then subjected to test potentials. The representative current traces exhibited typical T-type calcium channel behavior (Figure 5A). The current densities of the Cav3.2 S2137D mutant were significantly smaller than those of wild-type Cav3.2 (Figure 5B). Inhibition of calcineurin-mediated dephosphorylation by CSA increased phosphorylation on S2137 of Cav3.2 (Figure 2A) and led to a reduction in calcium current densities of wild-type Cav3.2 (Figure 5B). CSA failed to affect the current densities of S2137D-phosphorylation-mimicking Cav3.2, and this suggests that phosphorylation on S2137 of Cav3.2 is sufficient to inhibit the Cav3.2 calcium channel function. Although CSA also increased phosphorylation on S1198 of Cav3.2 (Figure 2A), the S1198E-phosphorylation-mimicking Cav3.2 itself could not significantly reduce the current densities of Cav3.2 unless further inhibiting the calcineurin-mediated dephosphorylation with CSA (Figure 5B). The above findings indicate that in the regulation of Cav3.2 current density by calcineurin, S2137 holds greater significance compared to S1198. Additionally, the voltage-dependent activation and steady-state inactivation curves indicated similar calcium channel gating properties between wild-type and S2137D Cav3.2 (Figure 5C).

Calcineurin binds to Cav3.2 [17] and also dephosphorylates Cav3.2. In Figure 2B, the phosphorylation levels on S1198 and S2137 of Cav3.2 were increased in the calcineurin-binding-deficient 9A mutant of Cav3.2. To distinguish between the effects of calcineurin binding and dephosphorylation, we introduced the phospho-deficient S1198A and S2137A mutants into the calcineurin-binding-deficient 9A mutant of Cav3.2. In the 9A mutant, single mutations in S1198A or S2137A led to increased current densities of Cav3.2, but these changes did not reach statistical significance. However, when both sites were mutated to S1198AS2137A, the current density was significantly increased (Figure 5D). Our results suggest that phosphorylation of S2137 of Cav3.2 inhibits the current densities of Cav3.2 calcium channels, and dephosphorylation of Cav3.2 by calcineurin enhances the current densities of Cav3.2 calcium channels.

## 4. Discussion

Previously, the phosphorylation of Cav3.2 by various kinases was elucidated [9]. In this study, we identified dephosphorylation sites on Cav3.2 by calcineurin, both in vitro and in vivo. We discovered that calcineurin dephosphorylates the previously identified CaMKII target site, S1198, on Cav3.2. Additionally, we revealed that a novel CaMKII target site, S2137, on Cav3.2 is also subjected to dephosphorylation by calcineurin. To specifically recognize phospho-S2137 Cav3.2, we generated an antibody, and with its application, we confirmed that membrane depolarization increases the phosphorylation of Cav3.2 at S2137. Lastly, we observed that S2137 phosphorylation modulates the calcium channel function of Cav3.2.

In this study, our findings indicate that the residues in the C-terminus of Cav3.2 undergo more significant dephosphorylation by calcineurin when compared to the residues in the II–III loop. The docking of calcineurin to its substrates is a crucial step in the dephosphorylation of various calcineurin targets [31]. Moreover, the specificity of calcineurin-mediated dephosphorylation relies more on the structural characteristics of substrates rather than a specific consensus sequence [32]. Notably, our findings demonstrate that the sites on Cav3.2 dephosphorylated by calcineurin lack a distinct sequence pattern. Since the substrate-binding site is located within the catalytic domain of calcineurin, a higher degree of dephosphorylation is expected in the C-terminus of Cav3.2, as observed in our study. Interestingly, the phosphorylation level of Cav3.2 at S2188, which is situated close to the PCISVE (2190–2195) calcineurin binding motif of Cav3.2, was found to be augmented by the addition of calcineurin. This observation raises the possibility that the protein/protein binding region might create an environment conducive to stabilizing the phosphorylated motifs.

Previously, CaMKII had been identified as the enzyme phosphorylating S1198 of Cav3.2, a process that facilitates the opening of channels near the membrane potential [11,12]. However, in the current study, we uncovered that S1198 of Cav3.2 can also be targeted for dephosphorylation by calcineurin. Moreover, our investigation revealed a previously unknown phosphorylation target of CaMKII, S2137 of Cav3.2, which, interestingly, is also subject to dephosphorylation by calcineurin. Notably, when analyzing CAD cells that naturally express Cav3.2, we detected an increased signal of phospho-S2137 Cav3.2 antibody after membrane depolarization caused by KCl. Given that membrane depolarization prompts the opening of Cav3.2, our findings suggest that calcium influx through these channels might stimulate the phosphorylation of Cav3.2 by CaMKII, rather than inducing dephosphorylation by calcineurin. In earlier investigations, we observed a peak binding of calcineurin with Cav3.2 at a calcium concentration of 30 μM, along with 20% binding at a calcium concentration of 1 μM [17]. Given the usual cytoplasmic calcium concentration span from 0.1 μM in resting cells to 1 μM in depolarized cells [33], it becomes plausible that the activation of calcineurin could take place in scenarios where there is an excessive influx of calcium through Cav3.2. Moreover, given that CaMKII binds to the II–III loop where S1198 is located, and calcineurin binds to the C-terminus where S2137 is situated, it is plausible that CaMKII would have a preference for phosphorylating S1198 over S2137, while calcineurin could have a predilection for dephosphorylating S2137 rather than S1198. These spatial arrangements of upstream regulators and downstream target sites contribute to the nuanced fine-tuning of the Cav3.2 channel function. We are of the opinion that the dephosphorylation of Cav3.2, in conjunction with its phosphorylation by CaMKII or potentially other kinases, plays a crucial role in maintaining the functional homeostasis of Cav3.2. This is particularly significant considering its involvement in conditions such as chronic pain, autism, epilepsy, and primary aldosteronism [4,5].

Owing to advancements in mass spectrometry technology, the identification of phosphorylation sites in proteins of interest is now a commonly conducted practice [34]. Our study underscores the potency of mass spectrometry technology in uncovering new phosphorylation sites, even within proteins that have been extensively studied before. The ability to detect previously undiscovered phosphopeptides could arise from variations in enzyme digestion techniques for membrane proteins, phosphopeptide enrichment strategies, and mass spectrometry analysis protocols. Consequently, it remains a challenge to exhaustively identify all phosphorylation sites of a purified protein using a singular analytical approach. Enhancing identification outcomes can be achieved through a combination of diverse methods involving protein digestion, phosphopeptide enrichment, and mass spectrometry analysis. Previously, Blesneac et al. identified 34 distinct phosphorylation sites from rat brains and 43 phosphorylation sites from human Cav3.2 overexpressed in HEK293T cells using mass spectrometry technology [26]. In this study, we identified 36 phosphorylation sites in human Cav3.2, and among them, 8 phosphorylation sites are novel, to our knowledge. The potential implications of these Cav3.2 phosphorylation sites can be speculated by comparing them with variant sequences of Cav3.2 from humans with phenotypes in the ClinVar database [35]. In addition to identification, our study expands the scope by incorporating phosphopeptide quantification, allowing us to uncover novel target sites of calcineurin and CaMKII. Furthermore, this phosphopeptide quantification strategy revealed varying fold changes among the target sites, indicating differences in the prioritization of phosphorylation or dephosphorylation among these targets. Regarding the importance of functional implications, it is worth noting that a search in the ClinVar database unveiled mutations at calcineurin-targeted sites, including S29F, S1198D, S1999F, S2188N, and S2222Y. These mutations have been linked to conditions such as type IV familial hyperaldosteronism and idiopathic generalized epilepsy in the ClinVar database.

Certain clinical agents are categorized as T-type calcium channel blockers and are used for treating epilepsy and hypertension [36]. Moreover, T-type calcium channel blockers also exhibit promising potential for pain management [37]. Given that there are three subtypes of T-type calcium channels in humans, namely Cav3.1, Cav3.2, and Cav3.3, the development of subtype-specific inhibitors for these channels is considered essential for both therapeutic and research purposes [38]. Research has shown that intrathecal administration of the deubiquitination target peptide of Cav3.2 to mice resulted in an analgesic effect in the context of neuropathic and inflammatory pain [7]. Cell-permeable phosphopeptides have been employed to either inhibit or stimulate intracellular signaling pathways [39]. We are confident that identifying Cav3.2 phosphopeptides regulated by kinases or phosphatases will advance our comprehension of channel regulation and consequently contribute to the development of treatment strategies.

## 5. Conclusions

The current study has unveiled the sites on Cav3.2 channels that undergo dephosphorylation by calcineurin. Among these calcineurin-dephosphorylated residues, S1198, situated in the II–III loop of Cav3.2, had been previously identified as a target site for CaMKII phosphorylation. Additionally, we have identified a novel site, S2137, located in the C-terminus of Cav3.2, which is both phosphorylated by CaMKII and dephosphorylated by calcineurin. Notably, membrane depolarization in mouse CAD cells led to the phosphorylation of S2137, a phenomenon confirmed by the specific antibody designed for this purpose. Furthermore, our study delved into the functional implications associated with S2137 phosphorylation.

## Figures and Tables

**Table 1:** Human Cav3.2 phosphorylation sites identified from indicated mass spectra.

Spectra_No. ^a^	Exp_mz ^b^	Phosphopeptide_Matched ^c^	Start ^d^	End ^d^	Site ^e^	Score ^f^	Delta_Score ^g^
1	800.40	VPLGAPPPGPAALVGASPE*pS*PGAPGR	13	38	S32	70	20
2	827.07	VPLGAPPPGPAALVGA*pS*PE*pS*PGAPGR	13	38	S29, S32	48	39
3	1069.48	EAERG*pS*ELGVSPSESPAAER	39	58	S44	133	50
4	826.87	GSELGV*pS*PSESPAAER	43	58	S49	86	19
5	866.85	GSELGV*pS*PSE*pS*PAAER	43	58	S49, S53	75	11
6	766.64	EAERG*pS*ELGV*pS*PSE*pS*PAAER	39	58	S44, S49, S53	36	6
7	1327.08	HL*pS*NDSTLASFSEPGSCYEELLK	440	462	S442	84	7
8	585.77	AGAPP*pS*PPSPGR	553	564	S558	37	16
9	585.77	AGAPPSPP*pS*PGR	553	564	S561	31	21
10	625.75	AGAPP*pS*PP*pS*PGR	553	564	S558, S561	35	26
11	824.38	WAGGPPGTGGHGPLSLN*pS*PDPYEK	636	659	S653	40	8
12	1043.92	ALEDPEGEL*pS*GSESGDSDGR	706	725	S715	128	29
13	1083.90	ALEDPEGEL*pS*G*pS*ESGDSDGR	706	725	S715, S717	93	25
14	1177.44	ALEDPEGELpSGpSEpSGDpSDGRGVYEFTQDVR	706	735	S715, S717, S719, S722	54	10
15	709.30	ATDTPGPGPG*pS*PQR	748	761	S758	47	24
16	1123.46	SDTDEDKT*pS*VHFEEDFHK	1027	1044	S1035	69	7
17	820.38	SSPFLDAAP*pS*LPDSR	1090	1104	S1099	74	14
18	860.37	SSPFLDAAP*pS*LPD*pS*R	1090	1104	S1099, S1103	36	13
19	898.43	SSPFLDAAPSLPD*pS*RR	1090	1105	S1103	41	21
20	730.35	RG*pS*SSSGDPPLGDQKPPASLR	1105	1125	S1107	77	6
21	1135.00	RG*pS*S*pS*SGDPPLGDQKPPASLR	1105	1125	S1107, S1109	43	6
22	558.26	S*pS*WSSLGR	1143	1150	S1144	43	10
23	711.29	E*pS*LLSGEGKGSTDDEAEDGR	1164	1183	S1165	49	7
24	500.23	ESLL*pS*GEGK	1164	1172	S1168	55	30
25	711.29	ESLLSGEGKG*pS*TDDEAEDGR	1164	1183	S1174	75	6
26	616.21	GS*pT*DDEAEDGR	1173	1183	T1175	36	9
27	1106.43	ESLLSGEGKG*pSpT*DDEAEDGR	1164	1183	S1174, T1175	113	35
28	670.35	AE*pS*LDPRPLRPAALPPTK	1196	1213	S1198	30	23
29	504.58	RR*pS*TFPSPEAQR	1585	1596	S1587	47	12
30	531.23	RR*pSpT*FPSPEAQR	1585	1596	S1587, T1588	32	8
31	600.26	STFP*pS*PEAQR	1587	1596	S1591	34	18
32	542.25	RPYYADY*pS*PTRR	1597	1608	S1604	46	11
33	892.92	VDADRPPLPQE*pS*PGAR	1894	1909	S1905	50	31
34	622.30	SGEPLHAL*pS*PR	1991	2001	S1999	62	38
35	910.42	ID*pS*PRDTLDPAEPGEK	2028	2043	S2030	55	22
36	619.65	TPVRPVTQGGSLQ*pS*PPR	2044	2060	S2057	91	36
37	873.42	LY*pS*VDAQGFLDKPGR	2135	2149	S2137	92	33
38	839.13	KM*pS*PPCISVEPPAEDEGSARPSAAEGGSTTLR	2186	2217	S2188	42	12
39	460.86	RTP*pS*CEATPHR	2219	2229	S2222	46	14

^a^ The MSMS spectrum is designated as Supplementary Figure S2. ^b^ Exp_mz: observed *m*/*z* ratio. ^c^ Phosphorylation sites are indicated with “*p*” before the abbreviation of serine (S) or threonine (T). ^d^ Amino acid positions denoting the beginning and end of phosphopeptides in human Cav3.2. ^e^ Amino acid positions indicating the sites of phosphorylation. ^f^ The Mascot score of the identified phosphopeptide. ^g^ The Mascot delta score of the specified MSMS spectrum.

**Table 2:** Potential kinases targeting human Cav3.2 phosphorylation sites.

Site	Domain	Predicted Kinase Motif ^a^
S32	N-terminal	CK1 (S-X-X-S/T) ERK (P-X-S/T-P, P-E-S/T-P)
S44	N-terminal	PKA (R-X-S/T)
S49	N-terminal	CK2 (S/T-X-X-E) GSK3 (S-X-X-X-S) NEK6 (L-X-X-S/T)
S53	N-terminal	CK1 (S/T-X-X-X-S)
S442	I–II loop	CaMKII (R-X-X-S/T) AKT (R-X-R-X-X-S/T)
S558	I–II loop	ERK (P-X-S/T-P)
S561	I–II loop	CK1 (S-X-X-S/T) CDK2 (S/T-P-X-K/R) ERK (P-X-S/T-P) CDK1 (S/T-P-X-K/R)
S653	I–II loop	CK1 (S-X-X-S/T)
S715	I–II loop	CK2 (S/T-X-X-E) GSK3 (S-X-X-X-S)
S717	I–II loop	NEK6 (L-X-X-S/T)
S719	I–II loop	CK1 (S/T-X-X-X-S)
S722	I–II loop	CK1 (S-X-X-S/T)
S758	I–II loop	CDK2 (S/T-P-X-K/R) ERK (P-X-S/T-P) CDK1 (S/T-P-X-K/R)
S1035	II–III loop	Aurora (R/K-X-S/T-I/L/V)
S1099	II–III loop	GSK3 (S-X-X-X-S)
S1103	II–III loop	CK1 (S/T-X-X-X-S) GSK3 (S-X-X-X-S) NEK6 (L-X-X-S/T)
S1107	II–III loop	PKA (R-X-S/T, R-R/K-X-S/T) CK1 (S/T-X-X-X-S) CaMKII (R-X-X-S/T) AKT (R-R/S/T-X-S/T-X-S/T)
S1109	II–III loop	CK1 (S-X-X-S/T)
S1144	II–III loop	PKA (R-X-S/T, R-R/K-X-S/T) CK1 (S/T-X-X-X-S) CaMKII (R-X-X-S/T) AKT (R-R/S/T-X-S/T-X-S/T)
S1165	II–III loop	PKA (R-X-S/T) Aurora (R/K-X-S/T-I/L/V)
S1168	II–III loop	CK1 (S-X-X-S/T)
T1175	II–III loop	CK2 (S/T-X-X-E)
S1198	II–III loop	CaMKII (R-X-X-S/T) PKD (L/V/I-X-R/K-X-X-S/T) CHK1/2 (L-X-R-X-X-S/T) CHK1 (M/I/L/V-X-R/K-X-X-S/T)
S1587	III–IV loop	PKA (R-X-S/T, R-R/K-X-S/T) GSK3 (S-X-X-X-S) CaMKII (R-X-X-S/T)
T1588	III–IV loop	PKA (R-X-S/T, R-R/K-X-S/T) CaMKII (R-X-X-S/T) AKT (R-X-R-X-X-S/T)
S1591	III–IV loop	CK1 (S/T-X-X-X-S)
S1604	III–IV loop	CDK2 (S/T-P-X-K/R) CDK1 (S/T-P-X-K/R)
S1999	C-terminal	CDK1 (S/T-P-K/R)
S2030	C-terminal	CDK1 (S/T-P-K/R)
S2057	C-terminal	CK1 (S-X-X-S/T) GSK3 (S-X-X-X-S) CDK2 (S/T-P-X-K/R) CDK1 (S/T-P-X-K/R)
S2137	C-terminal	CaMKII (R-X-X-S/T, R-X-X-S/T-V) PKD (L/V/I-X-R/K-X-X-S/T) CHK1/2 (L-X-R-X-X-S/T) CHK1 (M/I/L/V-X-R/K-X-X-S/T)
S2222	C-terminal	CaMKII (R-X-X-S/T) AKT (R-X-R-X-X-S/T)

^a^ Prediction was conducted using the entire amino acid sequence of human Cav3.2 through Phosida.

## Data Availability

Not applicable.

## Supplementary Materials

The following supporting information can be downloaded at: https://www.mdpi.com/article/10.3390/biomedicines11112891/s1, Figure S1: Approach for identifying calcineurin-dephosphorylated residues on Cav3.2 T-type calcium channel; Figure S2: Mascot search results of MSMS spectra.
