# Supplementary material for: Identification of Novel Targeting Sites of Calcineurin and CaMKII in Human CaV3.2 T-Type Calcium Channel
Source: Biomedicines. 2023 Oct 25;11(11):2891. doi: 10.3390/biomedicines11112891 (PMC10669385; doi:10.3390/biomedicines11112891)

Figure S1. Approach for identifying calcineurin-dephosphrylated residues on Cav3.2 T-type calcium channel.

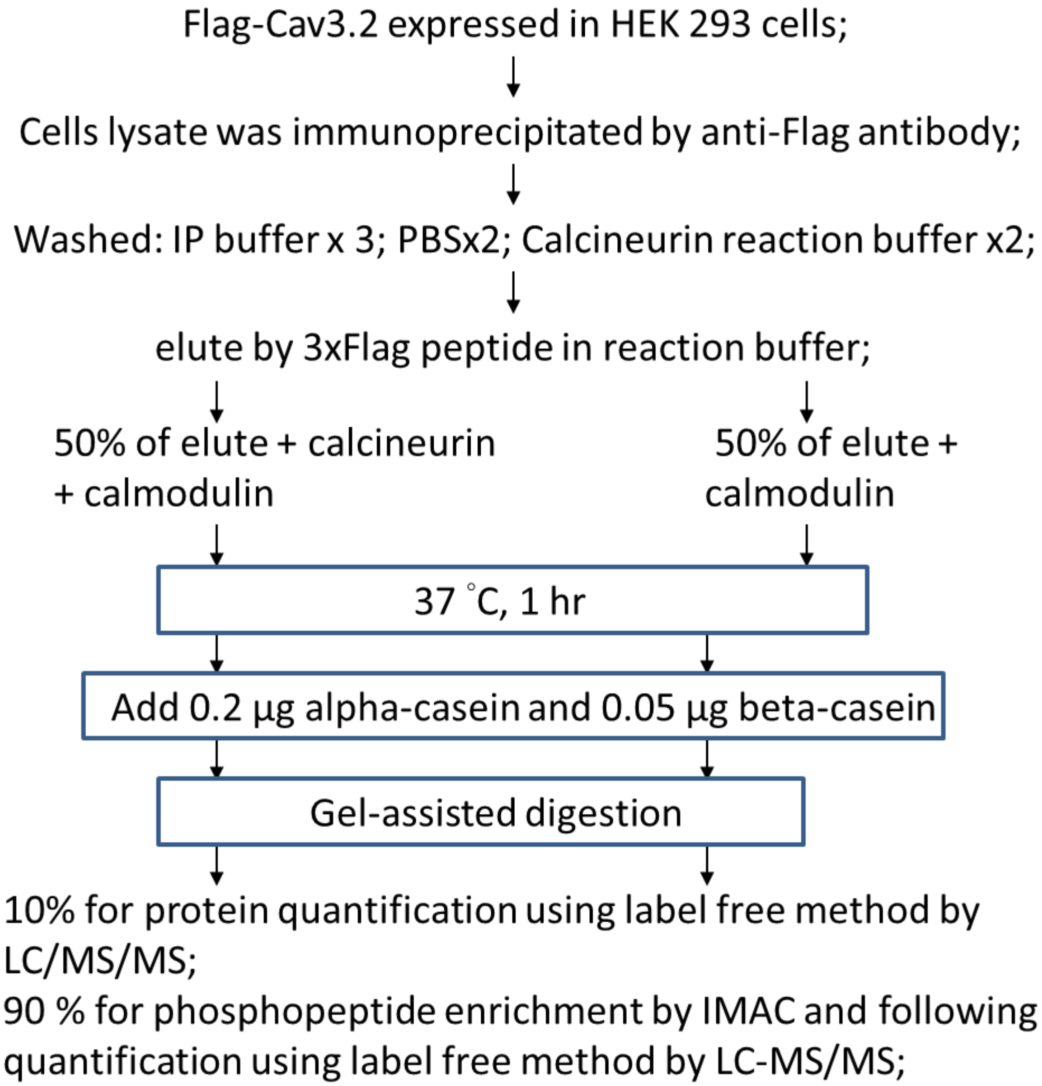

Figure S2. Mascot search results of MSMS spectra

1_MS/MS Fragmentation of **VPLGAPPPGPAALVGASPESPGAPGR**

Found in **O95180**, Voltage-dependent T-type calcium channel subunit alpha-1H OS=Homo sapiens GN=CACNA1H PE=1 SV=4

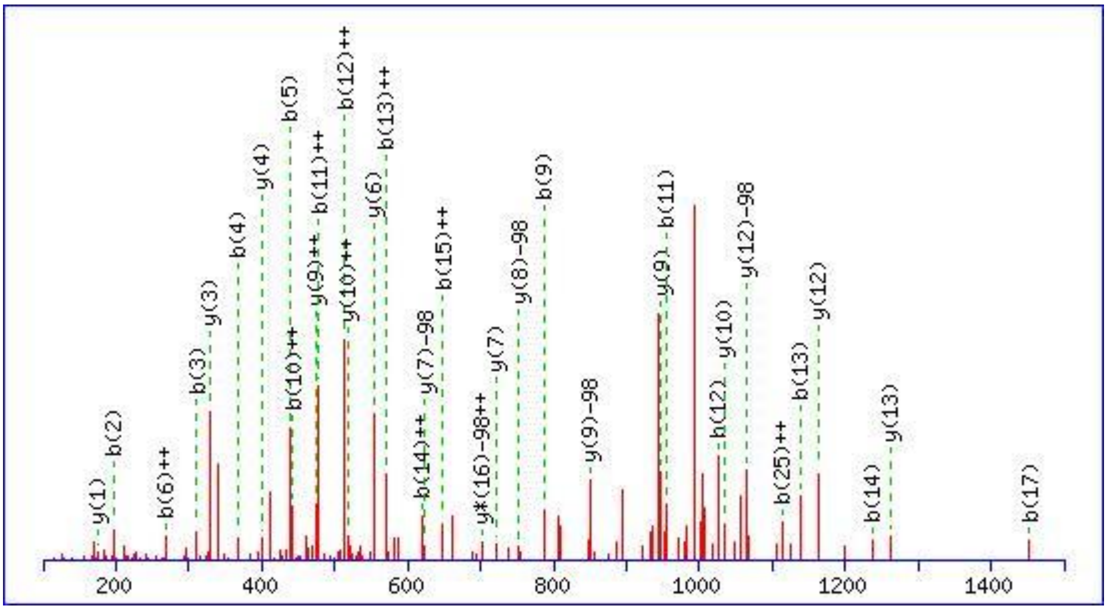

Monoisotopic mass of neutral peptide Mr(calc): 2398.2046

Variable modifications:

S20 : Phospho (ST), with neutral losses 0.0000(shown in table), 97.9769

Ions Score: 70 Expect: 0.00026

Matches : 34/344 fragment ions using 57 most intense peaks (help)

#	b	b^{++}	b^{0}	b^{0++}	Seq.	y	y^{++}	y*	y^{*++}	y^{0}	y^{0++}	#
1	100.0757	50.5415			V							26
2	197.1285	99.0679			P	2300.1435	1150.5754	2283.1169	1142.0621	2282.1329	1141.5701	25
3	310.2125	155.6099			L	2203.0907	1102.0490	2186.0642	1093.5357	2185.0801	1093.0437	24
4	367.2340	184.1206			G	2090.0066	1045.5070	2072.9801	1036.9937	2071.9961	1036.5017	23
5	438.2711	219.6392			A	2032.9852	1016.9962	2015.9586	1008.4830	2014.9746	1007.9909	22
6	535.3239	268.1656			P	1961.9481	981.4777	1944.9215	972.9644	1943.9375	972.4724	21
7	632.3766	316.6920			P	1864.8953	932.9513	1847.8688	924.4380	1846.8847	923.9460	20
8	729.4294	365.2183			P	1767.8425	884.4249	1750.8160	875.9116	1749.8320	875.4196	19
9	786.4509	393.7291			G	1670.7898	835.8985	1653.7632	827.3853	1652.7792	826.8932	18
10	883.5036	442.2554			P	1613.7683	807.3878	1596.7418	798.8745	1595.7577	798.3825	17
11	954.5407	477.7740			A	1516.7156	758.8614	1499.6890	750.3481	1498.7050	749.8561	16
12	1025.5778	513.2926			A	1445.6784	723.3429	1428.6519	714.8296	1427.6679	714.3376	15
13	1138.6619	569.8346			L	1374.6413	687.8243	1357.6148	679.3110	1356.6308	678.8190	14
14	1237.7303	619.3688			V	1261.5573	631.2823	1244.5307	622.7690	1243.5467	622.2770	13
15	1294.7518	647.8795			G	1162.4888	581.7481	1145.4623	573.2348	1144.4783	572.7428	12
16	1365.7889	683.3981			A	1105.4674	553.2373	1088.4408	544.7241	1087.4568	544.2320	11
17	1452.8209	726.9141	1434.8104	717.9088	S	1034.4303	517.7188	1017.4037	509.2055	1016.4197	508.7135	10

**18**	1549.8737	775.4405	1531.8631	766.4352	**P**	***947.3982***	474.2028	930.3717	465.6895	929.3877	465.1975	**9**
**19**	1678.9163	839.9618	1660.9057	830.9565	**E**	850.3455	425.6764	833.3189	417.1631	832.3349	416.6711	**8**
**20**	1845.9146	923.4610	1827.9041	914.4557	**S**	***721.3029***	361.1551	704.2763	352.6418	703.2923	352.1498	**7**
**21**	1942.9674	971.9873	1924.9568	962.9821	**P**	***554.3045***	277.6559	537.2780	269.1426			**6**
**22**	1999.9889	1000.4981	1981.9783	991.4928	**G**	457.2518	229.1295	440.2252	220.6162			**5**
**23**	2071.0260	1036.0166	2053.0154	1027.0113	**A**	***400.2303***	200.6188	383.2037	192.1055			**4**
**24**	2168.0788	1084.5430	2150.0682	1075.5377	**P**	***329.1932***	165.1002	312.1666	156.5870			**3**
**25**	2225.1002	***1113.0537***	2207.0896	1104.0485	**G**	232.1404	116.5738	215.1139	108.0606			**2**
**26**					**R**	***175.1190***	88.0631	158.0924	79.5498			**1**

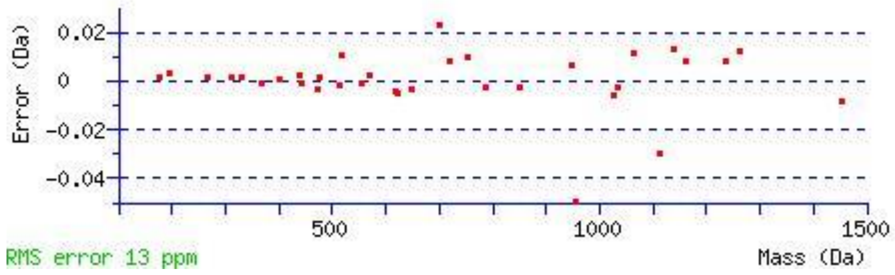

2_MS/MS Fragmentation of **VPLGAPPPGPAALVGASPESPGAPGR**

Found in **O95180**, Voltage-dependent T-type calcium channel subunit alpha-1H OS=Homo sapiens GN=CACNA1H PE=1 SV=4

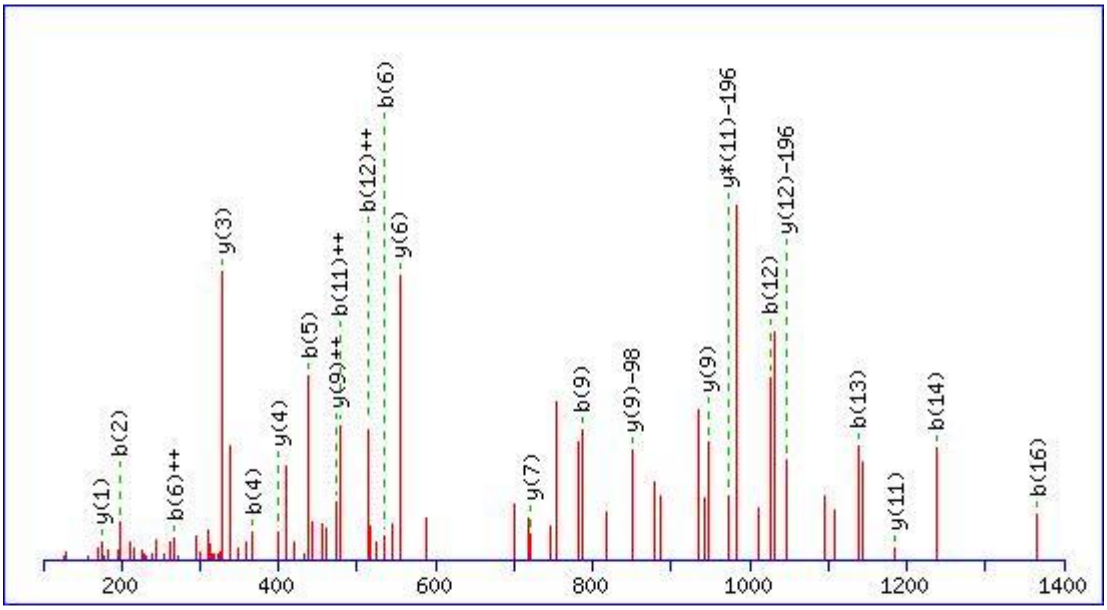

Monoisotopic mass of neutral peptide Mr(calc): 2478.1709

Variable modifications:

S17 : Phospho (ST), with neutral losses 0.0000(shown in table), 97.9769

S20 : Phospho (ST), with neutral losses 0.0000(shown in table), 97.9769

Ions Score: 48 Expect: 0.044

Matches : 23/356 fragment ions using 50 most intense peaks (help)

#	b	b^{++}	b^{0}	b^{0++}	Seq.	y	y^{++}	y*	y^{*++}	y^{0}	y^{0++}	#
1	100.0757	50.5415			V							26
2	197.1285	99.0679			P	2380.1098	1190.5585	2363.0833	1182.0453	2362.0992	1181.5533	25
3	310.2125	155.6099			L	2283.0570	1142.0322	2266.0305	1133.5189	2265.0465	1133.0269	24
4	367.2340	184.1206			G	2169.9730	1085.4901	2152.9464	1076.9769	2151.9624	1076.4848	23
5	438.2711	219.6392			A	2112.9515	1056.9794	2095.9250	1048.4661	2094.9409	1047.9741	22
6	535.3239	268.1656			P	2041.9144	1021.4608	2024.8879	1012.9476	2023.9038	1012.4556	21
7	632.3766	316.6920			P	1944.8616	972.9345	1927.8351	964.4212	1926.8511	963.9292	20
8	729.4294	365.2183			P	1847.8089	924.4081	1830.7823	915.8948	1829.7983	915.4028	19
9	786.4509	393.7291			G	1750.7561	875.8817	1733.7296	867.3684	1732.7455	866.8764	18
10	883.5036	442.2554			P	1693.7346	847.3710	1676.7081	838.8577	1675.7241	838.3657	17
11	954.5407	477.7740			A	1596.6819	798.8446	1579.6553	790.3313	1578.6713	789.8393	16
12	1025.5778	513.2926			A	1525.6448	763.3260	1508.6182	754.8127	1507.6342	754.3207	15
13	1138.6619	569.8346			L	1454.6077	727.8075	1437.5811	719.2942	1436.5971	718.8022	14
14	1237.7303	619.3688			V	1341.5236	671.2654	1324.4970	662.7522	1323.5130	662.2602	13
15	1294.7518	647.8795			G	1242.4552	621.7312	1225.4286	613.2180	1224.4446	612.7259	12
16	1365.7889	683.3981			A	1185.4337	593.2205	1168.4072	584.7072	1167.4231	584.2152	11
17	1532.7873	766.8973	1514.7767	757.8920	S	1114.3966	557.7019	1097.3700	549.1887	1096.3860	548.6967	10

**18**	1629.8400	815.4236	1611.8295	806.4184	**P**	***947.3982***	474.2028	930.3717	465.6895	929.3877	465.1975	**9**
**19**	1758.8826	879.9449	1740.8721	870.9397	**E**	850.3455	425.6764	833.3189	417.1631	832.3349	416.6711	**8**
**20**	1925.8810	963.4441	1907.8704	954.4388	**S**	***721.3029***	361.1551	704.2763	352.6418	703.2923	352.1498	**7**
**21**	2022.9337	1011.9705	2004.9232	1002.9652	**P**	***554.3045***	277.6559	537.2780	269.1426			**6**
**22**	2079.9552	1040.4812	2061.9446	1031.4760	**G**	457.2518	229.1295	440.2252	220.6162			**5**
**23**	2150.9923	1075.9998	2132.9818	1066.9945	**A**	***400.2303***	200.6188	383.2037	192.1055			**4**
**24**	2248.0451	1124.5262	2230.0345	1115.5209	**P**	***329.1932***	165.1002	312.1666	156.5870			**3**
**25**	2305.0665	1153.0369	2287.0560	1144.0316	**G**	232.1404	116.5738	215.1139	108.0606			**2**
**26**					**R**	***175.1190***	88.0631	158.0924	79.5498			**1**

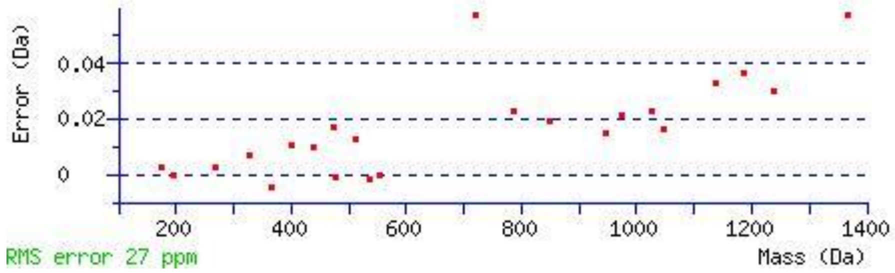

3_MS/MS Fragmentation of **EAERGSELGVSPSESPAAER**

Found in **O95180**, Voltage-dependent T-type calcium channel subunit alpha-1H OS=Homo sapiens GN=CACNA1H PE=1 SV=4

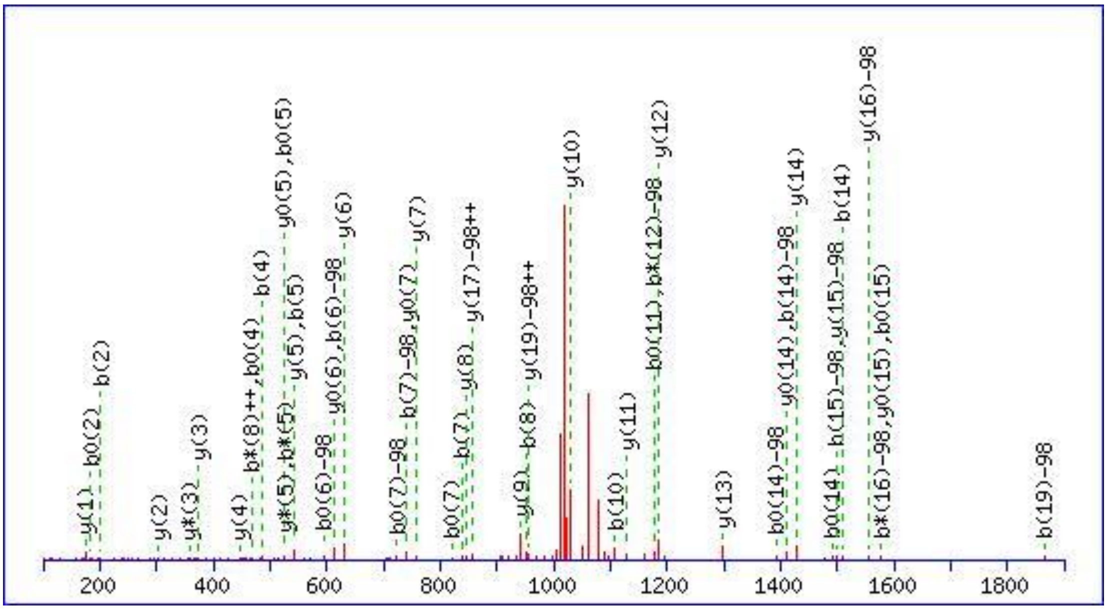

Monoisotopic mass of neutral peptide Mr(calc): 2136.9324

Variable modifications:

S6 : Phospho (ST), with neutral losses 97.9769(shown in table), 0.0000

Ions Score: 133 Expect: 1.3e-010

Matches : 51/334 fragment ions using 52 most intense peaks (help)

#	b	b^{++}	b*	b^{*++}	b^0	b^{0++}	Seq.	y	y^{++}	y*	y^{*++}	y^0	y^{0++}	#
1	130.0499	65.5286			112.0393	56.5233	E							20
2	201.0870	101.0471			183.0764	92.0418	A	1910.9203	955.9638	1893.8937	947.4505	1892.9097	946.9585	19
3	330.1296	165.5684			312.1190	156.5631	E	1839.8831	920.4452	1822.8566	911.9319	1821.8726	911.4399	18
4	486.2307	243.6190	469.2041	235.1057	468.2201	234.6137	R	1710.8406	855.9239	1693.8140	847.4106	1692.8300	846.9186	17
5	543.2522	272.1297	526.2256	263.6164	525.2416	263.1244	G	1554.7394	777.8734	1537.7129	769.3601	1536.7289	768.8681	16
6	612.2736	306.6404	595.2471	298.1272	594.2630	297.6352	S	1497.7180	749.3626	1480.6914	740.8494	1479.7074	740.3573	15
7	741.3162	371.1617	724.2897	362.6485	723.3056	362.1565	E	1428.6965	714.8519	1411.6700	706.3386	1410.6859	705.8466	14
8	854.4003	427.7038	837.3737	419.1905	836.3897	418.6985	L	1299.6539	650.3306	1282.6274	641.8173	1281.6434	641.3253	13
9	911.4217	456.2145	894.3952	447.7012	893.4112	447.2092	G	1186.5699	593.7886	1169.5433	585.2753	1168.5593	584.7833	12

**10**	1010.4901	505.7487	993.4636	497.2354	992.4796	496.7434	**V**	***1129.5484***	565.2778	1112.5218	556.7646	1111.5378	556.2726	**11**
**11**	1097.5222	549.2647	1080.4956	540.7515	1079.5116	540.2594	**S**	***1030.4800***	515.7436	1013.4534	507.2304	1012.4694	506.7383	**10**
**12**	1194.5749	597.7911	1177.5484	589.2778	1176.5644	588.7858	**P**	***943.4480***	472.2276	926.4214	463.7143	925.4374	463.2223	**9**
**13**	1281.6070	641.3071	1264.5804	632.7938	1263.5964	632.3018	**S**	***846.3952***	423.7012	829.3686	415.1880	828.3846	414.6959	**8**
**14**	**1410.6496**	705.8284	1393.6230	697.3151	1392.6390	696.8231	**E**	***759.3632***	380.1852	742.3366	371.6719	741.3526	371.1799	**7**
**15**	**1497.6816**	749.3444	1480.6550	740.8312	1479.6710	740.3392	**S**	***630.3206***	315.6639	613.2940	307.1506	612.3100	306.6586	**6**
**16**	1594.7344	797.8708	1577.7078	789.3575	1576.7238	788.8655	**P**	***543.2885***	272.1479	526.2620	263.6346	525.2780	263.1426	**5**
**17**	1665.7715	833.3894	1648.7449	824.8761	1647.7609	824.3841	**A**	***446.2358***	223.6215	429.2092	215.1083	428.2252	214.6162	**4**
**18**	1736.8086	868.9079	1719.7820	860.3947	1718.7980	859.9026	**A**	***375.1987***	188.1030	358.1721	179.5897	357.1881	179.0977	**3**
**19**	**1865.8512**	933.4292	1848.8246	924.9160	1847.8406	924.4239	**E**	***304.1615***	152.5844	287.1350	144.0711	286.1510	143.5791	**2**
**20**							**R**	***175.1190***	88.0631	158.0924	79.5498			**1**

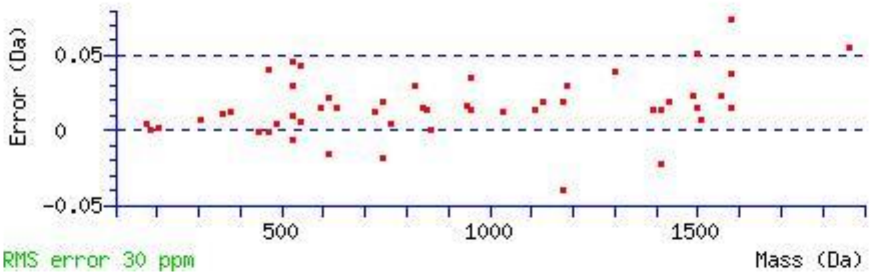

4_MS/MS Fragmentation of **GSELGVSPSESPAAER**

Found in **O95180**, Voltage-dependent T-type calcium channel subunit alpha-1H OS=Homo sapiens GN=CACNA1H PE=1 SV=4

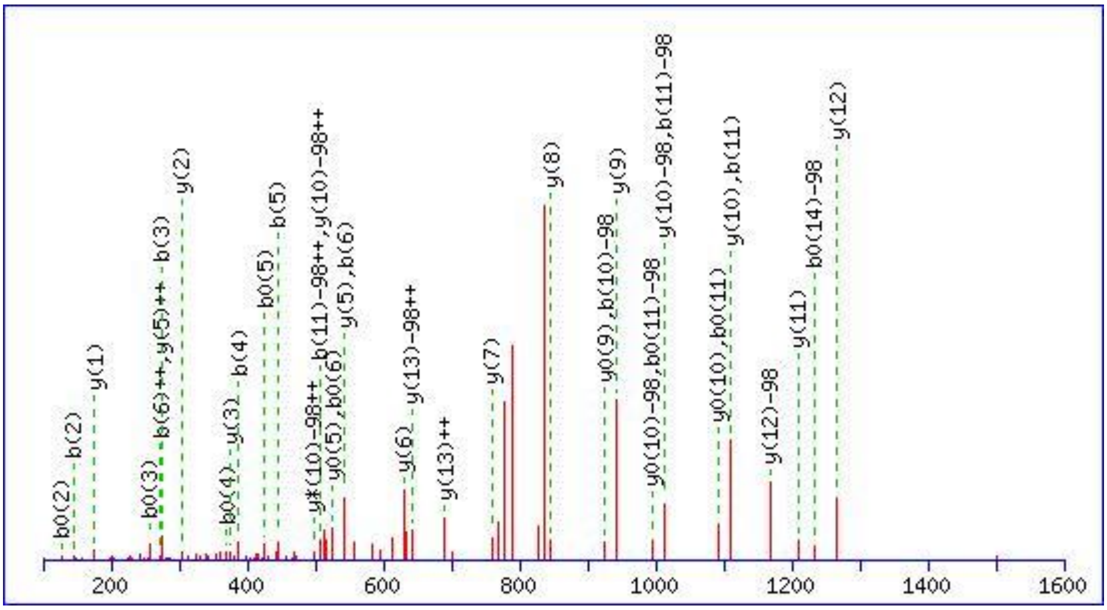

Monoisotopic mass of neutral peptide Mr(calc): 1651.7090

Variable modifications:

S7 : Phospho (ST), with neutral losses 0.0000(shown in table), 97.9769

Ions Score: 86 Expect: 6.6e-006

Matches : 40/218 fragment ions using 42 most intense peaks (help)

#	b	b^{++}	b^{0}	b^{0++}	Seq.	y	y^{++}	y*	y^{*++}	y^{0}	y^{0++}	#
1	58.0287	29.5180			G							16
2	**145.0608**	73.0340	127.0502	64.0287	S	1595.6949	798.3511	1578.6683	789.8378	1577.6843	789.3458	15
3	**274.1034**	137.5553	256.0928	128.5500	E	1508.6628	754.8351	1491.6363	746.3218	1490.6523	745.8298	14
4	**387.1874**	194.0974	369.1769	185.0921	L	1379.6203	690.3138	1362.5937	681.8005	1361.6097	681.3085	13
5	**444.2089**	222.6081	426.1983	213.6028	G	***1266.5362***	633.7717	1249.5096	625.2585	1248.5256	624.7664	12
6	**543.2773**	272.1423	525.2667	263.1370	V	***1209.5147***	605.2610	1192.4882	596.7477	1191.5042	596.2557	11
7	710.2757	355.6415	692.2651	346.6362	S	***1110.4463***	555.7268	1093.4198	547.2135	1092.4357	546.7215	10
8	807.3284	404.1679	789.3179	395.1626	P	***943.4480***	472.2276	926.4214	463.7143	925.4374	463.2223	9
9	894.3605	447.6839	876.3499	438.6786	S	***846.3952***	423.7012	829.3686	415.1880	828.3846	414.6959	8
10	1023.4030	512.2052	1005.3925	503.1999	E	***759.3632***	380.1852	742.3366	371.6719	741.3526	371.1799	7
11	**1110.4351**	555.7212	1092.4245	546.7159	S	***630.3206***	315.6639	613.2940	307.1506	612.3100	306.6586	6
12	1207.4878	604.2476	1189.4773	595.2423	P	***543.2885***	272.1479	526.2620	263.6346	525.2780	263.1426	5
13	1278.5250	639.7661	1260.5144	630.7608	A	446.2358	223.6215	429.2092	215.1083	428.2252	214.6162	4
14	1349.5621	675.2847	1331.5515	666.2794	A	***375.1987***	188.1030	358.1721	179.5897	357.1881	179.0977	3
15	1478.6047	739.8060	1460.5941	730.8007	E	***304.1615***	152.5844	287.1350	144.0711	286.1510	143.5791	2
16					R	***175.1190***	88.0631	158.0924	79.5498			1

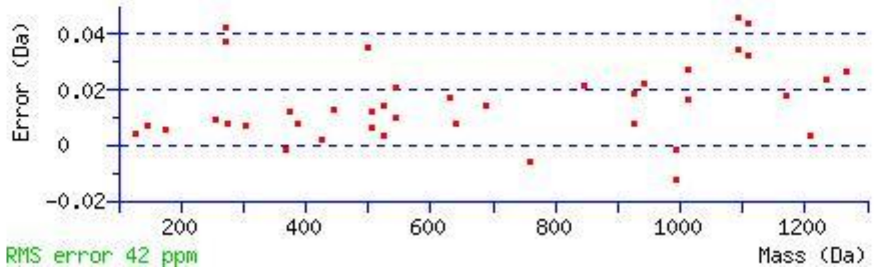
Error (Da)
0.04
0.02
0
-0.02
200
400
600
800
1000
1200
Mass (Da)
RMS error 42 ppm

5_MS/MS Fragmentation of **GSELGVSPSESPAAER**

Found in **O95180**, Voltage-dependent T-type calcium channel subunit alpha-1H OS=Homo sapiens GN=CACNA1H PE=1 SV=4

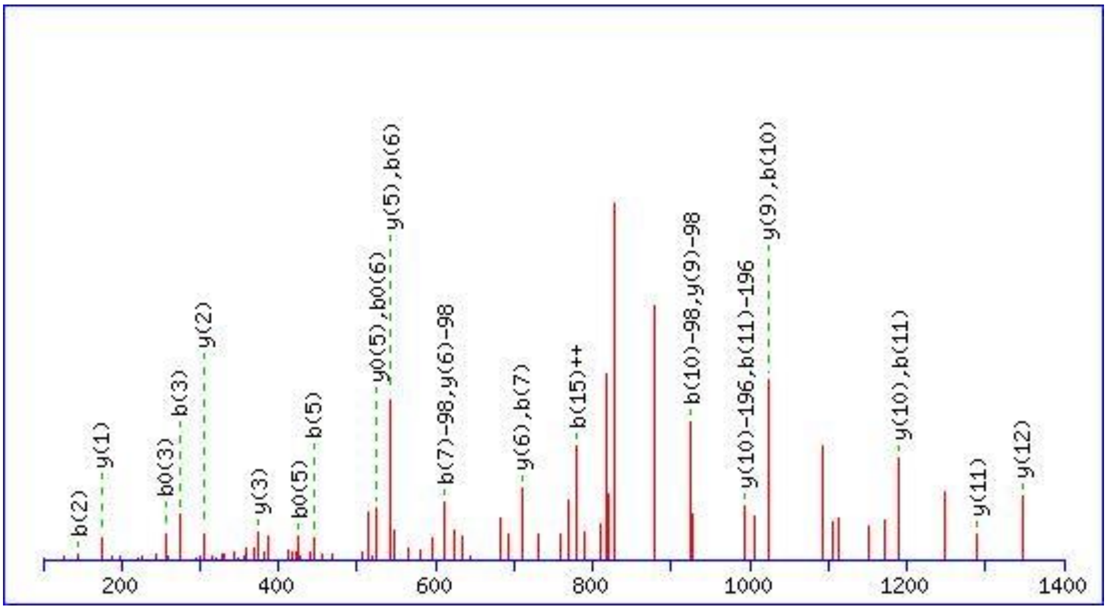

Monoisotopic mass of neutral peptide Mr(calc): 1731.6754

Variable modifications:

S7 : Phospho (ST), with neutral losses 0.0000(shown in table), 97.9769

S11 : Phospho (ST), with neutral losses 0.0000(shown in table), 97.9769

Ions Score: 75 Expect: 6.2e-005

Matches : 27/242 fragment ions using 25 most intense peaks (help)

#	b	b^{++}	b^{0}	b^{0++}	Seq.	y	y^{++}	y*	y^{*++}	y^{0}	y^{0++}	#
1	58.0287	29.5180			G							16
2	145.0608	73.0340	127.0502	64.0287	S	1675.6612	838.3342	1658.6347	829.8210	1657.6506	829.3290	15
3	274.1034	137.5553	256.0928	128.5500	E	1588.6292	794.8182	1571.6026	786.3050	1570.6186	785.8129	14
4	387.1874	194.0974	369.1769	185.0921	L	1459.5866	730.2969	1442.5600	721.7837	1441.5760	721.2916	13
5	444.2089	222.6081	426.1983	213.6028	G	1346.5025	673.7549	1329.4760	665.2416	1328.4920	664.7496	12
6	543.2773	272.1423	525.2667	263.1370	V	1289.4811	645.2442	1272.4545	636.7309	1271.4705	636.2389	11
7	710.2757	355.6415	692.2651	346.6362	S	1190.4126	595.7100	1173.3861	587.1967	1172.4021	586.7047	10
8	807.3284	404.1679	789.3179	395.1626	P	1023.4143	512.2108	1006.3877	503.6975	1005.4037	503.2055	9
9	894.3605	447.6839	876.3499	438.6786	S	926.3615	463.6844	909.3350	455.1711	908.3510	454.6791	8
10	1023.4030	512.2052	1005.3925	503.1999	E	839.3295	420.1684	822.3029	411.6551	821.3189	411.1631	7
11	1190.4014	595.7043	1172.3908	586.6991	S	710.2869	355.6471	693.2603	347.1338	692.2763	346.6418	6
12	1287.4542	644.2307	1269.4436	635.2254	P	543.2885	272.1479	526.2620	263.6346	525.2780	263.1426	5
13	1358.4913	679.7493	1340.4807	670.7440	A	446.2358	223.6215	429.2092	215.1083	428.2252	214.6162	4
14	1429.5284	715.2678	1411.5178	706.2626	A	375.1987	188.1030	358.1721	179.5897	357.1881	179.0977	3
15	1558.5710	779.7891	1540.5604	770.7839	E	304.1615	152.5844	287.1350	144.0711	286.1510	143.5791	2
16					R	175.1190	88.0631	158.0924	79.5498			1

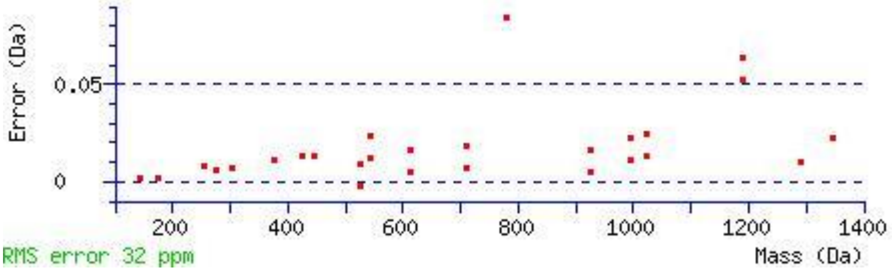

RMS error 32 ppm

6_MS/MS Fragmentation of **EAERGSELGVSPSESPAAER**

Found in **O95180**, Voltage-dependent T-type calcium channel subunit alpha-1H OS=Homo sapiens GN=CACNA1H PE=1 SV=4

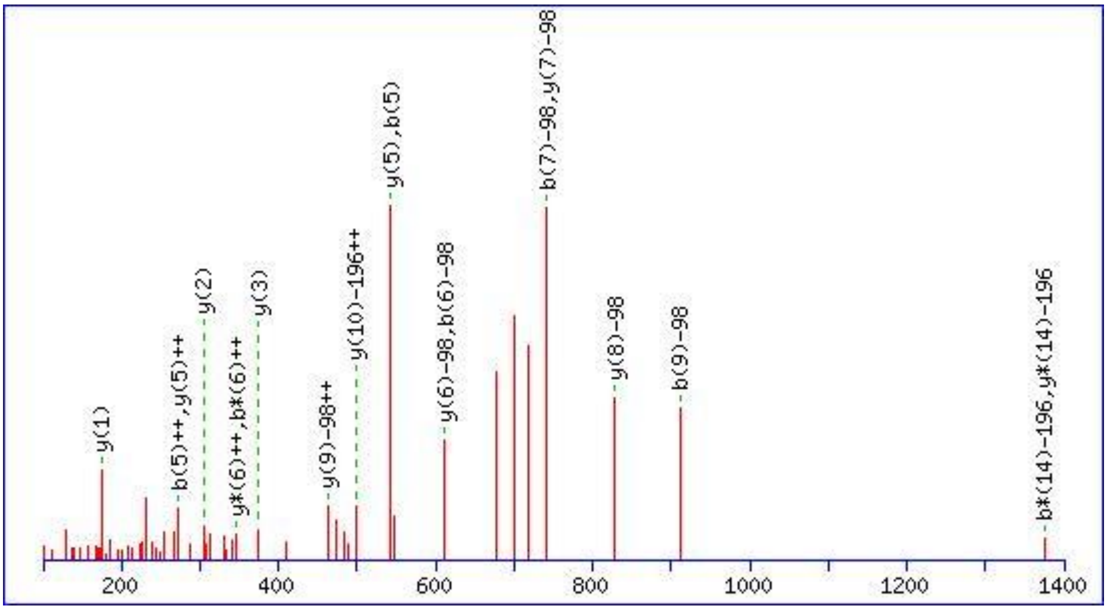

Monoisotopic mass of neutral peptide Mr(calc): 2296.8651

Variable modifications:

S6 : Phospho (ST), with neutral losses 97.9769(shown in table), 0.0000

S11 : Phospho (ST), with neutral losses 97.9769(shown in table), 0.0000

S15 : Phospho (ST), with neutral losses 97.9769(shown in table), 0.0000

Ions Score: 36 Expect: 0.41

Matches : 19/388 fragment ions using 22 most intense peaks (help)

#	b	b^{++}	b*	b^{*++}	b^0	b^{0++}	Seq.	y	y^{++}	y*	y^{*++}	y^0	y^{0++}	#
1	130.0499	65.5286			112.0393	56.5233	E							20
2	201.0870	101.0471			183.0764	92.0418	A	1874.8991	937.9532	1857.8726	929.4399	1856.8886	928.9479	19
3	330.1296	165.5684			312.1190	156.5631	E	1803.8620	902.4346	1786.8355	893.9214	1785.8514	893.4294	18
4	486.2307	243.6190	469.2041	235.1057	468.2201	234.6137	R	1674.8194	837.9133	1657.7929	829.4001	1656.8089	828.9081	17
5	543.2522	272.1297	526.2256	263.6164	525.2416	263.1244	G	1518.7183	759.8628	1501.6918	751.3495	1500.7077	750.8575	16
6	612.2736	306.6404	595.2471	298.1272	594.2630	297.6352	S	1461.6968	731.3521	1444.6703	722.8388	1443.6863	722.3468	15
7	741.3162	371.1617	724.2897	362.6485	723.3056	362.1565	E	1392.6754	696.8413	1375.6488	688.3281	1374.6648	687.8360	14
8	854.4003	427.7038	837.3737	419.1905	836.3897	418.6985	L	1263.6328	632.3200	1246.6062	623.8068	1245.6222	623.3148	13
9	911.4217	456.214	894.3952	447.701	893.4112	447.209	G	1150.548	575.778	1133.522	567.264	1132.538	566.772	1

		5		2		2		7	0	2	7	2	7	**2**
**10**	1010.4901	505.7487	993.4636	497.2354	992.4796	496.7434	**V**	1093.5273	547.2673	1076.5007	538.7540	1075.5167	538.2620	**11**
**11**	1079.5116	540.2594	1062.4851	531.7462	1061.5010	531.2542	**S**	994.4588	497.7331	977.4323	489.2198	976.4483	488.7278	**10**
**12**	1176.5644	588.7858	1159.5378	580.2726	1158.5538	579.7805	**P**	925.4374	463.2223	908.4108	454.7091	907.4268	454.2170	**9**
**13**	1263.5964	632.3018	1246.5699	623.7886	1245.5858	623.2966	**S**	***828.3846***	414.6959	811.3581	406.1827	810.3741	405.6907	**8**
**14**	1392.6390	696.8231	1375.6124	688.3099	1374.6284	687.8179	**E**	***741.3526***	371.1799	724.3260	362.6667	723.3420	362.1747	**7**
**15**	1461.6605	731.3339	1444.6339	722.8206	1443.6499	722.3286	**S**	***612.3100***	306.6586	595.2835	298.1454	594.2994	297.6534	**6**
**16**	1558.7132	779.8603	1541.6867	771.3470	1540.7027	770.8550	**P**	***543.2885***	272.1479	526.2620	263.6346	525.2780	263.1426	**5**
**17**	1629.7503	815.3788	1612.7238	806.8655	1611.7398	806.3735	**A**	446.2358	223.6215	429.2092	215.1083	428.2252	214.6162	**4**
**18**	1700.7875	850.8974	1683.7609	842.3841	1682.7769	841.8921	**A**	***375.1987***	188.1030	358.1721	179.5897	357.1881	179.0977	**3**
**19**	1829.8300	915.4187	1812.8035	906.9054	1811.8195	906.4134	**E**	***304.1615***	152.5844	287.1350	144.0711	286.1510	143.5791	**2**
**20**							**R**	***175.1190***	88.0631	158.0924	79.5498			**1**

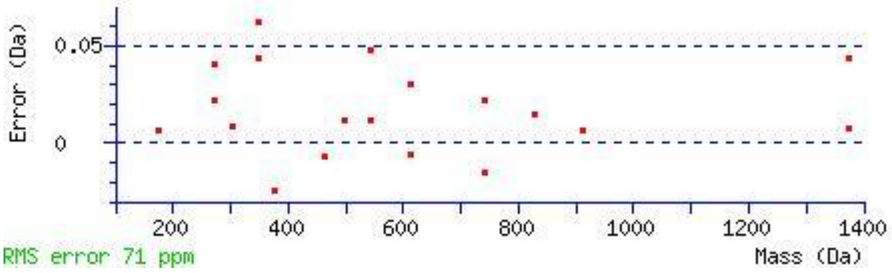

7_MS/MS Fragmentation of **HLSNDSTLASFSEPGSCYEELLK**

Found in **O95180**, Voltage-dependent T-type calcium channel subunit alpha-1H OS=Homo sapiens GN=CACNA1H PE=1 SV=4

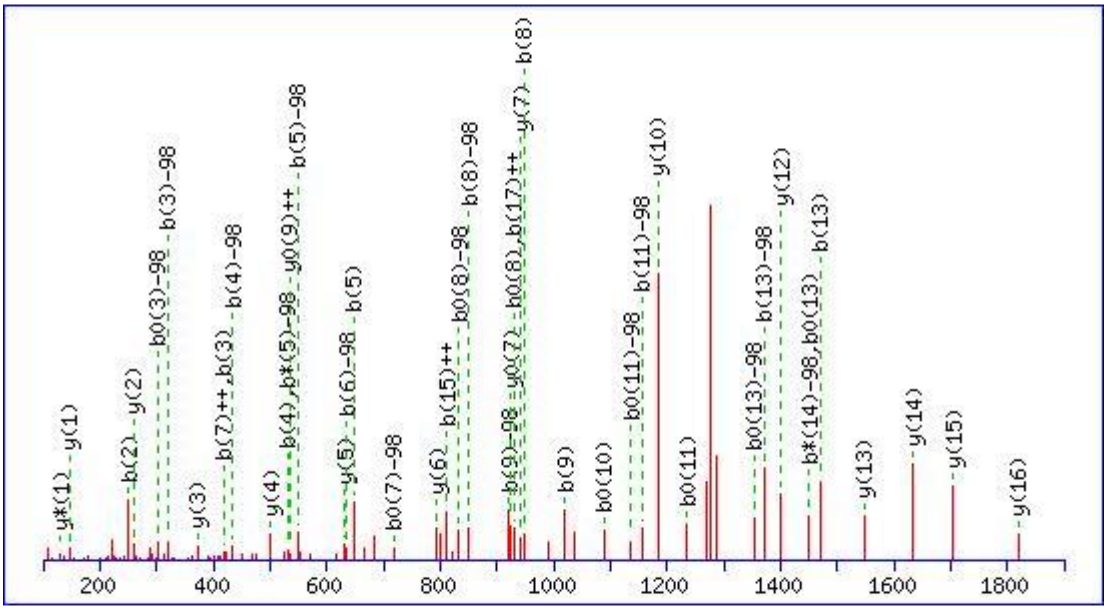

Monoisotopic mass of neutral peptide Mr(calc): 2652.1124

Variable modifications:

S3 : Phospho (ST), with neutral losses 97.9769(shown in table), 0.0000

C17 : Methylthio (C)

Ions Score: 84 Expect: 1.2e-005

Matches : 45/378 fragment ions using 61 most intense peaks (help)

#	b	b^{++}	b*	b*$^{++}$	b^{0}	b^{0++}	Seq.	y	y^{++}	y*	y*$^{++}$	y^{0}	y^{0++}	#
1	138.0662	69.5367					H							23
2	251.1503	126.0788					L	2418.0840	1209.5456	2401.0574	1201.0323	2400.0734	1200.5403	22
3	320.1717	160.5895			302.1611	151.5842	S	2304.9999	1153.0036	2287.9733	1144.4903	2286.9893	1143.9983	21
4	434.2146	217.6110	417.1881	209.0977	416.2041	208.6057	N	2235.9784	1118.4929	2218.9519	1109.9796	2217.9679	1109.4876	20
5	549.2416	275.1244	532.2150	266.6112	531.2310	266.1191	D	2121.9355	1061.4714	2104.9090	1052.9581	2103.9249	1052.4661	19
6	636.2736	318.6404	619.2471	310.1272	618.2630	309.6352	S	2006.9086	1003.9579	1989.8820	995.4446	1988.8980	994.9526	18
7	737.3213	369.1643	720.2947	360.6510	719.3107	360.1590	T	1919.8765	960.4419	1902.8500	951.9286	1901.8660	951.4366	17
8	850.4054	425.7063	833.3788	417.1930	832.3948	416.7010	L	1818.8289	909.9181	1801.8023	901.4048	1800.8183	900.9128	16
9	921.4425	461.2249	904.4159	452.7116	903.4319	452.2196	A	1705.7448	853.3760	1688.7182	844.8628	1687.7342	844.3707	15

**10**	1008.4745	504.7409	991.4479	496.2276	990.4639	495.7356	**S**	***1634.7077***	817.8575	1617.6811	809.3442	1616.6971	808.8522	**14**
**11**	***1155.5429***	578.2751	1138.5164	569.7618	1137.5323	569.2698	**F**	***1547.6756***	774.3415	1530.6491	765.8282	1529.6651	765.3362	**13**
**12**	1242.5749	621.7911	1225.5484	613.2778	1224.5644	612.7858	**S**	***1400.6072***	700.8073	1383.5807	692.2940	1382.5967	691.8020	**12**
**13**	***1371.6175***	686.3124	1354.5910	677.7991	1353.6070	677.3071	**E**	1313.5752	657.2912	1296.5487	648.7780	1295.5646	648.2860	**11**
**14**	1468.6703	734.8388	1451.6437	726.3255	1450.6597	725.8335	**P**	***1184.5326***	592.7699	1167.5061	584.2567	1166.5220	583.7647	**10**
**15**	1525.6918	763.3495	1508.6652	754.8362	1507.6812	754.3442	**G**	1087.4798	544.2436	1070.4533	535.7303	1069.4693	535.2383	**9**
**16**	1612.7238	806.8655	1595.6972	798.3523	1594.7132	797.8603	**S**	1030.4584	515.7328	1013.4318	507.2196	1012.4478	506.7275	**8**
**17**	1761.7207	881.3640	1744.6941	872.8507	1743.7101	872.3587	**C**	***943.4264***	472.2168	926.3998	463.7035	925.4158	463.2115	**7**
**18**	1924.7840	962.8957	1907.7575	954.3824	1906.7735	953.8904	**Y**	***794.4294***	397.7184	777.4029	389.2051	776.4189	388.7131	**6**
**19**	2053.8266	1027.4169	2036.8001	1018.9037	2035.8161	1018.4117	**E**	***631.3661***	316.1867	614.3396	307.6734	613.3556	307.1814	**5**
**20**	2182.8692	1091.9382	2165.8427	1083.4250	2164.8586	1082.9330	**E**	***502.3235***	251.6654	485.2970	243.1521	484.3130	242.6601	**4**
**21**	2295.9533	1148.4803	2278.9267	1139.9670	2277.9427	1139.4750	**L**	***373.2809***	187.1441	356.2544	178.6308			**3**
**22**	2409.0373	1205.0223	2392.0108	1196.5090	2391.0268	1196.0170	**L**	***260.1969***	130.6021	243.1703	122.0888			**2**
**23**							**K**	***147.1128***	74.0600	130.0863	65.5468			**1**

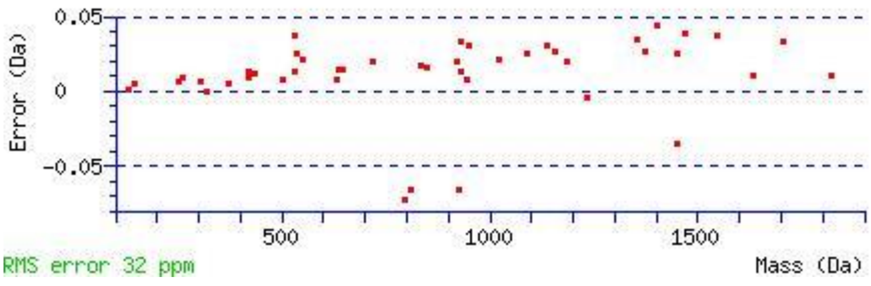

8_MS/MS Fragmentation of **AGAPPSPPSPGR**

Found in **O95180**, Voltage-dependent T-type calcium channel subunit alpha-1H OS=Homo sapiens GN=CACNA1H PE=1 SV=4

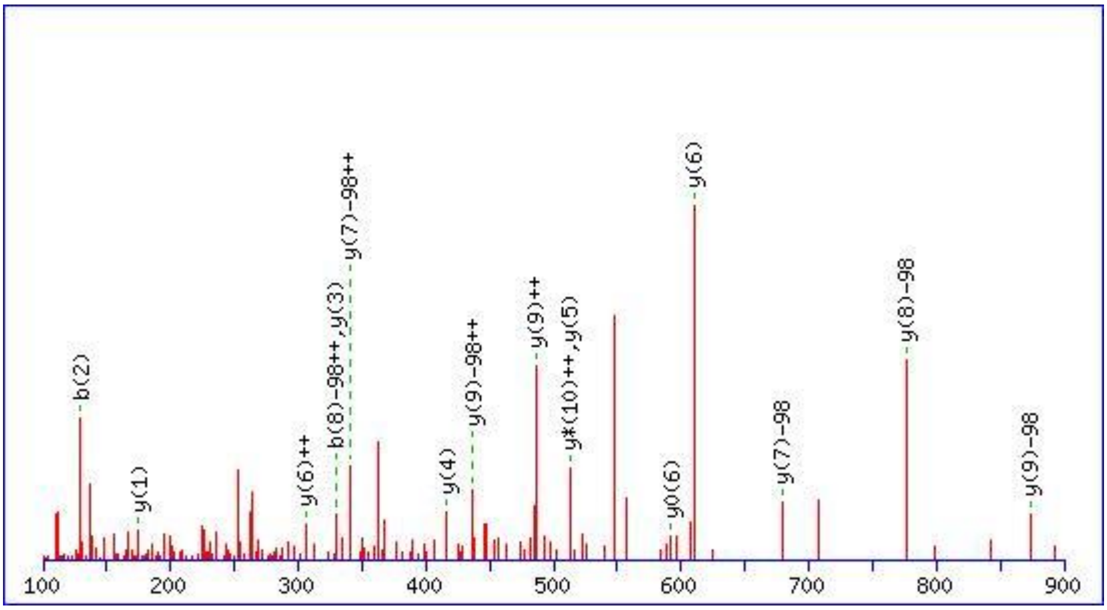

Monoisotopic mass of neutral peptide Mr(calc): 1169.5230

Variable modifications:

S6 : Phospho (ST), with neutral losses 97.9769(shown in table), 0.0000

Ions Score: 37 Expect: 0.35

Matches : 16/148 fragment ions using 35 most intense peaks (help)

#	b	b^{++}	b^0	b^{0++}	Seq.	y	y^{++}	y*	y^{*++}	y^0	y^{0++}	#
1	72.0444	36.5258			A							12
2	129.0659	65.0366			G	1001.5163	501.2618	984.4898	492.7485	983.5057	492.2565	11
3	200.1030	100.5551			A	944.4948	472.7511	927.4683	464.2378	926.4843	463.7458	10
4	297.1557	149.0815			P	***873.4577***	437.2325	856.4312	428.7192	855.4472	428.2272	9
5	394.2085	197.6079			P	***776.4050***	388.7061	759.3784	380.1928	758.3944	379.7008	8
6	463.2300	232.1186	445.2194	223.1133	S	***679.3522***	340.1797	662.3257	331.6665	661.3416	331.1745	7
7	560.2827	280.6450	542.2722	271.6397	P	***610.3307***	305.6690	593.3042	297.1557	592.3202	296.6637	6
8	657.3355	329.1714	639.3249	320.1661	P	***513.2780***	257.1426	496.2514	248.6293	495.2674	248.1373	5
9	744.3675	372.6874	726.3569	363.6821	S	***416.2252***	208.6162	399.1987	200.1030	398.2146	199.6110	4
10	841.4203	421.2138	823.4097	412.2085	P	***329.1932***	165.1002	312.1666	156.5870			3
11	898.4417	449.7245	880.4312	440.7192	G	232.1404	116.5738	215.1139	108.0606			2
12					R	***175.1190***	88.0631	158.0924	79.5498			1

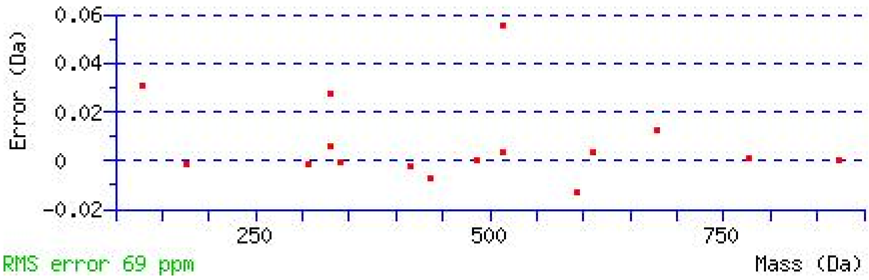

9_MS/MS Fragmentation of **AGAPPSPPSPGR**

Found in **O95180**, Voltage-dependent T-type calcium channel subunit alpha-1H OS=Homo sapiens GN=CACNA1H PE=1 SV=4

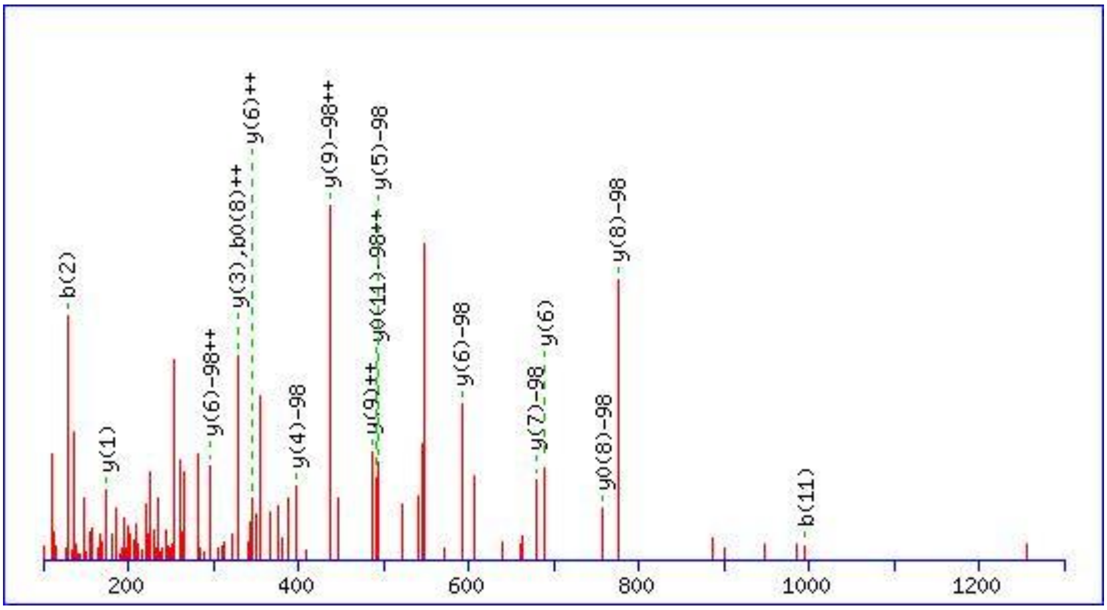

Monoisotopic mass of neutral peptide Mr(calc): 1169.5230

Variable modifications:

S9 : Phospho (ST), with neutral losses 97.9769(shown in table), 0.0000

Ions Score: 31 Expect: 1.7

Matches : 17/154 fragment ions using 31 most intense peaks (help)

#	b	b^{++}	b^{0}	b^{0++}	Seq.	y	y^{++}	y*	y^{*++}	y^{0}	y^{0++}	#
1	72.0444	36.5258			A							12
2	129.0659	65.0366			G	1001.5163	501.2618	984.4898	492.7485	983.5057	492.2565	11
3	200.1030	100.5551			A	944.4948	472.7511	927.4683	464.2378	926.4843	463.7458	10
4	297.1557	149.0815			P	873.4577	437.2325	856.4312	428.7192	855.4472	428.2272	9
5	394.2085	197.6079			P	***776.4050***	388.7061	759.3784	380.1928	758.3944	379.7008	8
6	481.2405	241.1239	463.2300	232.1186	S	***679.3522***	340.1797	662.3257	331.6665	661.3416	331.1745	7
7	578.2933	289.6503	560.2827	280.6450	P	***592.3202***	296.6637	575.2936	288.1504	574.3096	287.6584	6
8	675.3461	338.1767	657.3355	329.1714	P	***495.2674***	248.1373	478.2409	239.6241	477.2568	239.1321	5
9	744.3675	372.6874	726.3569	363.6821	S	***398.2146***	199.6110	381.1881	191.0977	380.2041	190.6057	4
10	841.4203	421.2138	823.4097	412.2085	P	***329.1932***	165.1002	312.1666	156.5870			3
11	898.4417	449.7245	880.4312	440.7192	G	232.1404	116.5738	215.1139	108.0606			2
12					R	***175.1190***	88.0631	158.0924	79.5498			1

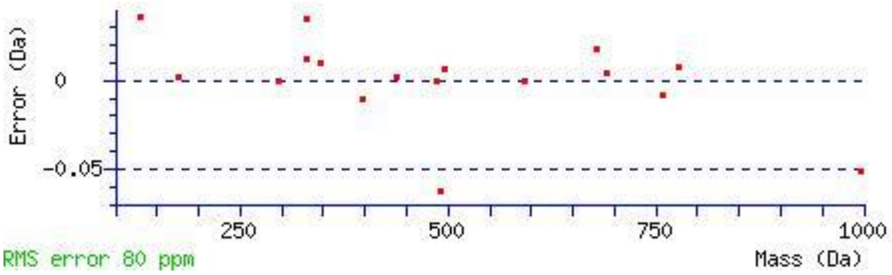

10_MS/MS Fragmentation of **AGAPPSPPSPGR**

Found in **O95180**, Voltage-dependent T-type calcium channel subunit alpha-1H OS=Homo sapiens GN=CACNA1H PE=1 SV=4

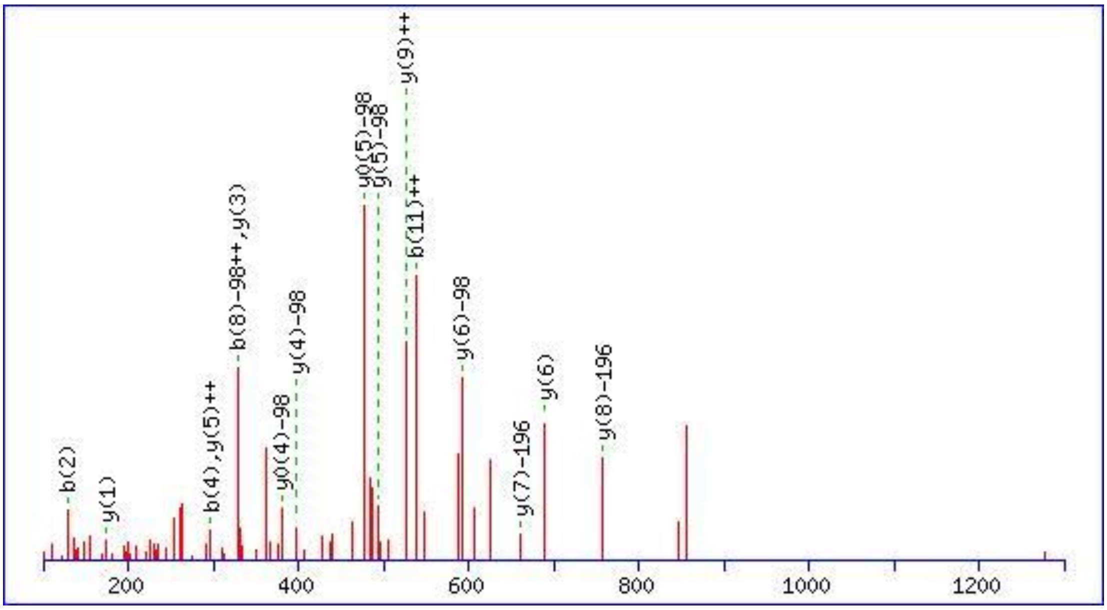

Monoisotopic mass of neutral peptide Mr(calc): 1249.4893
Variable modifications:
S6 : Phospho (ST), with neutral losses 97.9769(shown in table), 0.0000
S9 : Phospho (ST), with neutral losses 97.9769(shown in table), 0.0000
Ions Score: 35 Expect: 0.52
Matches : 16/166 fragment ions using 27 most intense peaks (help)

#	b	b^{++}	b^{0}	b^{0++}	Seq.	y	y^{++}	y*	y^{*++}	y^{0}	y^{0++}	#
1	72.0444	36.5258			A							12
2	**129.0659**	65.0366			G	983.5057	492.2565	966.4792	483.7432	965.4952	483.2512	11
3	200.1030	100.5551			A	926.4843	463.7458	909.4577	455.2325	908.4737	454.7405	10
4	**297.1557**	149.0815			P	855.4472	428.2272	838.4206	419.7139	837.4366	419.2219	9
5	394.2085	197.6079			P	***758.3944***	379.7008	741.3678	371.1876	740.3838	370.6956	8
6	463.2300	232.1186	445.2194	223.1133	S	***661.3416***	331.1745	644.3151	322.6612	643.3311	322.1692	7
7	560.2827	280.6450	542.2722	271.6397	P	***592.3202***	296.6637	575.2936	288.1504	574.3096	287.6584	6
8	657.3355	**329.1714**	639.3249	320.1661	P	***495.2674***	248.1373	478.2409	239.6241	477.2568	239.1321	5
9	726.3569	363.6821	708.3464	354.6768	S	***398.2146***	199.6110	381.1881	191.0977	380.2041	190.6057	4
10	823.4097	412.2085	805.3991	403.2032	P	***329.1932***	165.1002	312.1666	156.5870			3
11	880.4312	440.7192	862.4206	431.7139	G	232.1404	116.5738	215.1139	108.0606			2
12					R	***175.1190***	88.0631	158.0924	79.5498			1

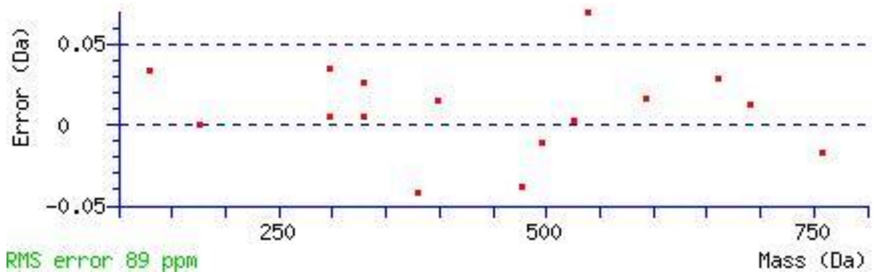
Error (Da)
0.05
0
-0.05
250
500
750
Mass (Da)
RMS error 89 ppm

11_MS/MS Fragmentation of **WAGGPPGTGGHGPLSLNSPDPYEK**

Found in **O95180**, Voltage-dependent T-type calcium channel subunit alpha-1H OS=Homo sapiens GN=CACNA1H PE=1 SV=4

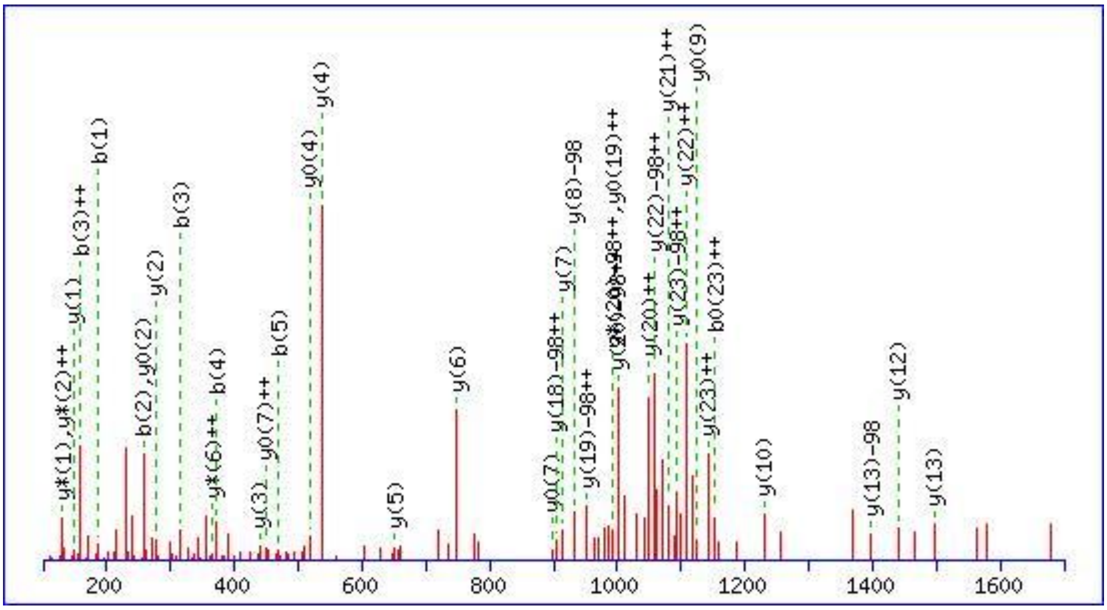

Monoisotopic mass of neutral peptide Mr(calc): 2470.0955

Variable modifications:

S18 : Phospho (ST), with neutral losses 0.0000(shown in table), 97.9769

Ions Score: 40 Expect: 0.3

Matches : 38/366 fragment ions using 81 most intense peaks (help)

#	b	b^{++}	b*	b^{*++}	b^{0}	b^{0++}	Seq.	y	y^{++}	y*	y^{*++}	y^{0}	y^{0++}	#
1	187.0866	94.0469					W							24
2	258.1237	129.5655					A	2285.0234	1143.0153	2267.9969	1134.5021	2267.0129	1134.0101	23
3	315.1452	158.0762					G	2213.9863	1107.4968	2196.9598	1098.9835	2195.9757	1098.4915	22
4	372.1666	186.5870					G	2156.9648	1078.9861	2139.9383	1070.4728	2138.9543	1069.9808	21
5	469.2194	235.1133					P	2099.9434	1050.4753	2082.9168	1041.9621	2081.9328	1041.4700	20
6	566.2722	283.6397					P	2002.8906	1001.9489	1985.8641	993.4357	1984.8800	992.9437	19
7	623.2936	312.1504					G	1905.8379	953.4226	1888.8113	944.9093	1887.8273	944.4173	18
8	724.3413	362.6743			706.3307	353.6690	T	1848.8164	924.9118	1831.7898	916.3986	1830.8058	915.9065	17
9	781.3628	391.1850			763.3522	382.1797	G	1747.7687	874.3880	1730.7422	865.8747	1729.7581	865.3827	16

10	838.3842	419.6958			820.3737	410.6905	G	1690.7472	845.8773	1673.7207	837.3640	1672.7367	836.8720	15
11	975.4431	488.2252			957.4326	479.2199	H	1633.7258	817.3665	1616.6992	808.8533	1615.7152	808.3612	14
12	1032.4646	516.7359			1014.4540	507.7307	G	***1496.6669***	748.8371	1479.6403	740.3238	1478.6563	739.8318	13
13	1129.5174	565.2623			1111.5068	556.2570	P	***1439.6454***	720.3263	1422.6189	711.8131	1421.6348	711.3211	12
14	1242.6014	621.8044			1224.5909	612.7991	L	1342.5926	671.8000	1325.5661	663.2867	1324.5821	662.7947	11
15	1329.6335	665.3204			1311.6229	656.3151	S	***1229.5086***	615.2579	1212.4820	606.7447	1211.4980	606.2526	10
16	1442.7175	721.8624			1424.7070	712.8571	L	1142.4765	571.7419	1125.4500	563.2286	1124.4660	562.7366	9
17	1556.7605	778.8839	1539.7339	770.3706	1538.7499	769.8786	N	1029.3925	515.1999	1012.3659	506.6866	1011.3819	506.1946	8
18	1723.7588	862.3830	1706.7323	853.8698	1705.7482	853.3778	S	***915.3496***	458.1784	898.3230	449.6651	897.3390	449.1731	7
19	1820.8116	910.9094	1803.7850	902.3962	1802.8010	901.9041	P	***748.3512***	374.6792	731.3246	366.1660	730.3406	365.6740	6
20	1935.8385	968.4229	1918.8120	959.9096	1917.8280	959.4176	D	***651.2984***	326.1529	634.2719	317.6396	633.2879	317.1476	5
21	2032.8913	1016.9493	2015.8647	1008.4360	2014.8807	1007.9440	P	***536.2715***	268.6394	519.2449	260.1261	518.2609	259.6341	4
22	2195.9546	1098.4809	2178.9281	1089.9677	2177.9440	1089.4757	Y	***439.2187***	220.1130	422.1922	211.5997	421.2082	211.1077	3
23	2324.9972	1163.0022	2307.9707	1154.4890	2306.9866	1153.9970	E	***276.1554***	138.5813	259.1288	130.0681	258.1448	129.5761	2
24							K	***147.1128***	74.0600	130.0863	65.5468			1

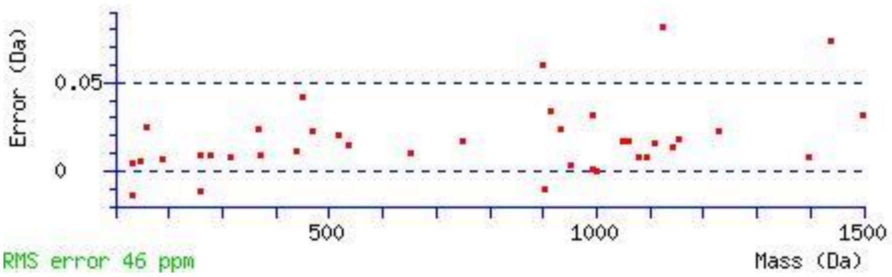

12_MS/MS Fragmentation of **ALEDPEGELSGSESGDSDGR**

Found in **O95180**, Voltage-dependent T-type calcium channel subunit alpha-1H OS=Homo sapiens GN=CACNA1H PE=1 SV=4

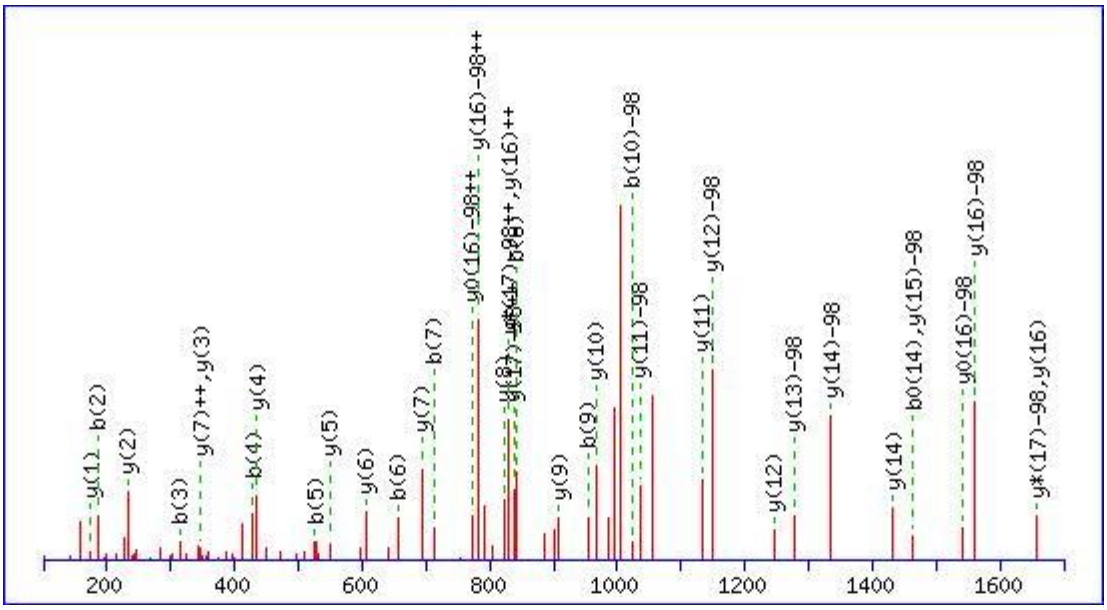

Monoisotopic mass of neutral peptide Mr(calc): 2085.8012

Variable modifications:

S10 : Phospho (ST), with neutral losses 97.9769(shown in table), 0.0000

Ions Score: 128 Expect: 2.7e-010

Matches : 38/276 fragment ions using 50 most intense peaks (help)

#	b	b^{++}	b^0	b^{0++}	Seq.	y	y^{++}	y*	y^{*++}	y^0	y^{0++}	#
1	72.0444	36.5258			A							20
2	185.1285	93.0679			L	1917.7944	959.4009	1900.7679	950.8876	1899.7839	950.3956	19
3	314.1710	157.5892	296.1605	148.5839	E	1804.7104	902.8588	1787.6838	894.3456	1786.6998	893.8535	18
4	429.1980	215.1026	411.1874	206.0974	D	1675.6678	838.3375	1658.6412	829.8243	1657.6572	829.3323	17
5	526.2508	263.6290	508.2402	254.6237	P	1560.6408	780.8241	1543.6143	772.3108	1542.6303	771.8188	16
6	655.2933	328.1503	637.2828	319.1450	E	1463.5881	732.2977	1446.5615	723.7844	1445.5775	723.2924	15
7	712.3148	356.6610	694.3042	347.6558	G	1334.5455	667.7764	1317.5189	659.2631	1316.5349	658.7711	14
8	841.3574	421.1823	823.3468	412.1771	E	1277.5240	639.2657	1260.4975	630.7524	1259.5135	630.2604	13
9	954.4415	477.7244	936.4309	468.7191	L	1148.4814	574.7444	1131.4549	566.2311	1130.4709	565.7391	12
10	1023.4629	512.2351	1005.4524	503.2298	S	1035.3974	518.2023	1018.3708	509.6890	1017.3868	509.1970	11
11	1080.4844	540.7458	1062.4738	531.7406	G	966.3759	483.6916	949.3494	475.1783	948.3653	474.6863	10
12	1167.5164	584.2618	1149.5059	575.2566	S	909.3544	455.1809	892.3279	446.6676	891.3439	446.1756	9
13	1296.5590	648.7831	1278.5485	639.7779	E	822.3224	411.6648	805.2959	403.1516	804.3119	402.6596	8
14	1383.5910	692.2992	1365.5805	683.2939	S	693.2798	347.1435	676.2533	338.6303	675.2693	338.1383	7
15	1440.6125	720.8099	1422.6019	711.8046	G	606.2478	303.6275	589.2212	295.1143	588.2372	294.6223	6
16	1555.6395	778.3234	1537.6289	769.3181	D	549.2263	275.1168	532.1998	266.6035	531.2158	266.1115	5
17	1642.6715	821.8394	1624.6609	812.8341	S	434.1994	217.6033	417.1728	209.0901	416.1888	208.5980	4

**18**	1757.6984	879.3528	1739.6879	870.3476	**D**	***347.1674***	174.0873	330.1408	165.5740	329.1568	165.0820	**3**
**19**	1814.7199	907.8636	1796.7093	898.8583	**G**	***232.1404***	116.5738	215.1139	108.0606			**2**
**20**					**R**	***175.1190***	88.0631	158.0924	79.5498			**1**

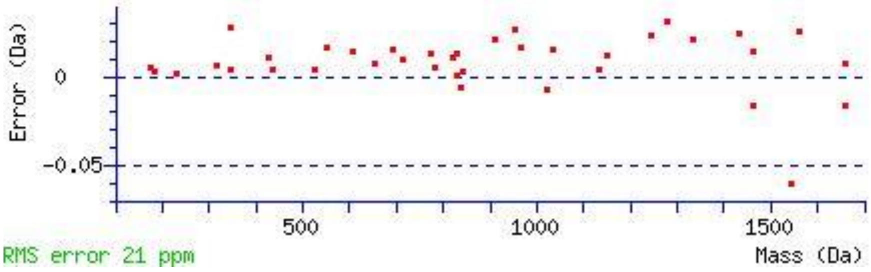

13_MS/MS Fragmentation of **ALEDPEGELSGSESGDSDGR**

Found in **O95180**, Voltage-dependent T-type calcium channel subunit alpha-1H OS=Homo sapiens GN=CACNA1H PE=1 SV=4

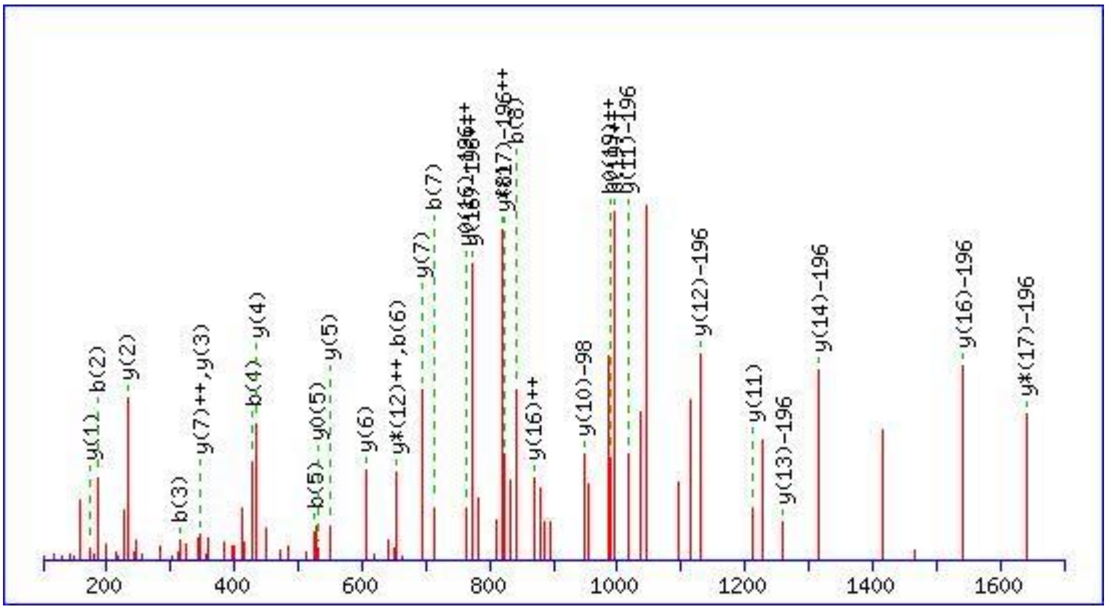

Monoisotopic mass of neutral peptide Mr(calc): 2165.7675

Variable modifications:

S10 : Phospho (ST), with neutral losses 97.9769(shown in table), 0.0000

S12 : Phospho (ST), with neutral losses 97.9769(shown in table), 0.0000

Ions Score: 93 Expect: 6.1e-007

Matches : 32/288 fragment ions using 50 most intense peaks (help)

#	b	b^{++}	b^0	b^{0++}	Seq.	y	y^{++}	y*	y^{*++}	y^0	y^{0++}	#
1	72.0444	36.5258			A							20
2	**185.1285**	93.0679			L	1899.7839	950.3956	1882.7573	941.8823	1881.7733	941.3903	19
3	**314.1710**	157.5892	296.1605	148.5839	E	1786.6998	893.8535	1769.6733	885.3403	1768.6893	884.8483	18
4	**429.1980**	215.1026	411.1874	206.0974	D	1657.6572	829.3323	1640.6307	820.8190	1639.6467	820.3270	17
5	**526.2508**	263.6290	508.2402	254.6237	P	***1542.6303***	771.8188	1525.6037	763.3055	1524.6197	762.8135	16
6	**655.2933**	328.1503	637.2828	319.1450	E	1445.5775	723.2924	1428.5510	714.7791	1427.5670	714.2871	15
7	**712.3148**	356.6610	694.3042	347.6558	G	***1316.5349***	658.7711	1299.5084	650.2578	1298.5244	649.7658	14
8	**841.3574**	421.1823	823.3468	412.1771	E	***1259.5135***	630.2604	1242.4869	621.7471	1241.5029	621.2551	13
9	954.4415	477.7244	936.4309	468.7191	L	***1130.4709***	565.7391	1113.4443	557.2258	1112.4603	556.7338	12
10	1023.4629	512.2351	1005.4524	503.2298	S	***1017.3868***	509.1970	1000.3603	500.6838	999.3762	500.1918	11
11	1080.4844	540.7458	1062.4738	531.7406	G	***948.3653***	474.6863	931.3388	466.1730	930.3548	465.6810	10
12	1149.5059	575.2566	1131.4953	566.2513	S	891.3439	446.1756	874.3173	437.6623	873.3333	437.1703	9
13	1278.5485	639.7779	1260.5379	630.7726	E	***822.3224***	411.6648	805.2959	403.1516	804.3119	402.6596	8
14	1365.5805	683.2939	1347.5699	674.2886	S	***693.2798***	347.1435	676.2533	338.6303	675.2693	338.1383	7
15	1422.6019	711.8046	1404.5914	702.7993	G	***606.2478***	303.6275	589.2212	295.1143	588.2372	294.6223	6
16	1537.6289	769.3181	1519.6183	760.3128	D	***549.2263***	275.1168	532.1998	266.6035	531.2158	266.1115	5
17	1624.6609	812.8341	1606.6503	803.8288	S	***434.1994***	217.6033	417.1728	209.0901	416.1888	208.5980	4

**18**	1739.6879	870.3476	1721.6773	861.3423	**D**	***347.1674***	174.0873	330.1408	165.5740	329.1568	165.0820	**3**
**19**	1796.7093	898.8583	1778.6988	889.8530	**G**	***232.1404***	116.5738	215.1139	108.0606			**2**
**20**					**R**	***175.1190***	88.0631	158.0924	79.5498			**1**

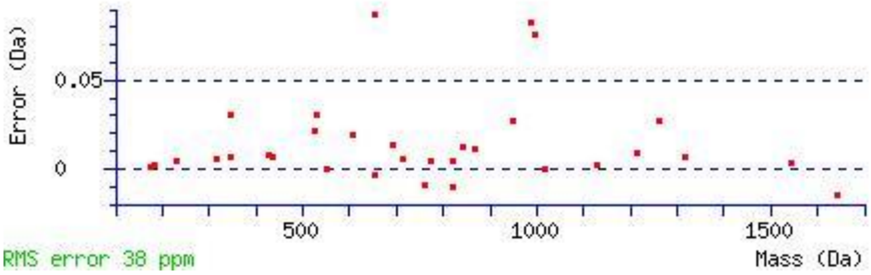

14_MS/MS Fragmentation of **ALEDPEGELSGSESGDSDGRGVYEFTQDVR**

Found in **O95180**, Voltage-dependent T-type calcium channel subunit alpha-1H OS=Homo sapiens GN=CACNA1H PE=1 SV=4

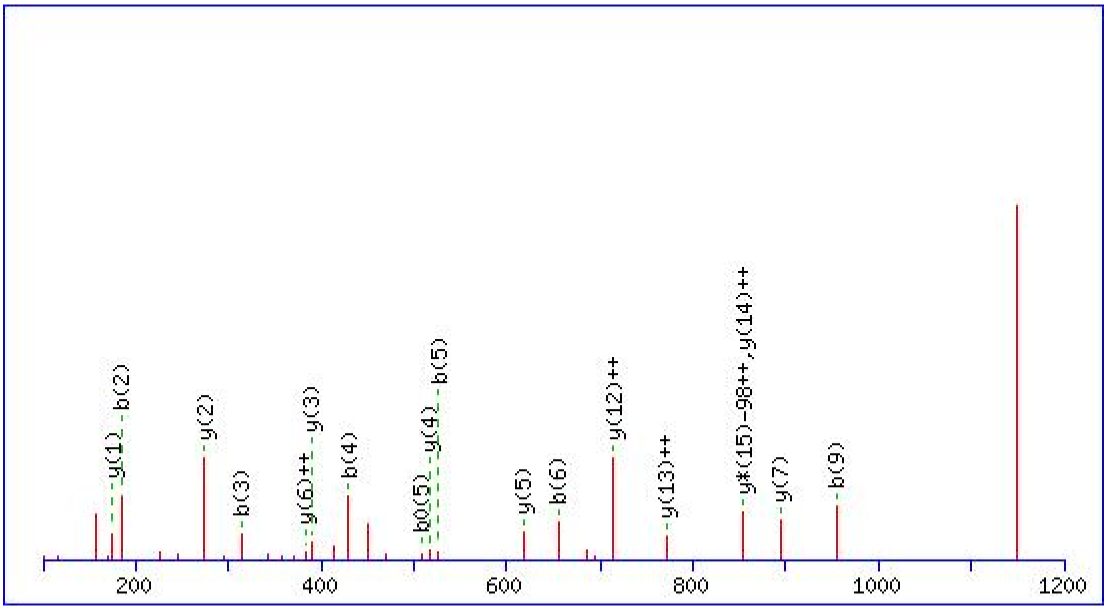

Monoisotopic mass of neutral peptide Mr(calc): 3520.2671

Variable modifications:

S10 : Phospho (ST), with neutral losses 0.0000(shown in table), 97.9769

S12 : Phospho (ST), with neutral losses 0.0000(shown in table), 97.9769

S14 : Phospho (ST), with neutral losses 0.0000(shown in table), 97.9769

S17 : Phospho (ST), with neutral losses 0.0000(shown in table), 97.9769

Ions Score: 54 Expect: 0.0059

Matches : 18/498 fragment ions using 24 most intense peaks (help)

#	b	b^{++}	b*	b^{*++}	b^0	b^{0++}	Seq.	y	y^{++}	y*	y^{*++}	y^0	y^{0++}	#
1	72.0444	36.5258					A							30
2	**185.1285**	93.0679					L	3450.2373	1725.6223	3433.2107	1717.1090	3432.2267	1716.6170	29
3	**314.1710**	157.5892			296.1605	148.5839	E	3337.1532	1669.0802	3320.1267	1660.5670	3319.1426	1660.0750	28
4	**429.1980**	215.1026			411.1874	206.0974	D	3208.1106	1604.5589	3191.0841	1596.0457	3190.1001	1595.5537	27
5	**526.2508**	263.6290			508.2402	254.6237	P	3093.0837	1547.0455	3076.0571	1538.5322	3075.0731	1538.0402	26
6	**655.2933**	328.1503			637.2828	319.1450	E	2996.0309	1498.5191	2979.0044	1490.0058	2978.0203	1489.5138	25
7	712.3148	356.6610			694.3042	347.6558	G	2866.9883	1433.9978	2849.9618	1425.4845	2848.9778	1424.9925	24
8	841.3574	421.1823			823.3468	412.1771	E	2809.9669	1405.4871	2792.9403	1396.9738	2791.9563	1396.4818	23

9	***954.4415***	477.7244			936.4309	468.7191	L	2680.9243	1340.9658	2663.8977	1332.4525	2662.9137	1331.9605	22
10	1121.4398	561.2236			1103.4293	552.2183	S	2567.8402	1284.4237	2550.8136	1275.9105	2549.8296	1275.4185	21
11	1178.4613	589.7343			1160.4507	580.7290	G	2400.8418	1200.9246	2383.8153	1192.4113	2382.8313	1191.9193	20
12	1345.4597	673.2335			1327.4491	664.2282	S	2343.8204	1172.4138	2326.7938	1163.9006	2325.8098	1163.4085	19
13	1474.5022	737.7548			1456.4917	728.7495	E	2176.8220	1088.9146	2159.7955	1080.4014	2158.8115	1079.9094	18
14	1641.5006	821.2539			1623.4900	812.2487	S	2047.7794	1024.3933	2030.7529	1015.8801	2029.7689	1015.3881	17
15	1698.5221	849.7647			1680.5115	840.7594	G	1880.7811	940.8942	1863.7545	932.3809	1862.7705	931.8889	16
16	1813.5490	907.2781			1795.5384	898.2729	D	1823.7596	912.3834	1806.7331	903.8702	1805.7490	903.3782	15
17	1980.5474	990.7773			1962.5368	981.7720	S	1708.7327	854.8700	1691.7061	846.3567	1690.7221	845.8647	14
18	2095.5743	1048.2908			2077.5637	1039.2855	D	1541.7343	771.3708	1524.7077	762.8575	1523.7237	762.3655	13
19	2152.5958	1076.8015			2134.5852	1067.7962	G	1426.7074	713.8573	1409.6808	705.3440	1408.6968	704.8520	12
20	2308.6969	1154.8521	2291.6703	1146.3388	2290.6863	1145.8468	R	1369.6859	685.3466	1352.6593	676.8333	1351.6753	676.3413	11
21	2365.7184	1183.3628	2348.6918	1174.8495	2347.7078	1174.3575	G	1213.5848	607.2960	1196.5582	598.7828	1195.5742	598.2907	10
22	2464.7868	1232.8970	2447.7602	1224.3837	2446.7762	1223.8917	V	1156.5633	578.7853	1139.5368	570.2720	1138.5528	569.7800	9
23	2627.8501	1314.4287	2610.8235	1305.9154	2609.8395	1305.4234	Y	1057.4949	529.2511	1040.4684	520.7378	1039.4843	520.2458	8
24	2756.8927	1378.9500	2739.8661	1370.4367	2738.8821	1369.9447	E	***894.4316***	447.7194	877.4050	439.2061	876.4210	438.7141	7
25	2903.9611	1452.4842	2886.9346	1443.9709	2885.9505	1443.4789	F	765.3890	383.1981	748.3624	374.6849	747.3784	374.1928	6
26	3005.0088	1503.0080	2987.9822	1494.4948	2986.9982	1494.0027	T	***618.3206***	309.6639	601.2940	301.1506	600.3100	300.6586	5
27	3133.0674	1567.0373	3116.0408	1558.5240	3115.0568	1558.0320	Q	***517.2729***	259.1401	500.2463	250.6268	499.2623	250.1348	4

**28**	3248.0943	1624.5508	3231.0678	1616.0375	3230.0837	1615.5455	**D**	***389.2143***	195.1108	372.1878	186.5975	371.2037	186.1055	**3**
**29**	3347.1627	1674.0850	3330.1362	1665.5717	3329.1521	1665.0797	**V**	***274.1874***	137.5973	257.1608	129.0840			**2**
**30**							**R**	***175.1190***	88.0631	158.0924	79.5498			**1**

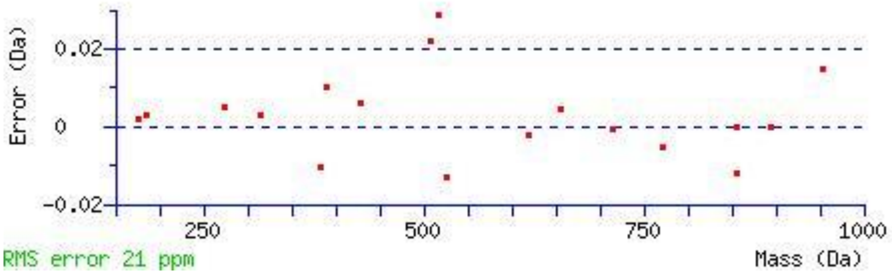

15_MS/MS Fragmentation of **ATDTPGPGPGSPQR**

Found in **O95180**, Voltage-dependent T-type calcium channel subunit alpha-1H OS=Homo sapiens GN=CACNA1H PE=1 SV=4

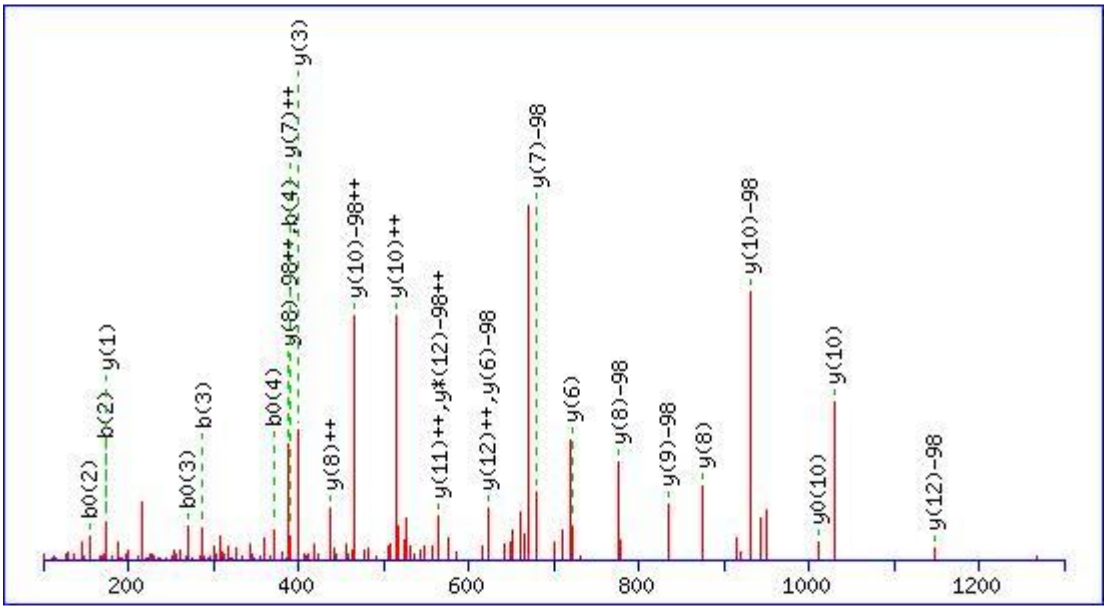

Monoisotopic mass of neutral peptide Mr(calc): 1416.6035

Variable modifications:

S11 : Phospho (ST), with neutral losses 97.9769(shown in table), 0.0000

Ions Score: 47 Expect: 0.039

Matches : 26/198 fragment ions using 30 most intense peaks (help)

#	b	b^{++}	b*	b^{*++}	b^{0}	b^{0++}	Seq.	y	y^{++}	y*	y^{*++}	y^{0}	y^{0++}	#
1	72.0444	36.5258					A							14
2	**173.0921**	87.0497			155.0815	78.0444	T	1248.5967	624.8020	1231.5702	616.2887	1230.5862	615.7967	13
3	**288.1190**	144.5631			270.1084	135.5579	D	***1147.5491***	574.2782	1130.5225	565.7649	1129.5385	565.2729	12
4	**389.1667**	195.0870			371.1561	186.0817	T	1032.5221	516.7647	1015.4956	508.2514	1014.5116	507.7594	11
5	486.2195	243.6134			468.2089	234.6081	P	***931.4744***	466.2409	914.4479	457.7276	913.4639	457.2356	10
6	543.2409	272.1241			525.2304	263.1188	G	***834.4217***	417.7145	817.3951	409.2012	816.4111	408.7092	9
7	640.2937	320.6505			622.2831	311.6452	P	***777.4002***	389.2037	760.3737	380.6905	759.3896	380.1985	8
8	697.3151	349.1612			679.3046	340.1559	G	***680.3474***	340.6774	663.3209	332.1641	662.3369	331.6721	7
9	794.3679	397.6876			776.3573	388.6823	P	***623.3260***	312.1666	606.2994	303.6534	605.3154	303.1613	6

**10**	851.3894	426.1983			833.3788	417.1930	**G**	526.2732	263.6402	509.2467	255.1270	508.2627	254.6350	**5**
**11**	920.4108	460.7091			902.4003	451.7038	**S**	469.2518	235.1295	452.2252	226.6162	451.2412	226.1242	**4**
**12**	1017.4636	509.2354			999.4530	500.2302	**P**	***400.2303***	200.6188	383.2037	192.1055			**3**
**13**	1145.5222	573.2647	1128.4956	564.7515	1127.5116	564.2594	**Q**	303.1775	152.0924	286.1510	143.5791			**2**
**14**							**R**	***175.1190***	88.0631	158.0924	79.5498			**1**

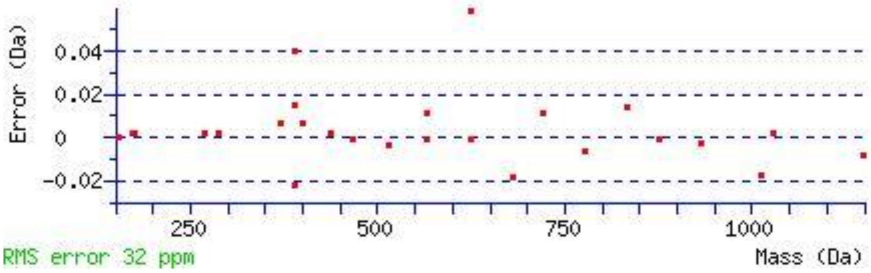

16_MS/MS Fragmentation of **SDTDEDKTSVHFEEDFHK**

Found in **O95180**, Voltage-dependent T-type calcium channel subunit alpha-1H OS=Homo sapiens GN=CACNA1H PE=1 SV=4

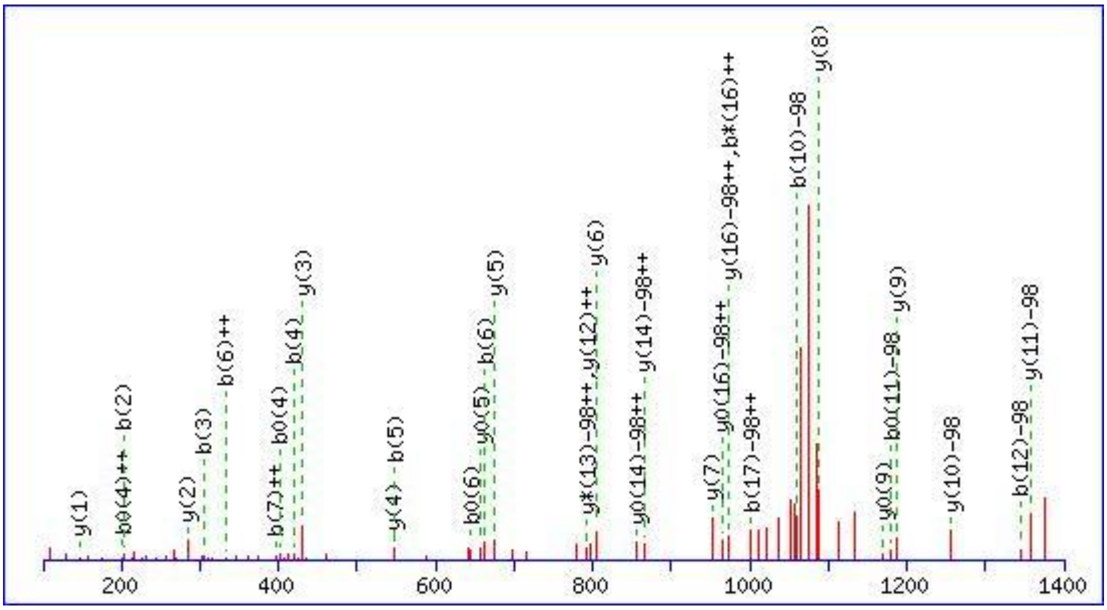

Monoisotopic mass of neutral peptide Mr(calc): 2244.8849

Variable modifications:

S9 : Phospho (ST), with neutral losses 97.9769(shown in table), 0.0000

Ions Score: 69 Expect: 0.00029

Matches : 34/288 fragment ions using 63 most intense peaks (help)

#	b	b^{++}	b*	b^{*++}	b^{0}	b^{0++}	Seq.	y	y^{++}	y*	y^{*++}	y^{0}	y^{0++}	#
1	88.0393	44.5233			70.0287	35.5180	S							18
2	***203.0662***	102.0368			185.0557	93.0315	D	2060.8832	1030.9452	2043.8567	1022.4320	2042.8726	1021.9400	17
3	***304.1139***	152.5606			286.1034	143.5553	T	1945.8563	973.4318	1928.8297	964.9185	1927.8457	964.4265	16
4	***419.1409***	210.0741			401.1303	201.0688	D	1844.8086	922.9079	1827.7820	914.3947	1826.7980	913.9026	15
5	***548.1835***	274.5954			530.1729	265.5901	E	1729.7816	865.3945	1712.7551	856.8812	1711.7711	856.3892	14
6	***663.2104***	**332.1088**			645.1998	323.1036	D	1600.7390	800.8732	1583.7125	792.3599	1582.7285	791.8679	13
7	791.3054	**396.1563**	774.2788	387.6430	773.2948	387.1510	K	1485.7121	743.3597	1468.6856	734.8464	1467.7015	734.3544	12
8	892.3530	446.6802	875.3265	438.1669	874.3425	437.6749	T	***1357.6171***	679.3122	1340.5906	670.7989	1339.6066	670.3069	11
9	961.3745	481.1909	944.3480	472.6776	943.3639	472.1856	S	***1256.5695***	628.7884	1239.5429	620.2751	1238.5589	619.7831	10

10	1060.4429	530.7251	1043.4164	522.2118	1042.4324	521.7198	V	1187.5480	594.2776	1170.5215	585.7644	1169.5374	585.2724	9
11	1197.5018	599.2546	1180.4753	590.7413	1179.4913	590.2493	H	1088.4796	544.7434	1071.4530	536.2302	1070.4690	535.7381	8
12	1344.5703	672.7888	1327.5437	664.2755	1326.5597	663.7835	F	951.4207	476.2140	934.3941	467.7007	933.4101	467.2087	7
13	1473.6128	737.3101	1456.5863	728.7968	1455.6023	728.3048	E	804.3523	402.6798	787.3257	394.1665	786.3417	393.6745	6
14	1602.6554	801.8314	1585.6289	793.3181	1584.6449	792.8261	E	675.3097	338.1585	658.2831	329.6452	657.2991	329.1532	5
15	1717.6824	859.3448	1700.6558	850.8316	1699.6718	850.3395	D	546.2671	273.6372	529.2405	265.1239	528.2565	264.6319	4
16	1864.7508	932.8790	1847.7242	924.3658	1846.7402	923.8738	F	431.2401	216.1237	414.2136	207.6104			3
17	2001.8097	1001.4085	1984.7832	992.8952	1983.7991	992.4032	H	284.1717	142.5895	267.1452	134.0762			2
18							K	147.1128	74.0600	130.0863	65.5468			1

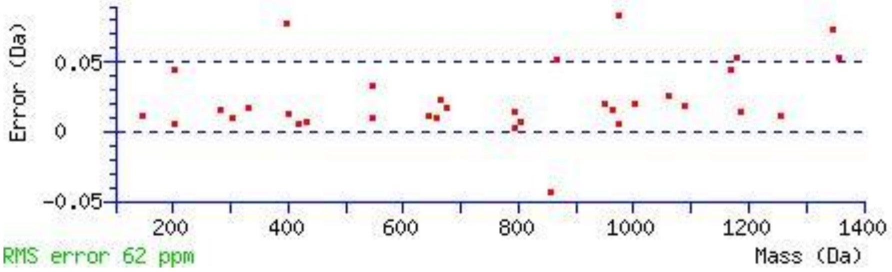

17_MS/MS Fragmentation of **SSPFLDAAPSLPDSR**

Found in **O95180**, Voltage-dependent T-type calcium channel subunit alpha-1H OS=Homo sapiens GN=CACNA1H PE=1 SV=4

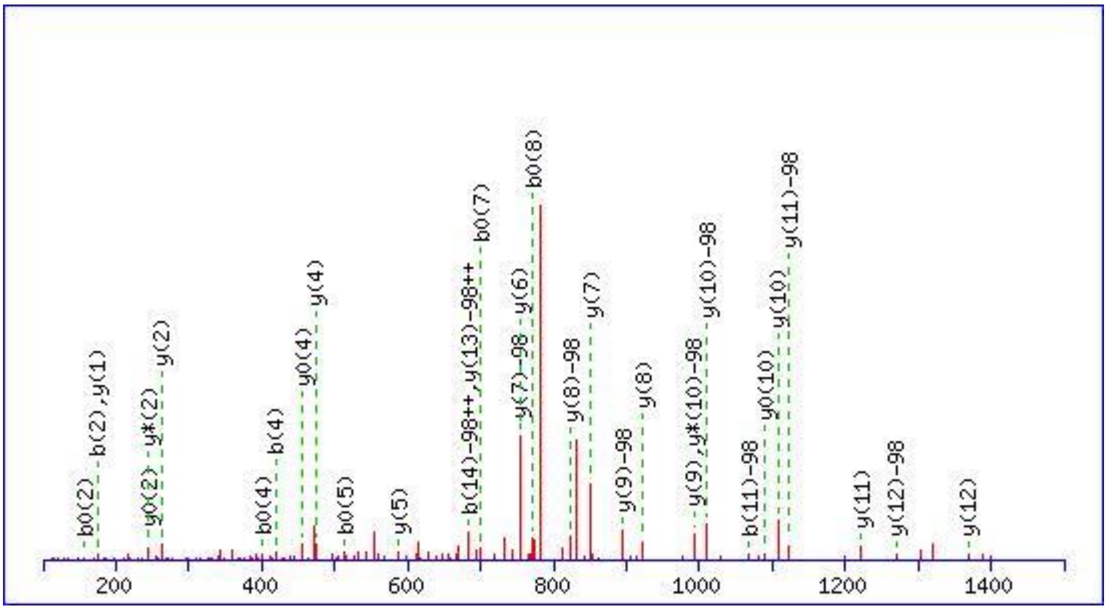

Monoisotopic mass of neutral peptide Mr(calc): 1638.7290

Variable modifications:

S10 : Phospho (ST), with neutral losses 0.0000(shown in table), 97.9769

Ions Score: 74 **Expect:** 9.5e-005

Matches : 32/212 fragment ions using 49 most intense peaks (help)

#	b	b^{++}	b^0	b^{0++}	Seq.	y	y^{++}	y*	y^{*++}	y^0	y^{0++}	#
1	88.0393	44.5233	70.0287	35.5180	S							15
2	**175.0713**	88.0393	157.0608	79.0340	S	1552.7043	776.8558	1535.6778	768.3425	1534.6937	767.8505	14
3	272.1241	136.5657	254.1135	127.5604	P	1465.6723	733.3398	1448.6457	724.8265	1447.6617	724.3345	13
4	**419.1925**	210.0999	401.1819	201.0946	F	***1368.6195***	684.8134	1351.5930	676.3001	1350.6090	675.8081	12
5	532.2766	266.6419	514.2660	257.6366	L	***1221.5511***	611.2792	1204.5246	602.7659	1203.5405	602.2739	11
6	647.3035	324.1554	629.2930	315.1501	D	***1108.4670***	554.7372	1091.4405	546.2239	1090.4565	545.7319	10
7	718.3406	359.6740	700.3301	350.6687	A	***993.4401***	497.2237	976.4136	488.7104	975.4295	488.2184	9
8	789.3777	395.1925	771.3672	386.1872	A	***922.4030***	461.7051	905.3764	453.1919	904.3924	452.6998	8
9	886.4305	443.7189	868.4199	434.7136	P	***851.3659***	426.1866	834.3393	417.6733	833.3553	417.1813	7
10	1053.4289	527.2181	1035.4183	518.2128	S	***754.3131***	377.6602	737.2866	369.1469	736.3025	368.6549	6
11	1166.5129	583.7601	1148.5024	574.7548	L	***587.3148***	294.1610	570.2882	285.6477	569.3042	285.1557	5
12	1263.5657	632.2865	1245.5551	623.2812	P	***474.2307***	237.6190	457.2041	229.1057	456.2201	228.6137	4
13	1378.5926	689.8000	1360.5821	680.7947	D	377.1779	189.0926	360.1514	180.5793	359.1674	180.0873	3
14	1465.6247	733.3160	1447.6141	724.3107	S	***262.1510***	131.5791	245.1244	123.0659	244.1404	122.5738	2
15					R	***175.1190***	88.0631	158.0924	79.5498			1

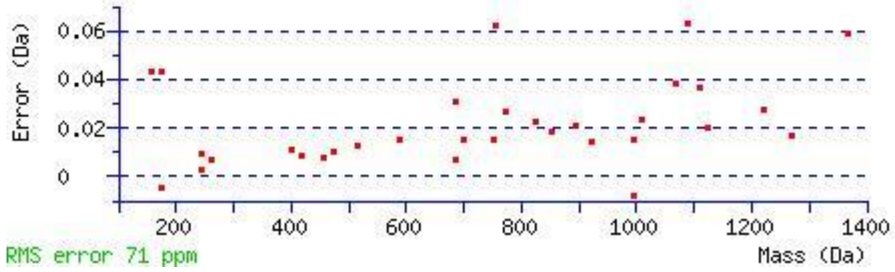
Error (Da)
0.06
0.04
0.02
0
200
400
600
800
1000
1200
1400
Mass (Da)
RMS error 71 ppm

18_MS/MS Fragmentation of **SSPFLDAAPSLPDSR**

Found in **O95180**, Voltage-dependent T-type calcium channel subunit alpha-1H OS=Homo sapiens GN=CACNA1H PE=1 SV=4

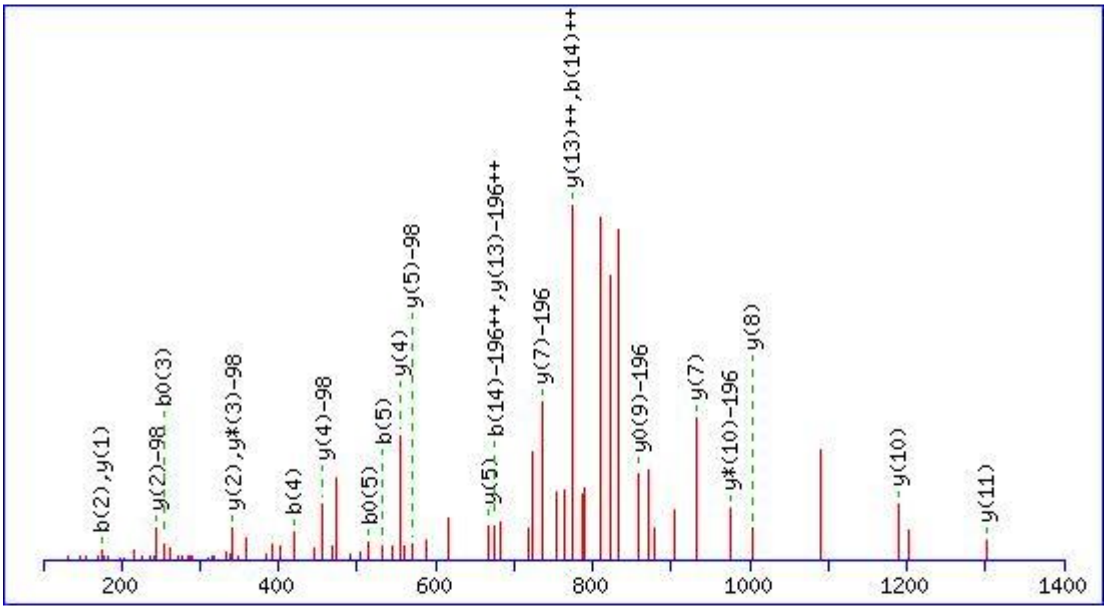

Monoisotopic mass of neutral peptide Mr(calc): 1718.6954

Variable modifications:

S10 : Phospho (ST), with neutral losses 0.0000(shown in table), 97.9769

S14 : Phospho (ST), with neutral losses 0.0000(shown in table), 97.9769

Ions Score: 36 Expect: 0.54

Matches : 24/236 fragment ions using 38 most intense peaks (help)

#	b	b^{++}	b^{0}	b^{0++}	Seq.	y	y^{++}	y*	y^{*++}	y^{0}	y^{0++}	#
1	88.0393	44.5233	70.0287	35.5180	S							15
2	175.0713	88.0393	157.0608	79.0340	S	1632.6706	816.8390	1615.6441	808.3257	1614.6601	807.8337	14
3	272.1241	136.5657	254.1135	127.5604	P	1545.6386	773.3229	1528.6121	764.8097	1527.6281	764.3177	13
4	419.1925	210.0999	401.1819	201.0946	F	1448.5859	724.7966	1431.5593	716.2833	1430.5753	715.7913	12
5	532.2766	266.6419	514.2660	257.6366	L	1301.5174	651.2624	1284.4909	642.7491	1283.5069	642.2571	11
6	647.3035	324.1554	629.2930	315.1501	D	1188.4334	594.7203	1171.4068	586.2071	1170.4228	585.7150	10
7	718.3406	359.6740	700.3301	350.6687	A	1073.4064	537.2069	1056.3799	528.6936	1055.3959	528.2016	9
8	789.3777	395.1925	771.3672	386.1872	A	1002.3693	501.6883	985.3428	493.1750	984.3588	492.6830	8
9	886.4305	443.7189	868.4199	434.7136	P	931.3322	466.1697	914.3057	457.6565	913.3216	457.1645	7
10	1053.4289	527.2181	1035.4183	518.2128	S	834.2794	417.6434	817.2529	409.1301	816.2689	408.6381	6
11	1166.5129	583.7601	1148.5024	574.7548	L	667.2811	334.1442	650.2545	325.6309	649.2705	325.1389	5
12	1263.5657	632.2865	1245.5551	623.2812	P	554.1970	277.6021	537.1705	269.0889	536.1865	268.5969	4
13	1378.5926	689.8000	1360.5821	680.7947	D	457.1443	229.0758	440.1177	220.5625	439.1337	220.0705	3
14	1545.5910	773.2991	1527.5804	764.2939	S	342.1173	171.5623	325.0908	163.0490	324.1067	162.5570	2
15					R	175.1190	88.0631	158.0924	79.5498			1

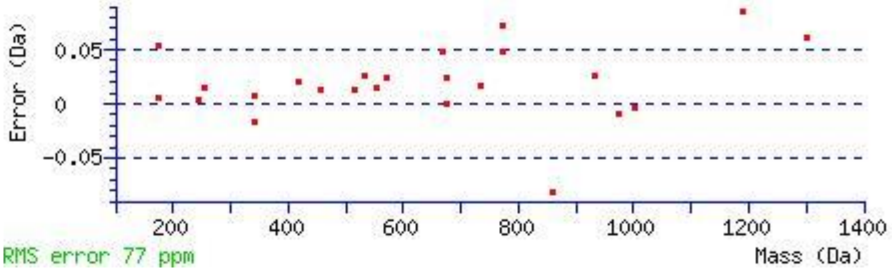

Error (Da)
0.05
0
-0.05
200
400
600
800
1000
1200
1400
Mass (Da)
RMS error 77 ppm

19_MS/MS Fragmentation of **SSPFLDAAPSLPDSRR**

Found in **O95180**, Voltage-dependent T-type calcium channel subunit alpha-1H OS=Homo sapiens GN=CACNA1H PE=1 SV=4

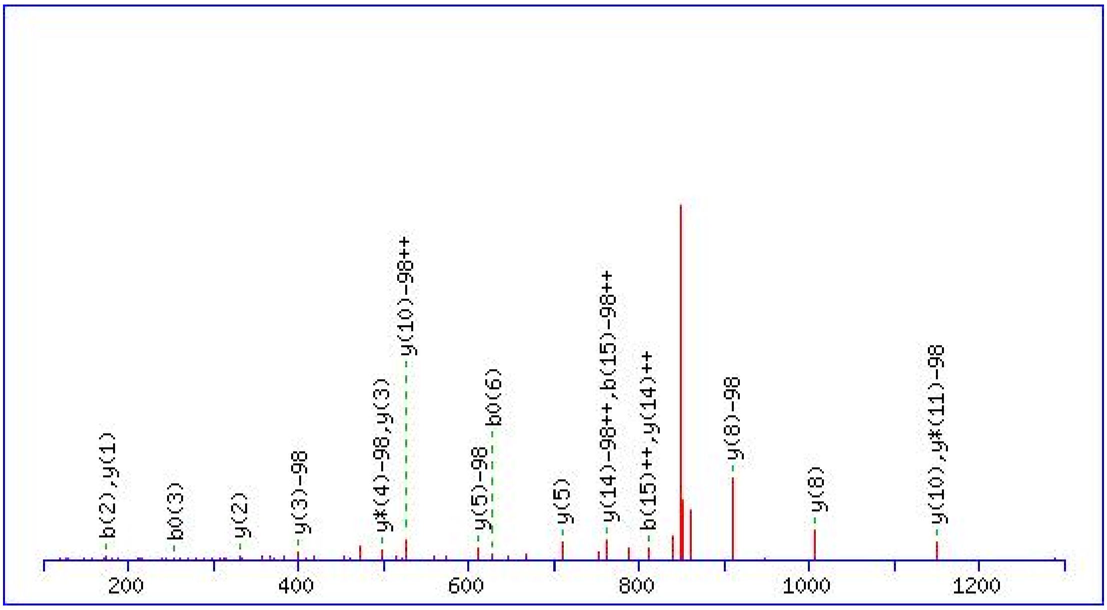

Monoisotopic mass of neutral peptide Mr(calc): 1794.8302

Variable modifications:

S14 : Phospho (ST), with neutral losses 0.0000(shown in table), 97.9769

Ions Score: 41 Expect: 0.2

Matches : 19/236 fragment ions using 20 most intense peaks (help)

#	b	b^{++}	b*	b^{*++}	b^0	b^{0++}	Seq.	y	y^{++}	y*	y^{*++}	y^0	y^{0++}	#
1	88.0393	44.5233			70.0287	35.5180	S							16
2	**175.0713**	88.0393			157.0608	79.0340	S	1708.8054	854.9064	1691.7789	846.3931	1690.7949	845.9011	15
3	272.1241	136.5657			254.1135	127.5604	P	1621.7734	811.3903	1604.7468	802.8771	1603.7628	802.3851	14
4	419.1925	210.0999			401.1819	201.0946	F	1524.7206	762.8640	1507.6941	754.3507	1506.7101	753.8587	13
5	532.2766	266.6419			514.2660	257.6366	L	1377.6522	689.3297	1360.6257	680.8165	1359.6417	680.3245	12
6	647.3035	324.1554			629.2930	315.1501	D	1264.5682	632.7877	1247.5416	624.2744	1246.5576	623.7824	11
7	718.3406	359.6740			700.3301	350.6687	A	***1149.5412***	575.2742	1132.5147	566.7610	1131.5306	566.2690	10
8	789.3777	395.1925			771.3672	386.1872	A	1078.5041	539.7557	1061.4776	531.2424	1060.4935	530.7504	9
9	886.4305	443.7189			868.4199	434.7136	P	***1007.4670***	504.2371	990.4404	495.7239	989.4564	495.2318	8

**10**	973.4625	487.2349			955.4520	478.2296	**S**	910.4142	455.7107	893.3877	447.1975	892.4037	446.7055	**7**
**11**	1086.5466	543.7769			1068.5360	534.7717	**L**	823.3822	412.1947	806.3556	403.6815	805.3716	403.1895	**6**
**12**	1183.5994	592.3033			1165.5888	583.2980	**P**	***710.2981***	355.6527	693.2716	347.1394	692.2876	346.6474	**5**
**13**	1298.6263	649.8168			1280.6157	640.8115	**D**	613.2454	307.1263	596.2188	298.6130	595.2348	298.1210	**4**
**14**	1465.6247	733.3160			1447.6141	724.3107	**S**	***498.2184***	249.6128	481.1919	241.0996	480.2079	240.6076	**3**
**15**	1621.7258	**811.3665**	1604.6992	802.8533	1603.7152	802.3612	**R**	***331.2201***	166.1137	314.1935	157.6004			**2**
**16**							**R**	***175.1190***	88.0631	158.0924	79.5498			**1**

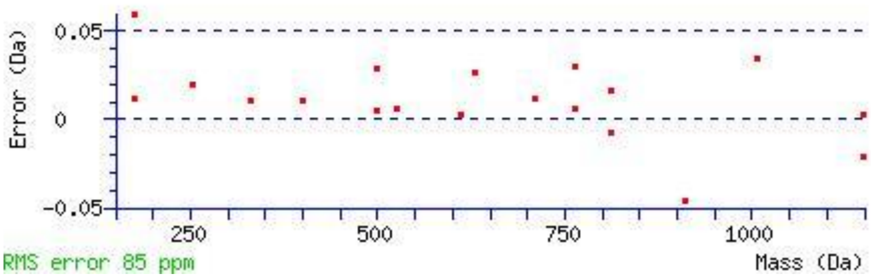

20_MS/MS Fragmentation of **RGSSSSGDPPLGDQKPPASLR**

Found in **O95180**, Voltage-dependent T-type calcium channel subunit alpha-1H OS=Homo sapiens GN=CACNA1H PE=1 SV=4

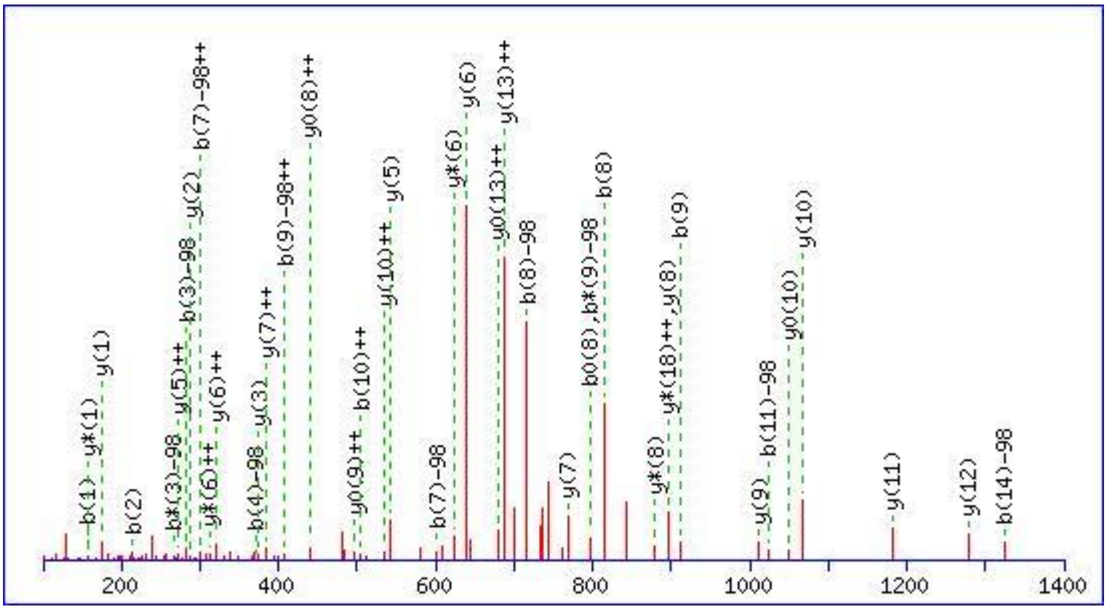

Monoisotopic mass of neutral peptide Mr(calc): 2188.0274

Variable modifications:

S3 : Phospho (ST), with neutral losses 97.9769(shown in table), 0.0000

Ions Score: 77 Expect: 6.6e-005

Matches : 41/352 fragment ions using 64 most intense peaks (help)

#	b	b^{++}	b*	b^{*++}	b^0	b^{0++}	Seq.	y	y^{++}	y*	y^{*++}	y^0	y^{0++}	#
1	***157.1084***	79.0578	140.0818	70.5446			R							21
2	***214.1299***	107.5686	197.1033	99.0553			G	1934.9566	967.9820	1917.9301	959.4687	1916.9461	958.9767	20
3	***283.1513***	142.0793	266.1248	133.5660	265.1407	133.0740	S	1877.9352	939.4712	1860.9086	930.9580	1859.9246	930.4659	19
4	***370.1833***	185.5953	353.1568	177.0820	352.1728	176.5900	S	1808.9137	904.9605	1791.8872	896.4472	1790.9032	895.9552	18
5	457.2154	229.1113	440.1888	220.5980	439.2048	220.1060	S	1721.8817	861.4445	1704.8551	852.9312	1703.8711	852.4392	17
6	544.2474	272.6273	527.2208	264.1141	526.2368	263.6221	S	1634.8497	817.9285	1617.8231	809.4152	1616.8391	808.9232	16
7	***601.2689***	**301.1381**	584.2423	292.6248	583.2583	292.1328	G	1547.8176	774.4125	1530.7911	765.8992	1529.8071	765.4072	15
8	***716.2958***	358.6515	699.2693	350.1383	698.2852	349.6463	D	1490.7962	745.9017	1473.7696	737.3884	1472.7856	736.8964	14
9	813.3486	**407.1779**	796.3220	398.6646	795.3380	398.1726	P	1375.7692	688.3883	1358.7427	679.8750	1357.7587	679.3830	13

**10**	910.4013	455.7043	893.3748	447.1910	892.3908	446.6990	**P**	***1278.7165***	639.8619	1261.6899	631.3486	1260.7059	630.8566	**12**
**11**	***1023.4854***	512.2463	1006.4588	503.7331	1005.4748	503.2411	**L**	***1181.6637***	591.3355	1164.6371	582.8222	1163.6531	582.3302	**11**
**12**	1080.5069	540.7571	1063.4803	532.2438	1062.4963	531.7518	**G**	***1068.5796***	534.7935	1051.5531	526.2802	1050.5691	525.7882	**10**
**13**	1195.5338	598.2705	1178.5073	589.7573	1177.5232	589.2653	**D**	***1011.5582***	506.2827	994.5316	497.7694	993.5476	497.2774	**9**
**14**	***1323.5924***	662.2998	1306.5658	653.7866	1305.5818	653.2945	**Q**	***896.5312***	448.7693	879.5047	440.2560	878.5207	439.7640	**8**
**15**	1451.6873	726.3473	1434.6608	717.8340	1433.6768	717.3420	**K**	***768.4726***	384.7400	751.4461	376.2267	750.4621	375.7347	**7**
**16**	1548.7401	774.8737	1531.7136	766.3604	1530.7295	765.8684	**P**	***640.3777***	320.6925	623.3511	312.1792	622.3671	311.6872	**6**
**17**	1645.7929	823.4001	1628.7663	814.8868	1627.7823	814.3948	**P**	***543.3249***	272.1661	526.2984	263.6528	525.3144	263.1608	**5**
**18**	1716.8300	858.9186	1699.8034	850.4054	1698.8194	849.9133	**A**	446.2722	223.6397	429.2456	215.1264	428.2616	214.6344	**4**
**19**	1803.8620	902.4346	1786.8355	893.9214	1785.8515	893.4294	**S**	***375.2350***	188.1212	358.2085	179.6079	357.2245	179.1159	**3**
**20**	1916.9461	958.9767	1899.9195	950.4634	1898.9355	949.9714	**L**	***288.2030***	144.6051	271.1765	136.0919			**2**
**21**							**R**	***175.1190***	88.0631	158.0924	79.5498			**1**

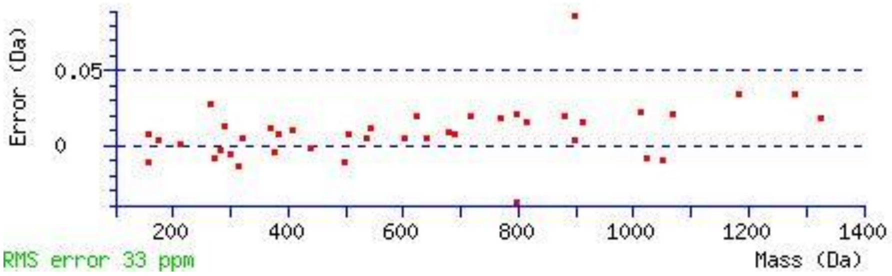

21_MS/MS Fragmentation of **RGSSSSGDPPLGDQKPPASLR**

Found in **O95180**, Voltage-dependent T-type calcium channel subunit alpha-1H OS=Homo sapiens GN=CACNA1H PE=1 SV=4

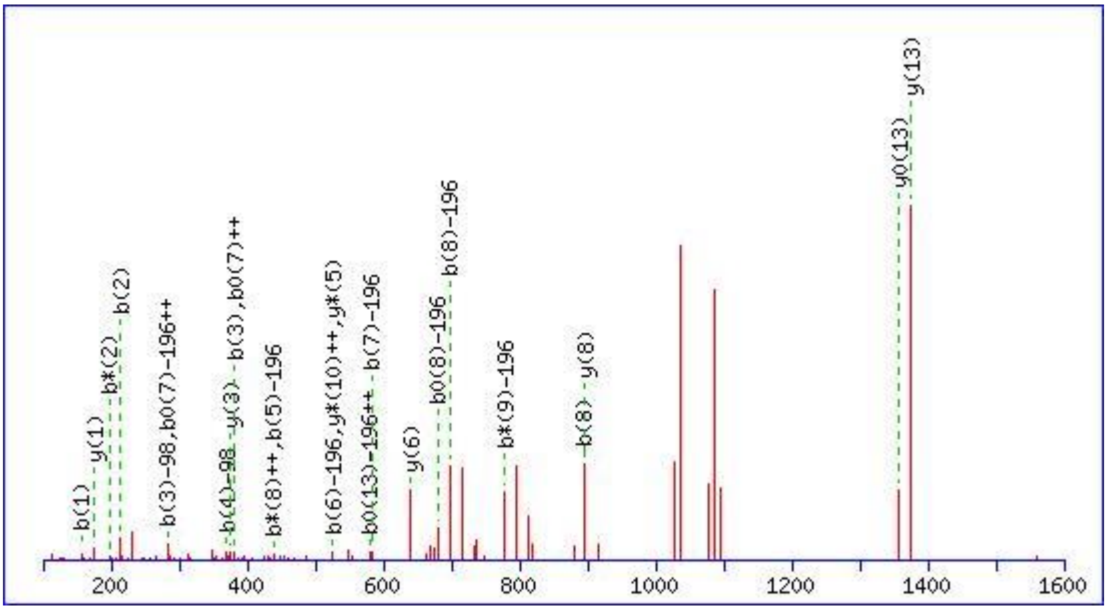

Monoisotopic mass of neutral peptide Mr(calc): 2267.9937

Variable modifications:

S3 : Phospho (ST), with neutral losses 97.9769(shown in table), 0.0000

S5 : Phospho (ST), with neutral losses 97.9769(shown in table), 0.0000

Ions Score: 43 Expect: 0.16

Matches : 25/364 fragment ions using 40 most intense peaks (help)

#	b	b^{++}	b*	b^{*++}	b^{0}	b^{0++}	Seq.	y	y^{++}	y*	y^{*++}	y^{0}	y^{0++}	#
1	***157.1084***	79.0578	140.0818	70.5446			R							21
2	***214.1299***	107.5686	197.1033	99.0553			G	1916.9461	958.9767	1899.9195	950.4634	1898.9355	949.9714	20
3	***283.1513***	142.0793	266.1248	133.5660	265.1407	133.0740	S	1859.9246	930.4659	1842.8981	921.9527	1841.9140	921.4607	19
4	***370.1833***	185.5953	353.1568	177.0820	352.1728	176.5900	S	1790.9032	895.9552	1773.8766	887.4419	1772.8926	886.9499	18
5	***439.2048***	220.1060	422.1783	211.5928	421.1942	211.1008	S	1703.8711	852.4392	1686.8446	843.9259	1685.8606	843.4339	17
6	***526.2368***	263.6221	509.2103	255.1088	508.2263	254.6168	S	1634.8497	817.9285	1617.8231	809.4152	1616.8391	808.9232	16
7	***583.2583***	292.1328	566.2317	283.6195	565.2477	283.1275	G	1547.8176	774.4125	1530.7911	765.8992	1529.8071	765.4072	15
8	***698.2852***	349.6463	681.2587	341.1330	680.2747	340.6410	D	1490.7962	745.9017	1473.7696	737.3884	1472.7856	736.8964	14
9	795.3380	398.1726	778.3115	389.6594	777.3274	389.1674	P	***1375.7692***	688.3883	1358.7427	679.8750	1357.7587	679.3830	13

10	892.3908	446.6990	875.3642	438.1857	874.3802	437.6937	P	1278.7165	639.8619	1261.6899	631.3486	1260.7059	630.8566	12
11	1005.4748	503.2411	988.4483	494.7278	987.4643	494.2358	L	1181.6637	591.3355	1164.6371	582.8222	1163.6531	582.3302	11
12	1062.4963	531.7518	1045.4697	523.2385	1044.4857	522.7465	G	1068.5796	534.7935	1051.5531	526.2802	1050.5691	525.7882	10
13	1177.5232	589.2653	1160.4967	580.7520	1159.5127	580.2600	D	1011.5582	506.2827	994.5316	497.7694	993.5476	497.2774	9
14	1305.5818	653.2945	1288.5553	644.7813	1287.5713	644.2893	Q	***896.5312***	448.7693	879.5047	440.2560	878.5207	439.7640	8
15	1433.6768	717.3420	1416.6502	708.8288	1415.6662	708.3367	K	768.4726	384.7400	751.4461	376.2267	750.4621	375.7347	7
16	1530.7295	765.8684	1513.7030	757.3551	1512.7190	756.8631	P	***640.3777***	320.6925	623.3511	312.1792	622.3671	311.6872	6
17	1627.7823	814.3948	1610.7558	805.8815	1609.7717	805.3895	P	543.3249	272.1661	526.2984	263.6528	525.3144	263.1608	5
18	1698.8194	849.9133	1681.7929	841.4001	1680.8089	840.9081	A	446.2722	223.6397	429.2456	215.1264	428.2616	214.6344	4
19	1785.8514	893.4294	1768.8249	884.9161	1767.8409	884.4241	S	***375.2350***	188.1212	358.2085	179.6079	357.2245	179.1159	3
20	1898.9355	949.9714	1881.9090	941.4581	1880.9249	940.9661	L	288.2030	144.6051	271.1765	136.0919			2
21							R	***175.1190***	88.0631	158.0924	79.5498			1

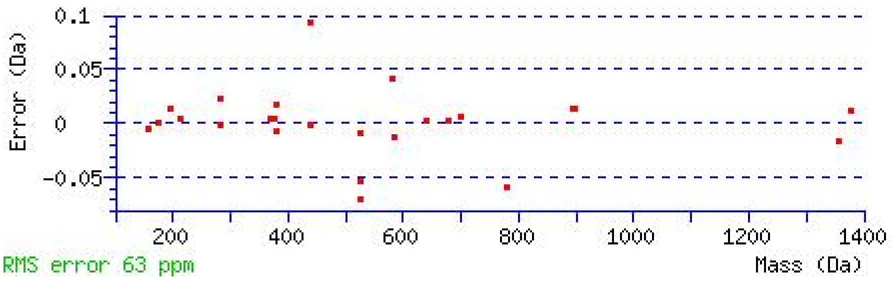

22_MS/MS Fragmentation of **SSWSSLGR**

Found in **O95180**, Voltage-dependent T-type calcium channel subunit alpha-1H OS=Homo sapiens GN=CACNA1H PE=1 SV=4

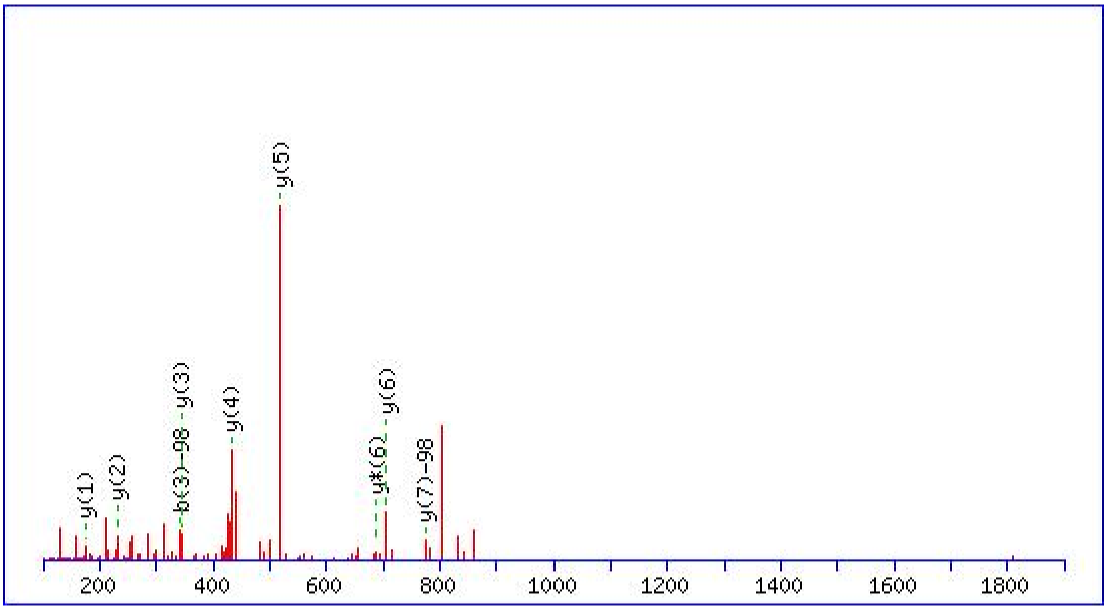

Monoisotopic mass of neutral peptide Mr(calc): 958.3909

Variable modifications:

S2 : Phospho (ST), with neutral losses 97.9769(shown in table), 0.0000

Ions Score: 43 Expect: 0.074

Matches : 9/94 fragment ions using 24 most intense peaks (help)

#	b	b^{++}	b^{0}	b^{0++}	Seq.	y	y^{++}	y*	y^{*++}	y^{0}	y^{0++}	#
1	88.0393	44.5233	70.0287	35.5180	S							8
2	157.0608	79.0340	139.0502	70.0287	S	***774.3893***	387.6983	757.3628	379.1850	756.3787	378.6930	7
3	**343.1401**	172.0737	325.1295	163.0684	W	***705.3678***	353.1876	688.3413	344.6743	687.3573	344.1823	6
4	430.1721	215.5897	412.1615	206.5844	S	***519.2885***	260.1479	502.2620	251.6346	501.2780	251.1426	5
5	517.2041	259.1057	499.1936	250.1004	S	***432.2565***	216.6319	415.2300	208.1186	414.2459	207.6266	4
6	630.2882	315.6477	612.2776	306.6425	L	***345.2245***	173.1159	328.1979	164.6026			3
7	687.3097	344.1585	669.2991	335.1532	G	***232.1404***	116.5738	215.1139	108.0606			2
8					R	***175.1190***	88.0631	158.0924	79.5498			1

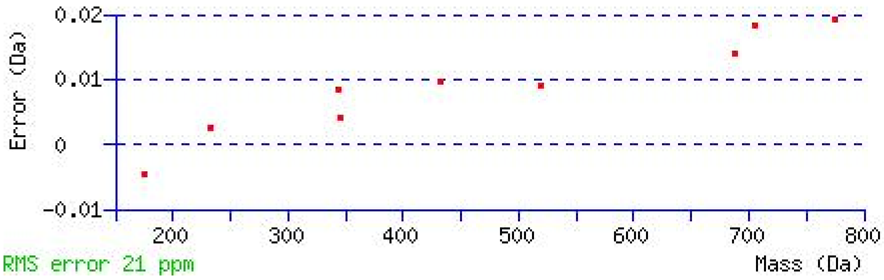

23_MS/MS Fragmentation of **ESLLSGEGKGSTDDEAEDGR**

Found in **O95180**, Voltage-dependent T-type calcium channel subunit alpha-1H OS=Homo sapiens GN=CACNA1H PE=1 SV=4

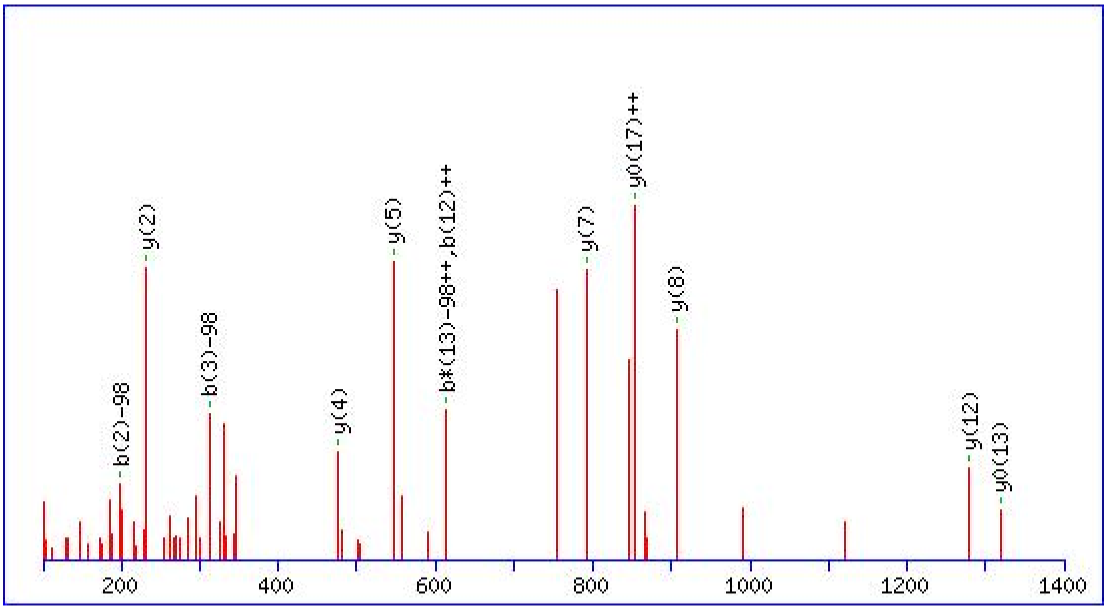

Monoisotopic mass of neutral peptide Mr(calc): 2130.8590

Variable modifications:

S2 : Phospho (ST), with neutral losses 97.9769(shown in table), 0.0000

Ions Score: 49 Expect: 0.027

Matches : 12/308 fragment ions using 12 most intense peaks (help)

#	b	b^{++}	b*	b^{*++}	b^{0}	b^{0++}	Seq.	y	y^{++}	y*	y^{*++}	y^{0}	y^{0++}	#
1	130.0499	65.5286			112.0393	56.5233	E							20
2	**199.0713**	100.0393			181.0608	91.0340	S	1904.8468	952.9270	1887.8203	944.4138	1886.8363	943.9218	19
3	**312.1554**	156.5813			294.1448	147.5761	L	1835.8254	918.4163	1818.7988	909.9030	1817.8148	909.4110	18
4	425.2395	213.1234			407.2289	204.1181	L	1722.7413	861.8743	1705.7147	853.3610	1704.7307	852.8690	17
5	512.2715	256.6394			494.2609	247.6341	S	1609.6572	805.3323	1592.6307	796.8190	1591.6467	796.3270	16
6	569.2930	285.1501			551.2824	276.1448	G	1522.6252	761.8162	1505.5987	753.3030	1504.6146	752.8110	15
7	698.3355	349.6714			680.3250	340.6661	E	1465.6037	733.3055	1448.5772	724.7922	1447.5932	724.3002	14
8	755.3570	378.1821			737.3464	369.1769	G	1336.5611	668.7842	1319.5346	660.2709	1318.5506	659.7789	13
9	883.4520	442.2296	866.4254	433.7163	865.4414	433.2243	K	*1279.5397*	640.2735	1262.5131	631.7602	1261.5291	631.2682	12

10	940.4734	470.7404	923.4469	462.2271	922.4629	461.7351	G	1151.4447	576.2260	1134.4182	567.7127	1133.4342	567.2207	11
11	1027.5055	514.2564	1010.4789	505.7431	1009.4949	505.2511	S	1094.4233	547.7153	1077.3967	539.2020	1076.4127	538.7100	10
12	1128.5531	564.7802	1111.5266	556.2669	1110.5426	555.7749	T	1007.3912	504.1993	990.3647	495.6860	989.3807	495.1940	9
13	1243.5801	622.2937	1226.5535	613.7804	1225.5695	613.2884	D	906.3435	453.6754	889.3170	445.1621	888.3330	444.6701	8
14	1358.6070	679.8072	1341.5805	671.2939	1340.5965	670.8019	D	791.3166	396.1619	774.2901	387.6487	773.3060	387.1567	7
15	1487.6496	744.3284	1470.6231	735.8152	1469.6391	735.3232	E	676.2897	338.6485	659.2631	330.1352	658.2791	329.6432	6
16	1558.6867	779.8470	1541.6602	771.3337	1540.6762	770.8417	A	547.2471	274.1272	530.2205	265.6139	529.2365	265.1219	5
17	1687.7293	844.3683	1670.7028	835.8550	1669.7188	835.3630	E	476.2100	238.6086	459.1834	230.0953	458.1994	229.6033	4
18	1802.7563	901.8818	1785.7297	893.3685	1784.7457	892.8765	D	347.1674	174.0873	330.1408	165.5740	329.1568	165.0820	3
19	1859.7777	930.3925	1842.7512	921.8792	1841.7672	921.3872	G	232.1404	116.5738	215.1139	108.0606			2
20							R	175.1190	88.0631	158.0924	79.5498			1

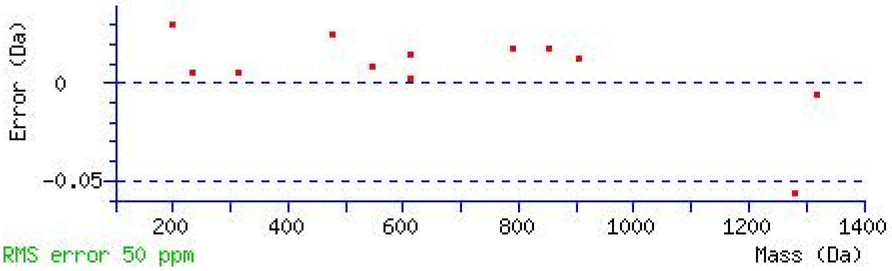

24_MS/MS Fragmentation of **ESLLSGEGK**

Found in **O95180**, Voltage-dependent T-type calcium channel subunit alpha-1H OS=Homo sapiens GN=CACNA1H PE=1 SV=4

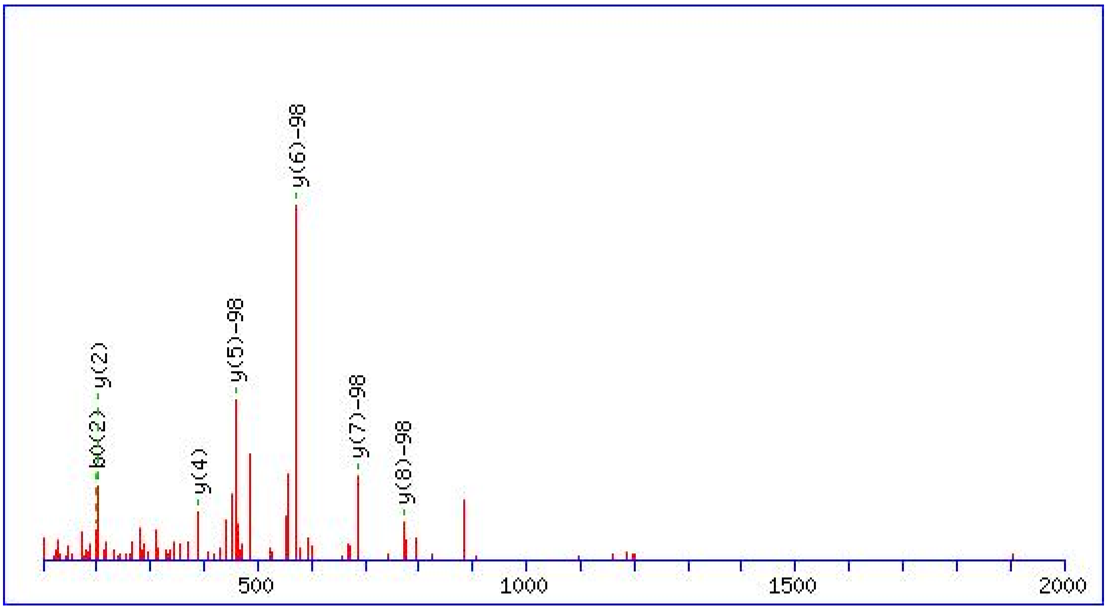

Monoisotopic mass of neutral peptide Mr(calc): 998.4321

Variable modifications:

S5 : Phospho (ST), with neutral losses 97.9769(shown in table), 0.0000

Ions Score: 55 Expect: 0.0051

Matches : 7/116 fragment ions using 9 most intense peaks (help)

#	b	b^{++}	b^{0}	b^{0++}	Seq.	y	y^{++}	y*	y^{*++}	y^{0}	y^{0++}	#
1	130.0499	65.5286	112.0393	56.5233	E							9
2	217.0819	109.0446	199.0713	100.0393	S	***772.4199***	386.7136	755.3934	378.2003	754.4094	377.7083	8
3	330.1660	165.5866	312.1554	156.5813	L	***685.3879***	343.1976	668.3614	334.6843	667.3774	334.1923	7
4	443.2500	222.1287	425.2395	213.1234	L	***572.3039***	286.6556	555.2773	278.1423	554.2933	277.6503	6
5	512.2715	256.6394	494.2609	247.6341	S	***459.2198***	230.1135	442.1932	221.6003	441.2092	221.1082	5
6	569.2930	285.1501	551.2824	276.1448	G	***390.1983***	195.6028	373.1718	187.0895	372.1878	186.5975	4
7	698.3355	349.6714	680.3250	340.6661	E	333.1769	167.0921	316.1503	158.5788	315.1663	158.0868	3
8	755.3570	378.1821	737.3464	369.1769	G	***204.1343***	102.5708	187.1077	94.0575			2
9					K	147.1128	74.0600	130.0863	65.5468			1

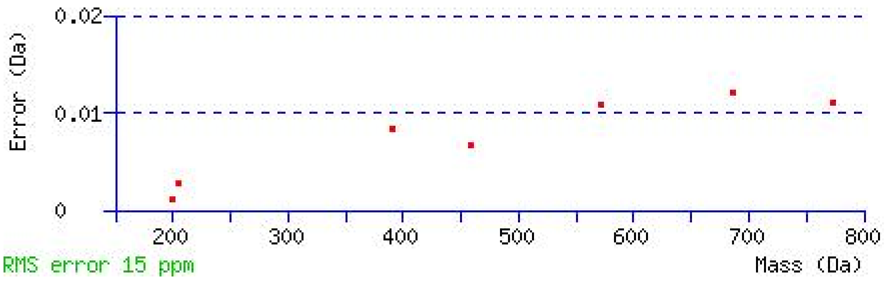

25_MS/MS Fragmentation of **ESLLSGEGKGSTDDEAEDGR**

Found in **O95180**, Voltage-dependent T-type calcium channel subunit alpha-1H OS=Homo sapiens GN=CACNA1H PE=1 SV=4

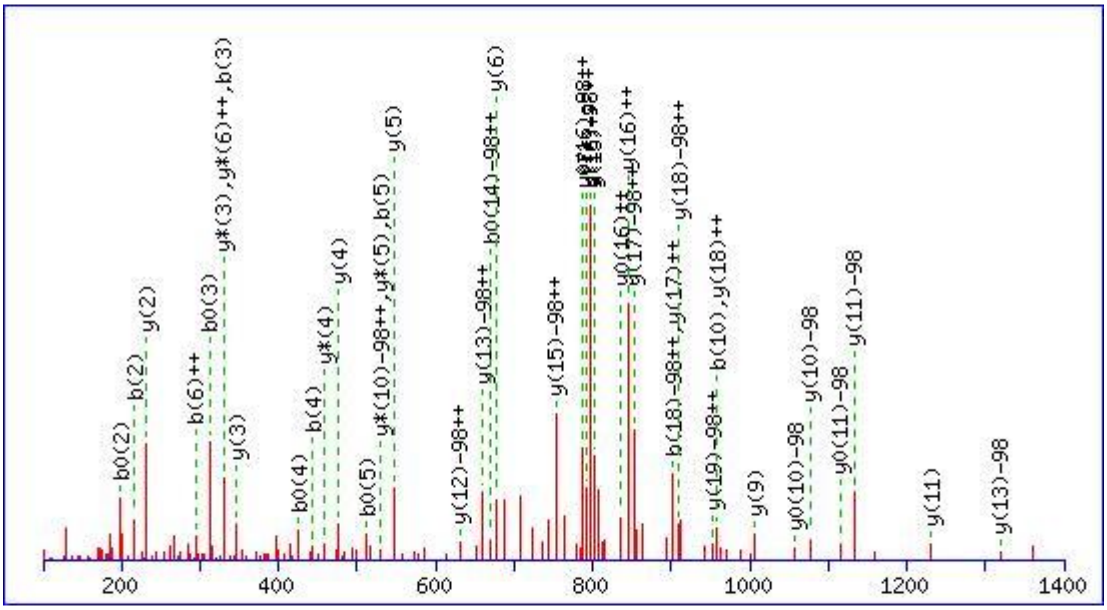

Monoisotopic mass of neutral peptide Mr(calc): 2130.8590

Variable modifications:

S11 : Phospho (ST), with neutral losses 97.9769(shown in table), 0.0000

Ions Score: 75 Expect: 6.8e-005

Matches : 43/322 fragment ions using 54 most intense peaks (help)

#	b	b^{++}	b*	b^{*++}	b^0	b^{0++}	Seq.	y	y^{++}	y*	y^{*++}	y^0	y^{0++}	#
1	130.0499	65.5286			112.0393	56.5233	E							20
2	**217.0819**	109.0446			199.0713	100.0393	S	1904.8468	952.9270	1887.8203	944.4138	1886.8363	943.9218	19
3	**330.1660**	165.5866			312.1554	156.5813	L	1817.8148	909.4110	1800.7882	900.8978	1799.8042	900.4058	18
4	**443.2500**	222.1287			425.2395	213.1234	L	1704.7307	852.8690	1687.7042	844.3557	1686.7202	843.8637	17
5	**530.2821**	265.6447			512.2715	256.6394	S	1591.6467	796.3270	1574.6201	787.8137	1573.6361	787.3217	16
6	587.3035	**294.1554**			569.2930	285.1501	G	1504.6146	752.8110	1487.5881	744.2977	1486.6041	743.8057	15
7	716.3461	358.6767			698.3355	349.6714	E	1447.5932	724.3002	1430.5666	715.7869	1429.5826	715.2949	14
8	773.3676	387.1874			755.3570	378.1821	G	***1318.5506***	659.7789	1301.5240	651.2657	1300.5400	650.7736	13
9	901.4625	451.2349	884.4360	442.7216	883.4520	442.2296	K	1261.5291	631.2682	1244.5026	622.7549	1243.5185	622.2629	12

10	**958.4840**	479.7456	941.4575	471.2324	940.4734	470.7404	G	***1133.4342***	567.2207	1116.4076	558.7074	1115.4236	558.2154	11
11	1027.5055	514.2564	1010.4789	505.7431	1009.4949	505.2511	S	***1076.4127***	538.7100	1059.3861	530.1967	1058.4021	529.7047	10
12	1128.5531	564.7802	1111.5266	556.2669	1110.5426	555.7749	T	***1007.3912***	504.1993	990.3647	495.6860	989.3807	495.1940	9
13	1243.5801	622.2937	1226.5535	613.7804	1225.5695	613.2884	D	906.3435	453.6754	889.3170	445.1621	888.3330	444.6701	8
14	1358.6070	679.8072	1341.5805	671.2939	1340.5965	670.8019	D	***791.3166***	396.1619	774.2901	387.6487	773.3060	387.1567	7
15	1487.6496	744.3284	1470.6231	735.8152	1469.6391	735.3232	E	***676.2897***	338.6485	659.2631	330.1352	658.2791	329.6432	6
16	1558.6867	779.8470	1541.6602	771.3337	1540.6762	770.8417	A	***547.2471***	274.1272	530.2205	265.6139	529.2365	265.1219	5
17	1687.7293	844.3683	1670.7028	835.8550	1669.7188	835.3630	E	***476.2100***	238.6086	459.1834	230.0953	458.1994	229.6033	4
18	1802.7563	**901.8818**	1785.7297	893.3685	1784.7457	892.8765	D	***347.1674***	174.0873	330.1408	165.5740	329.1568	165.0820	3
19	1859.7777	930.3925	1842.7512	921.8792	1841.7672	921.3872	G	***232.1404***	116.5738	215.1139	108.0606			2
20							R	175.1190	88.0631	158.0924	79.5498			1

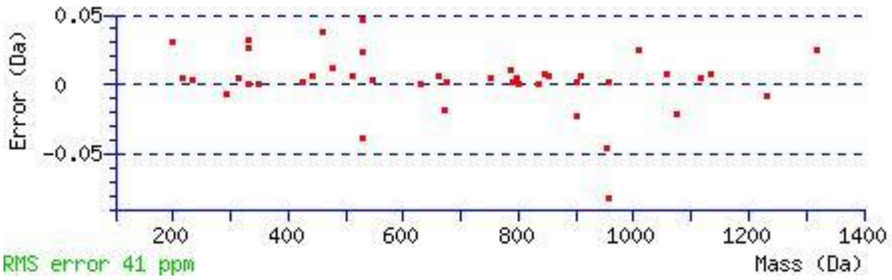

26_MS/MS Fragmentation of **GSTDDEAEDGR**

Found in **O95180**, Voltage-dependent T-type calcium channel subunit alpha-1H OS=Homo sapiens GN=CACNA1H PE=1 SV=4

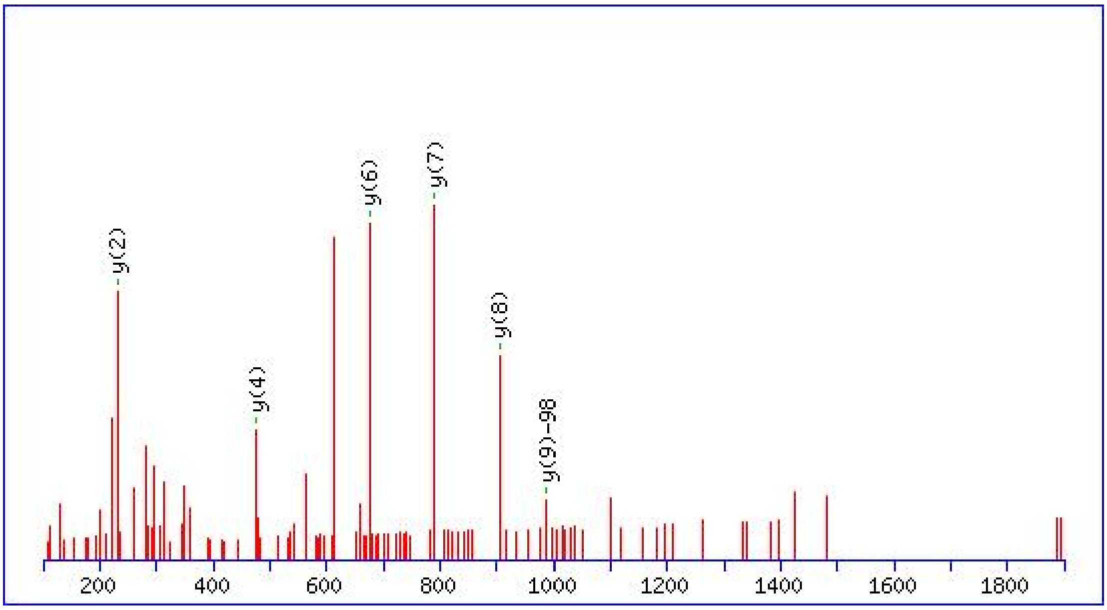

Monoisotopic mass of neutral peptide Mr(calc): 1230.4038

Variable modifications:

T3 : Phospho (ST), with neutral losses 97.9769(shown in table), 0.0000

Ions Score: 36 Expect: 0.17

Matches : 6/138 fragment ions using 12 most intense peaks (help)

#	b	b^{++}	b^{0}	b^{0++}	Seq.	y	y^{++}	y*	y^{*++}	y^{0}	y^{0++}	#
1	58.0287	29.5180			G							11
2	145.0608	73.0340	127.0502	64.0287	S	1076.4127	538.7100	1059.3861	530.1967	1058.4021	529.7047	10
3	228.0979	114.5526	210.0873	105.5473	T	***989.3807***	495.1940	972.3541	486.6807	971.3701	486.1887	9
4	343.1248	172.0661	325.1143	163.0608	D	***906.3435***	453.6754	889.3170	445.1621	888.3330	444.6701	8
5	458.1518	229.5795	440.1412	220.5742	D	***791.3166***	396.1619	774.2901	387.6487	773.3060	387.1567	7
6	587.1944	294.1008	569.1838	285.0955	E	***676.2897***	338.6485	659.2631	330.1352	658.2791	329.6432	6
7	658.2315	329.6194	640.2209	320.6141	A	547.2471	274.1272	530.2205	265.6139	529.2365	265.1219	5
8	787.2741	394.1407	769.2635	385.1354	E	***476.2100***	238.6086	459.1834	230.0953	458.1994	229.6033	4
9	902.3010	451.6541	884.2904	442.6489	D	347.1674	174.0873	330.1408	165.5740	329.1568	165.0820	3
10	959.3225	480.1649	941.3119	471.1596	G	***232.1404***	116.5738	215.1139	108.0606			2
11					R	175.1190	88.0631	158.0924	79.5498			1

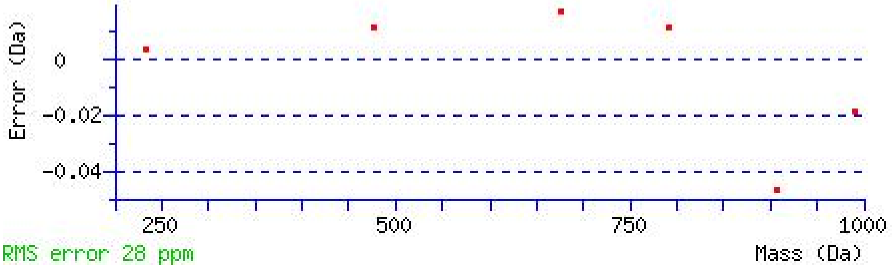

27_MS/MS Fragmentation of **ESLLSGEGKGSTDDEAEDGR**

Found in **O95180**, Voltage-dependent T-type calcium channel subunit alpha-1H OS=Homo sapiens GN=CACNA1H PE=1 SV=4

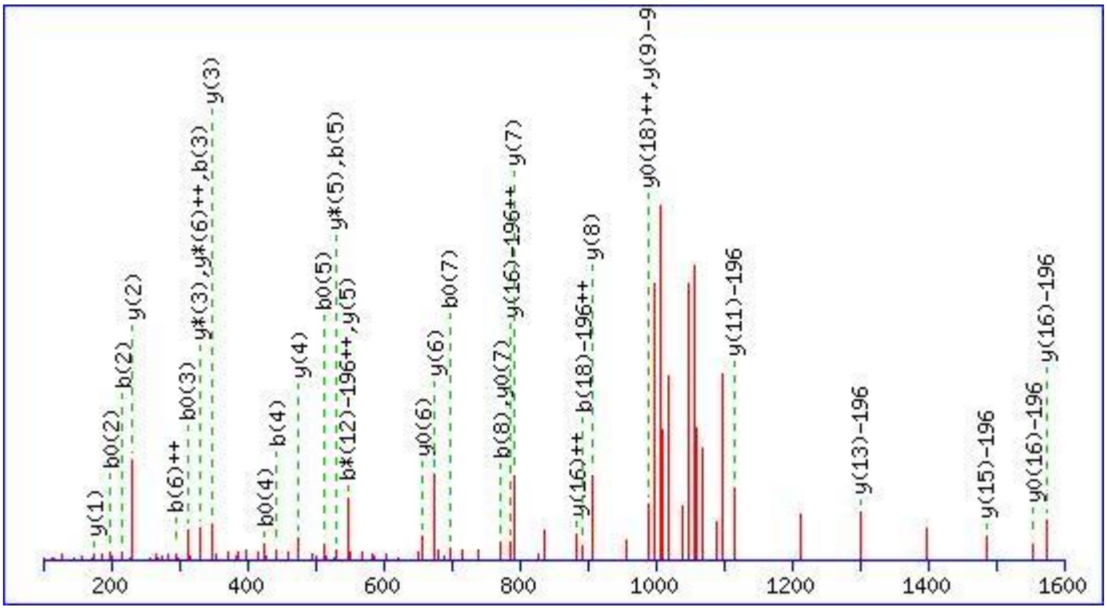

Monoisotopic mass of neutral peptide Mr(calc): 2210.8253

Variable modifications:

S11 : Phospho (ST), with neutral losses 97.9769(shown in table), 0.0000

T12 : Phospho (ST), with neutral losses 97.9769(shown in table), 0.0000

Ions Score: 113 Expect: 9e-009

Matches : 35/328 fragment ions using 37 most intense peaks (help)

#	b	b^{++}	b*	b^{*++}	b^0	b^{0++}	Seq.	y	y^{++}	y*	y^{*++}	y^0	y^{0++}	#
1	130.0499	65.5286			112.0393	56.5233	E							20
2	**217.0819**	109.0446			199.0713	100.0393	S	1886.8363	943.9218	1869.8097	935.4085	1868.8257	934.9165	19
3	**330.1660**	165.5866			312.1554	156.5813	L	1799.8042	900.4058	1782.7777	891.8925	1781.7937	891.4005	18
4	**443.2500**	222.1287			425.2395	213.1234	L	1686.7202	843.8637	1669.6936	835.3504	1668.7096	834.8584	17
5	**530.2821**	265.6447			512.2715	256.6394	S	***1573.6361***	787.3217	1556.6095	778.8084	1555.6255	778.3164	16
6	587.3035	**294.1554**			569.2930	285.1501	G	***1486.6041***	743.8057	1469.5775	735.2924	1468.5935	734.8004	15
7	716.3461	358.6767			698.3355	349.6714	E	1429.5826	715.2949	1412.5561	706.7817	1411.5720	706.2897	14
8	**773.3676**	387.1874			755.3570	378.1821	G	***1300.5400***	650.7736	1283.5135	642.2604	1282.5294	641.7684	13
9	901.4625	451.2349	884.4360	442.7216	883.4520	442.2296	K	1243.5185	622.2629	1226.4920	613.7496	1225.5080	613.2576	12

10	958.4840	479.7456	941.4575	471.2324	940.4734	470.7404	G	***1115.4236***	558.2154	1098.3970	549.7022	1097.4130	549.2101	11
11	1027.5055	514.2564	1010.4789	505.7431	1009.4949	505.2511	S	1058.4021	529.7047	1041.3756	521.1914	1040.3916	520.6994	10
12	1110.5426	555.7749	1093.5160	547.2617	1092.5320	546.7696	T	***989.3807***	495.1940	972.3541	486.6807	971.3701	486.1887	9
13	1225.5695	613.2884	1208.5430	604.7751	1207.5590	604.2831	D	***906.3435***	453.6754	889.3170	445.1621	888.3330	444.6701	8
14	1340.5965	670.8019	1323.5699	662.2886	1322.5859	661.7966	D	***791.3166***	396.1619	774.2901	387.6487	773.3060	387.1567	7
15	1469.6391	735.3232	1452.6125	726.8099	1451.6285	726.3179	E	***676.2897***	338.6485	659.2631	330.1352	658.2791	329.6432	6
16	1540.6762	770.8417	1523.6496	762.3284	1522.6656	761.8364	A	***547.2471***	274.1272	530.2205	265.6139	529.2365	265.1219	5
17	1669.7188	835.3630	1652.6922	826.8497	1651.7082	826.3577	E	***476.2100***	238.6086	459.1834	230.0953	458.1994	229.6033	4
18	1784.7457	**892.8765**	1767.7192	884.3632	1766.7351	883.8712	D	***347.1674***	174.0873	330.1408	165.5740	329.1568	165.0820	3
19	1841.7672	921.3872	1824.7406	912.8739	1823.7566	912.3819	G	***232.1404***	116.5738	215.1139	108.0606			2
20							R	***175.1190***	88.0631	158.0924	79.5498			1

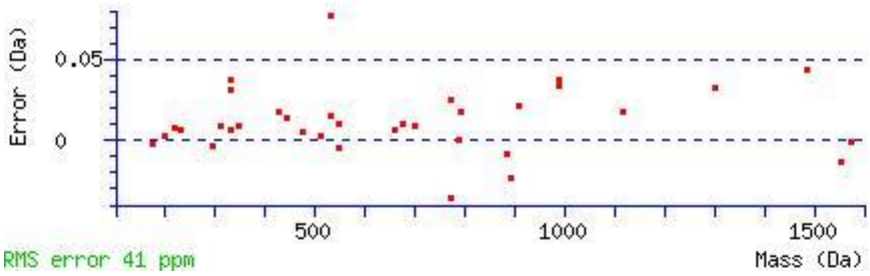

28_MS/MS Fragmentation of **RAESLDPRPLRPAALPPTK**

Found in **O95180**, Voltage-dependent T-type calcium channel subunit alpha-1H OS=Homo sapiens GN=CACNA1H PE=1 SV=4

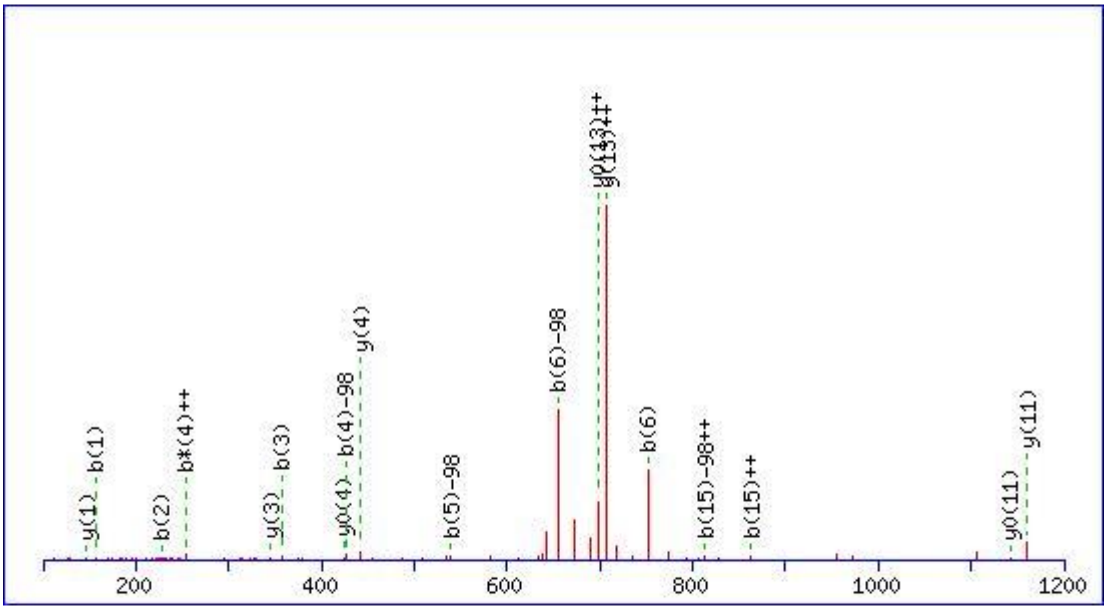

Monoisotopic mass of neutral peptide Mr(calc): 2164.1517

Variable modifications:

S4 : Phospho (ST), with neutral losses 97.9769(shown in table), 0.0000

Ions Score: 35 Expect: 0.53

Matches : 18/318 fragment ions using 29 most intense peaks (help)

#	b	b^{++}	b*	b^{*++}	b^{0}	b^{0++}	Seq.	y	y^{++}	y*	y^{*++}	y^{0}	y^{0++}	#
1	***157.1084***	79.0578	140.0818	70.5446			**R**							19
2	***228.1455***	114.5764	211.1190	106.0631			**A**	1911.0811	956.0442	1894.0545	947.5309	1893.0705	947.0389	18
3	***357.1881***	179.0977	340.1615	170.5844	339.1775	170.0924	**E**	1840.0439	920.5256	1823.0174	912.0123	1822.0334	911.5203	17
4	***426.2096***	213.6084	409.1830	205.0951	408.1990	204.6031	**S**	1711.0014	856.0043	1693.9748	847.4910	1692.9908	846.9990	16
5	***539.2936***	270.1504	522.2671	261.6372	521.2831	261.1452	**L**	1641.9799	821.4936	1624.9533	812.9803	1623.9693	812.4883	15
6	***654.3206***	327.6639	637.2940	319.1506	636.3100	318.6586	**D**	1528.8958	764.9516	1511.8693	756.4383	1510.8853	755.9463	14
7	751.3733	376.1903	734.3468	367.6770	733.3628	367.1850	**P**	1413.8689	707.4381	1396.8423	698.9248	1395.8583	698.4328	13
8	907.4744	454.2409	890.4479	445.7276	889.4639	445.2356	**R**	1316.8161	658.9117	1299.7896	650.3984	1298.8056	649.9064	12
9	1004.5272	502.7672	987.5007	494.2540	986.5166	493.7620	**P**	***1160.7150***	580.8611	1143.6885	572.3479	1142.7044	571.8559	11

**10**	1117.6113	559.3093	1100.5847	550.7960	1099.6007	550.3040	**L**	1063.6622	532.3348	1046.6357	523.8215	1045.6517	523.3295	**10**
**11**	1273.7124	637.3598	1256.6858	628.8466	1255.7018	628.3545	**R**	950.5782	475.7927	933.5516	467.2795	932.5676	466.7874	**9**
**12**	1370.7651	685.8862	1353.7386	677.3729	1352.7546	676.8809	**P**	794.4771	397.7422	777.4505	389.2289	776.4665	388.7369	**8**
**13**	1441.8023	721.4048	1424.7757	712.8915	1423.7917	712.3995	**A**	697.4243	349.2158	680.3978	340.7025	679.4137	340.2105	**7**
**14**	1512.8394	756.9233	1495.8128	748.4100	1494.8288	747.9180	**A**	626.3872	313.6972	609.3606	305.1840	608.3766	304.6920	**6**
**15**	1625.9234	**813.4654**	1608.8969	804.9521	1607.9129	804.4601	**L**	555.3501	278.1787	538.3235	269.6654	537.3395	269.1734	**5**
**16**	1722.9762	861.9917	1705.9496	853.4785	1704.9656	852.9865	**P**	***442.2660***	221.6366	425.2395	213.1234	424.2554	212.6314	**4**
**17**	1820.0290	910.5181	1803.0024	902.0048	1802.0184	901.5128	**P**	***345.2132***	173.1103	328.1867	164.5970	327.2027	164.1050	**3**
**18**	1921.0766	961.0420	1904.0501	952.5287	1903.0661	952.0367	**T**	248.1605	124.5839	231.1339	116.0706	230.1499	115.5786	**2**
**19**							**K**	***147.1128***	74.0600	130.0863	65.5468			**1**

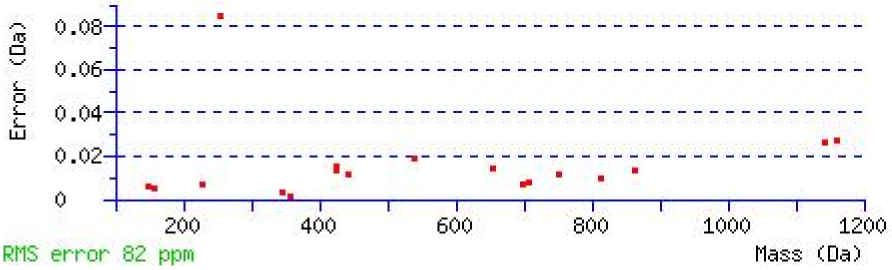

29_MS/MS Fragmentation of **RRSTFPSPEAQR**

Found in **O95180**, Voltage-dependent T-type calcium channel subunit alpha-1H OS=Homo sapiens GN=CACNA1H PE=1 SV=4

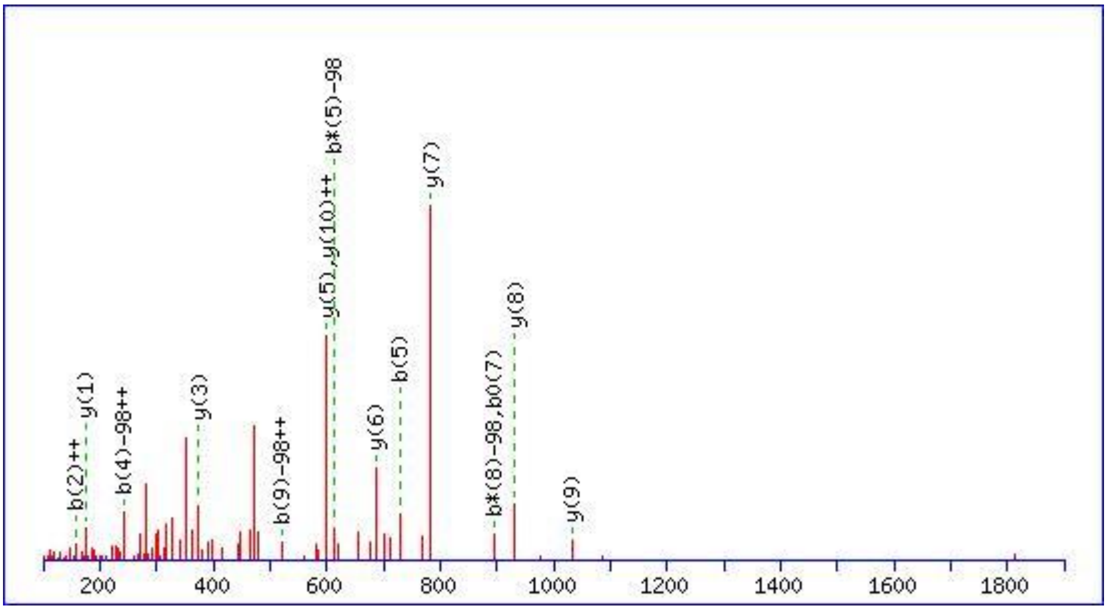

Monoisotopic mass of neutral peptide Mr(calc): 1510.7042

Variable modifications:

S3 : Phospho (ST), with neutral losses 0.0000(shown in table), 97.9769

Ions Score: 47 Expect: 0.046

Matches : 16/188 fragment ions using 19 most intense peaks (help)

#	b	b^{++}	b*	b^{*++}	b^0	b^{0++}	Seq.	y	y^{++}	y*	y^{*++}	y^0	y^{0++}	#
1	**157.1084**	79.0578	140.0818	70.5446			R							12
2	313.2095	**157.1084**	296.1829	148.5951			R	1355.6104	678.3088	1338.5838	669.7955	1337.5998	669.3035	11
3	480.2079	240.6076	463.1813	232.0943	462.1973	231.6023	S	1199.5092	600.2583	1182.4827	591.7450	1181.4987	591.2530	10
4	581.2555	291.1314	564.2290	282.6181	563.2450	282.1261	T	***1032.5109***	516.7591	1015.4843	508.2458	1014.5003	507.7538	9
5	**728.3239**	364.6656	711.2974	356.1523	710.3134	355.6603	F	***931.4632***	466.2352	914.4367	457.7220	913.4526	457.2300	8
6	825.3767	413.1920	808.3502	404.6787	807.3661	404.1867	P	***784.3948***	392.7010	767.3682	384.1878	766.3842	383.6958	7
7	912.4087	456.7080	895.3822	448.1947	894.3982	447.7027	S	***687.3420***	344.1747	670.3155	335.6614	669.3315	335.1694	6
8	1009.4615	505.2344	992.4350	496.7211	991.4509	496.2291	P	***600.3100***	300.6586	583.2835	292.1454	582.2994	291.6534	5
9	1138.5041	569.7557	1121.4776	561.2424	1120.4935	560.7504	E	503.2572	252.1323	486.2307	243.6190	485.2467	243.1270	4

**10**	1209.5412	605.2742	1192.5147	596.7610	1191.5306	596.2690	**A**	***374.2146***	187.6110	357.1881	179.0977			**3**
**11**	1337.5998	669.3035	1320.5732	660.7903	1319.5892	660.2983	**Q**	303.1775	152.0924	286.1510	143.5791			**2**
**12**							**R**	***175.1190***	88.0631	158.0924	79.5498			**1**

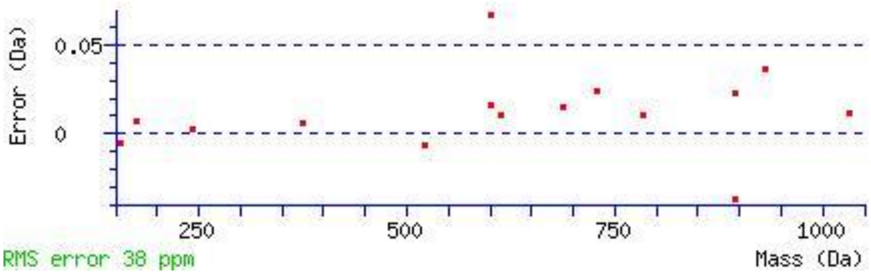

30_MS/MS Fragmentation of **RRSTFPSPEAQR**

Found in **O95180**, Voltage-dependent T-type calcium channel subunit alpha-1H OS=Homo sapiens GN=CACNA1H PE=1 SV=4

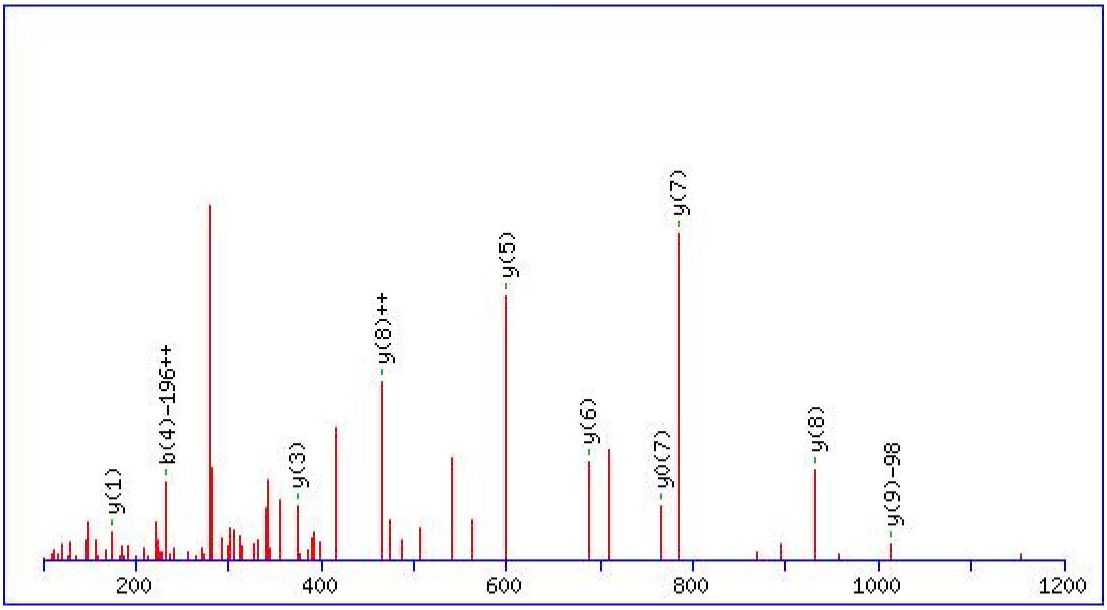

Monoisotopic mass of neutral peptide Mr(calc): 1590.6705

Variable modifications:

S3 : Phospho (ST), with neutral losses 97.9769(shown in table), 0.0000

T4 : Phospho (ST), with neutral losses 97.9769(shown in table), 0.0000

Ions Score: 32 Expect: 1.4

Matches : 10/194 fragment ions using 25 most intense peaks (help)

#	b	b^{++}	b*	b^{*++}	b^{0}	b^{0++}	Seq.	y	y^{++}	y*	y^{*++}	y^{0}	y^{0++}	#
1	157.1084	79.0578	140.0818	70.5446			R							12
2	313.2095	157.1084	296.1829	148.5951			R	1239.6229	620.3151	1222.5963	611.8018	1221.6123	611.3098	11
3	382.2310	191.6191	365.2044	183.1058	364.2204	182.6138	S	1083.5218	542.2645	1066.4952	533.7513	1065.5112	533.2592	10
4	465.2681	**233.1377**	448.2415	224.6244	447.2575	224.1324	T	***1014.5003***	507.7538	997.4738	499.2405	996.4898	498.7485	9
5	612.3365	306.6719	595.3099	298.1586	594.3259	297.6666	F	***931.4632***	466.2352	914.4367	457.7220	913.4526	457.2300	8
6	709.3893	355.1983	692.3627	346.6850	691.3787	346.1930	P	***784.3948***	392.7010	767.3682	384.1878	766.3842	383.6958	7
7	796.4213	398.7143	779.3947	390.2010	778.4107	389.7090	S	***687.3420***	344.1747	670.3155	335.6614	669.3315	335.1694	6
8	893.4740	447.2407	876.4475	438.7274	875.4635	438.2354	P	***600.3100***	300.6586	583.2835	292.1454	582.2994	291.6534	5
9	1022.5166	511.7620	1005.4901	503.2487	1004.5061	502.7567	E	503.2572	252.1323	486.2307	243.6190	485.2467	243.1270	4

**10**	1093.5538	547.2805	1076.5272	538.7672	1075.5432	538.2752	**A**	***374.2146***	187.6110	357.1881	179.0977			**3**
**11**	1221.6123	611.3098	1204.5858	602.7965	1203.6018	602.3045	**Q**	303.1775	152.0924	286.1510	143.5791			**2**
**12**							**R**	***175.1190***	88.0631	158.0924	79.5498			**1**

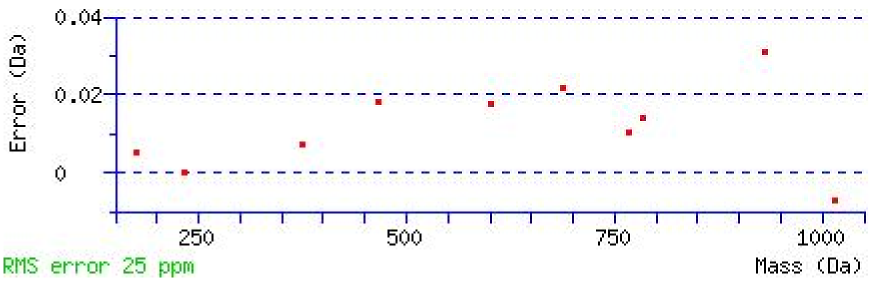

31_MS/MS Fragmentation of **STFPSPEAQR**

Found in **O95180**, Voltage-dependent T-type calcium channel subunit alpha-1H OS=Homo sapiens GN=CACNA1H PE=1 SV=4

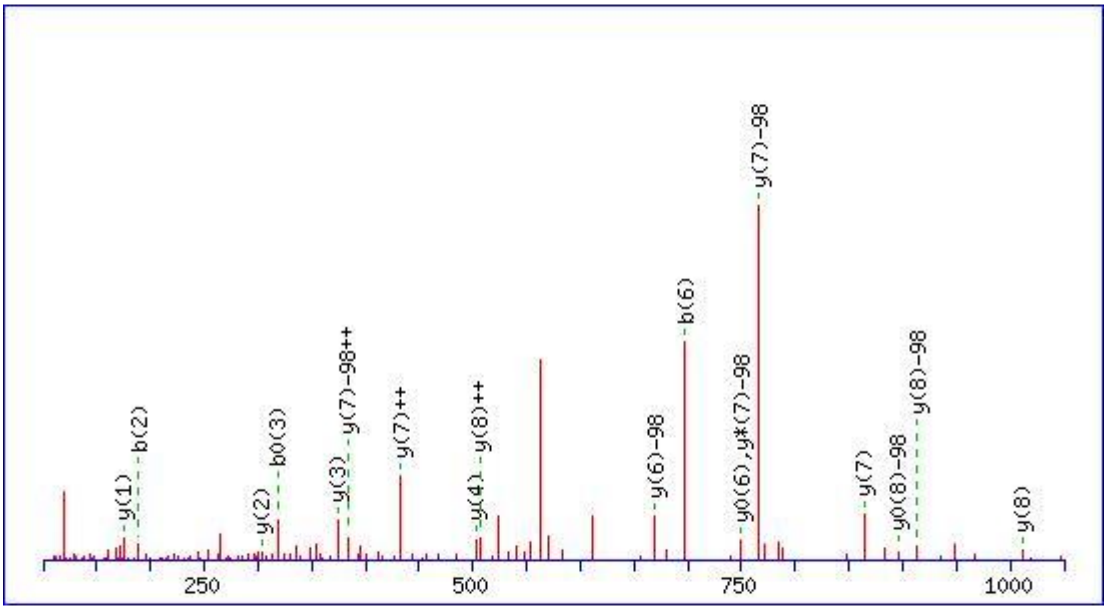

Monoisotopic mass of neutral peptide Mr(calc): 1198.5020

Variable modifications:

S5 : Phospho (ST), with neutral losses 0.0000(shown in table), 97.9769

Ions Score: 34 Expect: 0.76

Matches : 18/132 fragment ions using 30 most intense peaks (help)

#	b	b^{++}	b*	b^{*++}	b^0	b^{0++}	Seq.	y	y^{++}	y*	y^{*++}	y^0	y^{0++}	#
1	88.0393	44.5233			70.0287	35.5180	S							10
2	**189.0870**	95.0471			171.0764	86.0418	T	1112.4772	556.7422	1095.4507	548.2290	1094.4667	547.7370	9
3	336.1554	168.5813			318.1448	159.5761	F	***1011.4295***	506.2184	994.4030	497.7051	993.4190	497.2131	8
4	433.2082	217.1077			415.1976	208.1024	P	***864.3611***	432.6842	847.3346	424.1709	846.3506	423.6789	7
5	600.2065	300.6069			582.1960	291.6016	S	767.3084	384.1578	750.2818	375.6445	749.2978	375.1525	6
6	**697.2593**	349.1333			679.2487	340.1280	P	600.3100	300.6586	583.2835	292.1454	582.2994	291.6534	5
7	826.3019	413.6546			808.2913	404.6493	E	***503.2572***	252.1323	486.2307	243.6190	485.2467	243.1270	4
8	897.3390	449.1731			879.3284	440.1679	A	***374.2146***	187.6110	357.1881	179.0977			3
9	1025.3976	513.2024	1008.3710	504.6891	1007.3870	504.1971	Q	***303.1775***	152.0924	286.1510	143.5791			2

**1<br>0**							**R**	***175.1190***	88.0631	158.0924	79.5498			**1**

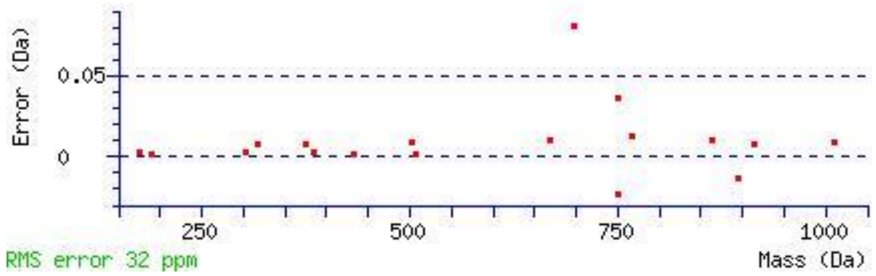

32_MS/MS Fragmentation of **RPYYADYSPTRR**

Found in **O95180**, Voltage-dependent T-type calcium channel subunit alpha-1H OS=Homo sapiens GN=CACNA1H PE=1 SV=4

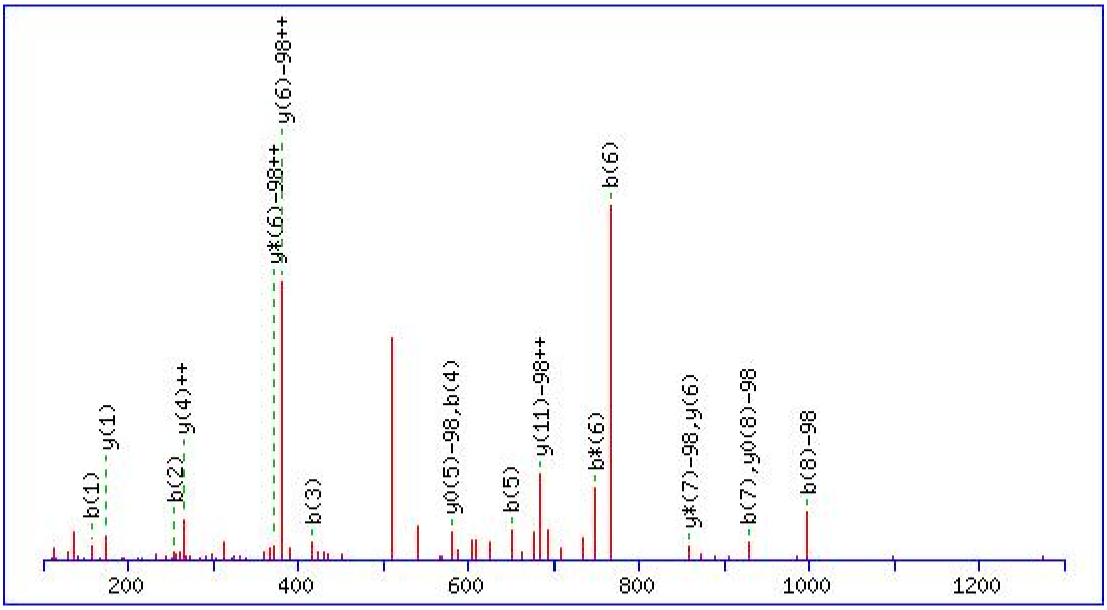

Monoisotopic mass of neutral peptide Mr(calc): 1623.7195

Variable modifications:

S8 : Phospho (ST), with neutral losses 97.9769(shown in table), 0.0000

Ions Score: 46 Expect: 0.056

Matches : 18/184 fragment ions using 29 most intense peaks (help)

#	b	b^{++}	b*	b^{*++}	b^0	b^{0++}	Seq.	y	y^{++}	y*	y^{*++}	y^0	y^{0++}	#
1	***157.1084***	79.0578	140.0818	70.5446			R							12
2	***254.1612***	127.5842	237.1346	119.0709			P	1370.6488	685.8280	1353.6222	677.3148	1352.6382	676.8227	11
3	***417.2245***	209.1159	400.1979	200.6026			Y	1273.5960	637.3016	1256.5695	628.7884	1255.5854	628.2964	10
4	***580.2878***	290.6475	563.2613	282.1343			Y	1110.5327	555.7700	1093.5061	547.2567	1092.5221	546.7647	9
5	***651.3249***	326.1661	634.2984	317.6528			A	947.4694	474.2383	930.4428	465.7250	929.4588	465.2330	8
6	***766.3519***	383.6796	749.3253	375.1663	748.3413	374.6743	D	876.4322	438.7198	859.4057	430.2065	858.4217	429.7145	7
7	***929.4152***	465.2112	912.3886	456.6980	911.4046	456.2060	Y	761.4053	381.2063	744.3787	372.6930	743.3947	372.2010	6
8	***998.4367***	499.7220	981.4101	491.2087	980.4261	490.7167	S	598.3420	299.6746	581.3154	291.1613	580.3314	290.6693	5
9	1095.4894	548.2483	1078.4629	539.7351	1077.4789	539.2431	P	529.3205	265.1639	512.2940	256.6506	511.3099	256.1586	4

**10**	1196.5371	598.7722	1179.5106	590.2589	1178.5265	589.7669	**T**	432.2677	216.6375	415.2412	208.1242	414.2572	207.6322	**3**
**11**	1352.6382	676.8227	1335.6117	668.3095	1334.6276	667.8175	**R**	331.2201	166.1137	314.1935	157.6004			**2**
**12**							**R**	**175.1190**	88.0631	158.0924	79.5498			**1**

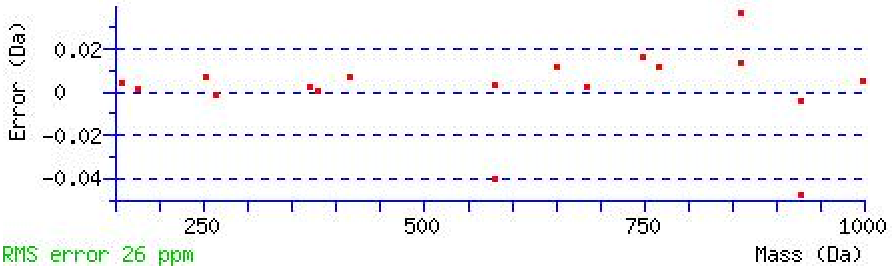

33_MS/MS Fragmentation of **VDADRPPLPQESPGAR**

Found in **O95180**, Voltage-dependent T-type calcium channel subunit alpha-1H OS=Homo sapiens GN=CACNA1H PE=1 SV=4

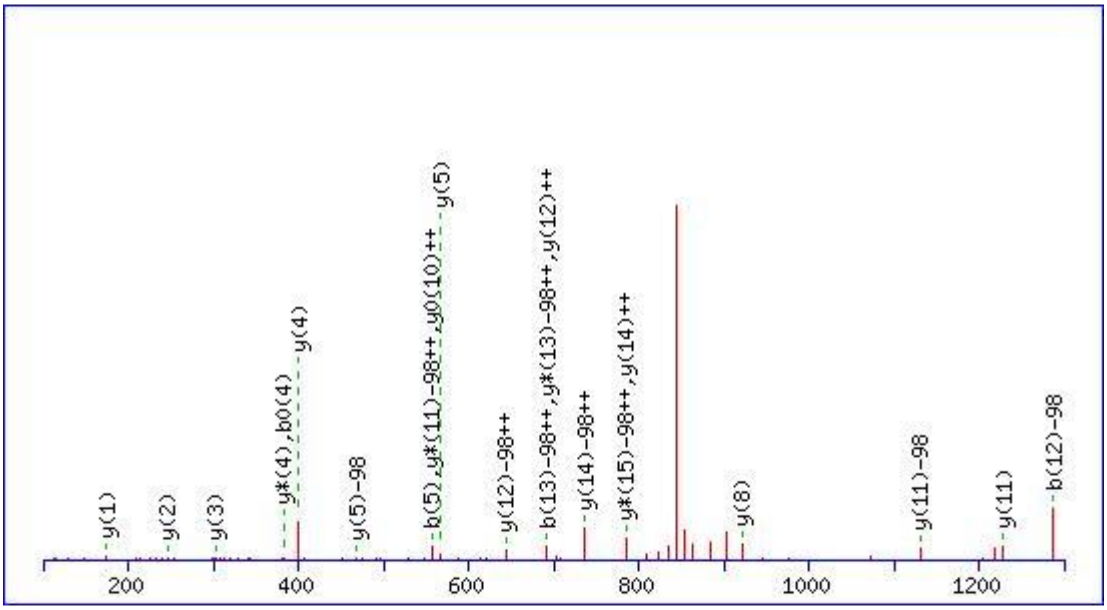

Monoisotopic mass of neutral peptide Mr(calc): 1783.8254

Variable modifications:

S12 : Phospho (ST), with neutral losses 0.0000(shown in table), 97.9769

Ions Score: 50 Expect: 0.028

Matches : 22/252 fragment ions using 23 most intense peaks (help)

#	b	b^{++}	b*	b^{*++}	b^0	b^{0++}	Seq.	y	y^{++}	y*	y^{*++}	y^0	y^{0++}	#
1	100.0757	50.5415					V							16
2	215.1026	108.0550			197.0921	99.0497	D	1685.7643	843.3858	1668.7377	834.8725	1667.7537	834.3805	15
3	286.1397	143.5735			268.1292	134.5682	A	1570.7373	785.8723	1553.7108	777.3590	1552.7268	776.8670	14
4	401.1667	201.0870			383.1561	192.0817	D	1499.7002	750.3538	1482.6737	741.8405	1481.6897	741.3485	13
5	**557.2678**	279.1375	540.2413	270.6243	539.2572	270.1323	R	1384.6733	692.8403	1367.6467	684.3270	1366.6627	683.8350	12
6	654.3206	327.6639	637.2940	319.1506	636.3100	318.6586	P	***1228.5722***	614.7897	1211.5456	606.2765	1210.5616	605.7844	11
7	751.3733	376.1903	734.3468	367.6770	733.3628	367.1850	P	1131.5194	566.2633	1114.4929	557.7501	1113.5089	557.2581	10
8	864.4574	432.7323	847.4308	424.2191	846.4468	423.7271	L	1034.4667	517.7370	1017.4401	509.2237	1016.4561	508.7317	9
9	961.5102	481.2587	944.4836	472.7454	943.4996	472.2534	P	***921.3826***	461.1949	904.3560	452.6817	903.3720	452.1896	8

10	1089.5687	545.2880	1072.5422	536.7747	1071.5582	536.2827	Q	824.3298	412.6686	807.3033	404.1553	806.3193	403.6633	7
11	1218.6113	609.8093	1201.5848	601.2960	1200.6008	600.8040	E	696.2712	348.6393	679.2447	340.1260	678.2607	339.6340	6
12	1385.6097	693.3085	1368.5831	684.7952	1367.5991	684.3032	S	***567.2287***	284.1180	550.2021	275.6047	549.2181	275.1127	5
13	1482.6625	741.8349	1465.6359	733.3216	1464.6519	732.8296	P	***400.2303***	200.6188	383.2037	192.1055			4
14	1539.6839	770.3456	1522.6574	761.8323	1521.6734	761.3403	G	***303.1775***	152.0924	286.1510	143.5791			3
15	1610.7210	805.8642	1593.6945	797.3509	1592.7105	796.8589	A	***246.1561***	123.5817	229.1295	115.0684			2
16							R	***175.1190***	88.0631	158.0924	79.5498			1

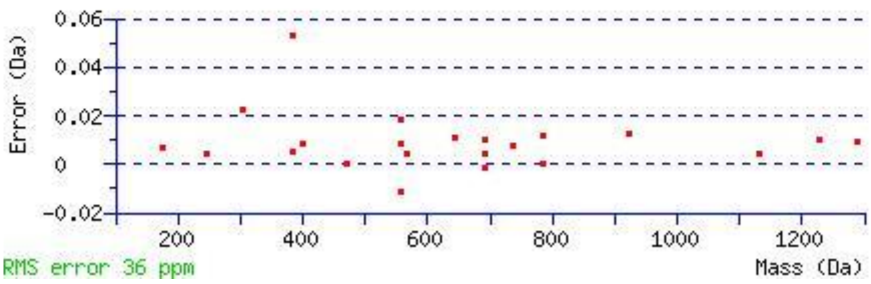

34_MS/MS Fragmentation of **SGEPLHALSPR**

Found in **O95180**, Voltage-dependent T-type calcium channel subunit alpha-1H OS=Homo sapiens GN=CACNA1H PE=1 SV=4

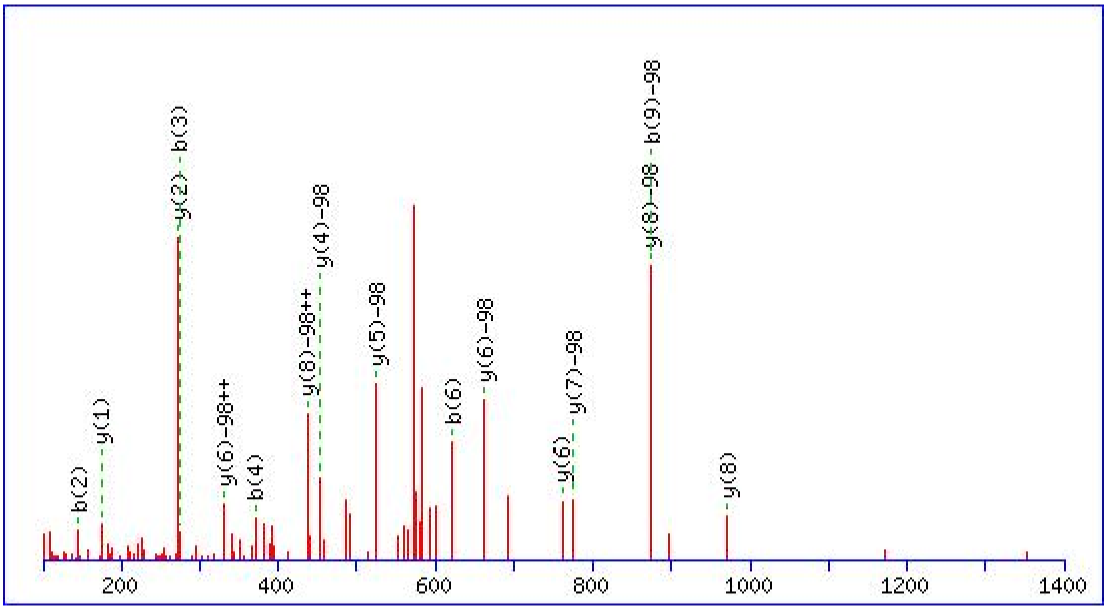

Monoisotopic mass of neutral peptide Mr(calc): 1242.5758

Variable modifications:

S9 : Phospho (ST), with neutral losses 97.9769(shown in table), 0.0000

Ions Score: 62 Expect: 0.0013

Matches : 16/152 fragment ions using 18 most intense peaks (help)

#	b	b++	b0	b0++	Seq.	y	y++	y*	y*++	y0	y0++	#
1	88.0393	44.5233	70.0287	35.5180	S							11
2	**145.0608**	73.0340	127.0502	64.0287	G	1058.5742	529.7907	1041.5476	521.2774	1040.5636	520.7854	10
3	**274.1034**	137.5553	256.0928	128.5500	E	1001.5527	501.2800	984.5261	492.7667	983.5421	492.2747	9
4	**371.1561**	186.0817	353.1456	177.0764	P	***872.5101***	436.7587	855.4835	428.2454	854.4995	427.7534	8
5	484.2402	242.6237	466.2296	233.6185	L	***775.4573***	388.2323	758.4308	379.7190	757.4468	379.2270	7
6	**621.2991**	311.1532	603.2885	302.1479	H	***662.3733***	331.6903	645.3467	323.1770	644.3627	322.6850	6
7	692.3362	346.6717	674.3257	337.6665	A	***525.3144***	263.1608	508.2878	254.6475	507.3038	254.1555	5
8	805.4203	403.2138	787.4097	394.2085	L	***454.2772***	227.6423	437.2507	219.1290	436.2667	218.6370	4
9	**874.4417**	437.7245	856.4312	428.7192	S	341.1932	171.1002	324.1666	162.5870	323.1826	162.0949	3
10	971.4945	486.2509	953.4839	477.2456	P	***272.1717***	136.5895	255.1452	128.0762			2
11					R	***175.1190***	88.0631	158.0924	79.5498			1

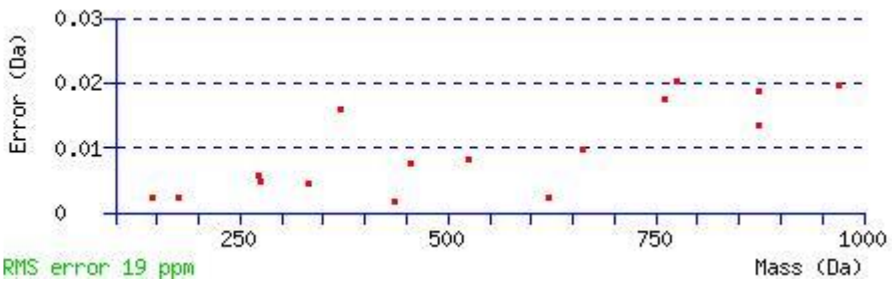

35_MS/MS Fragmentation of **IDSPRDTLDPAEPGEK**

Found in **O95180**, Voltage-dependent T-type calcium channel subunit alpha-1H OS=Homo sapiens GN=CACNA1H PE=1 SV=4

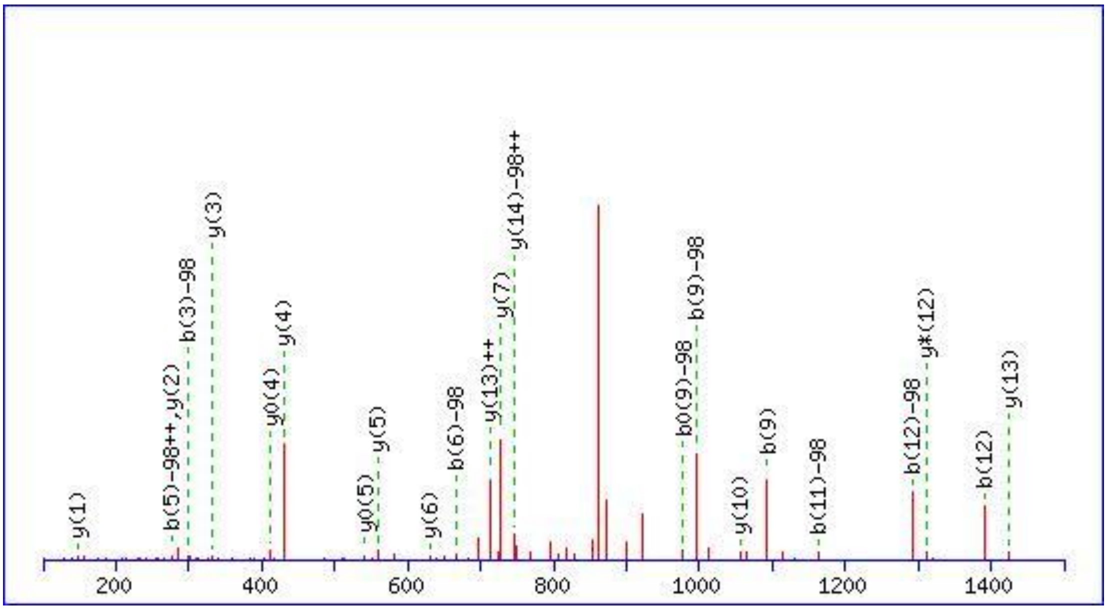

Monoisotopic mass of neutral peptide Mr(calc): 1818.8037

Variable modifications:

S3 : Phospho (ST), with neutral losses 0.0000(shown in table), 97.9769

Ions Score: 55 Expect: 0.0083

Matches : 23/254 fragment ions using 38 most intense peaks (help)

#	b	b^{++}	b*	b^{*++}	b^0	b^{0++}	Seq.	y	y^{++}	y*	y^{*++}	y^0	y^{0++}	#
1	114.0913	57.5493					I							16
2	229.1183	115.0628			211.1077	106.0575	D	1706.7269	853.8671	1689.7004	845.3538	1688.7163	844.8618	15
3	396.1166	198.5620			378.1061	189.5567	S	1591.7000	796.3536	1574.6734	787.8403	1573.6894	787.3483	14
4	493.1694	247.0883			475.1588	238.0831	P	***1424.7016***	712.8544	1407.6751	704.3412	1406.6910	703.8492	13
5	649.2705	325.1389	632.2440	316.6256	631.2600	316.1336	R	1327.6488	664.3281	1310.6223	655.8148	1309.6383	655.3228	12
6	764.2975	382.6524	747.2709	374.1391	746.2869	373.6471	D	1171.5477	586.2775	1154.5212	577.7642	1153.5372	577.2722	11
7	865.3451	433.1762	848.3186	424.6629	847.3346	424.1709	T	***1056.5208***	528.7640	1039.4942	520.2508	1038.5102	519.7587	10
8	978.4292	489.7182	961.4027	481.2050	960.4186	480.7130	L	955.4731	478.2402	938.4466	469.7269	937.4625	469.2349	9
9	**1093.4561**	547.2317	1076.4296	538.7184	1075.4456	538.2264	D	842.3890	421.6982	825.3625	413.1849	824.3785	412.6929	8

**10**	1190.5089	595.7581	1173.4824	587.2448	1172.4983	586.7528	**P**	***727.3621***	364.1847	710.3355	355.6714	709.3515	355.1794	**7**
**11**	1261.5460	631.2767	1244.5195	622.7634	1243.5355	622.2714	**A**	***630.3093***	315.6583	613.2828	307.1450	612.2988	306.6530	**6**
**12**	**1390.5886**	695.7979	1373.5621	687.2847	1372.5781	686.7927	**E**	***559.2722***	280.1397	542.2457	271.6265	541.2617	271.1345	**5**
**13**	1487.6414	744.3243	1470.6148	735.8111	1469.6308	735.3190	**P**	***430.2296***	215.6185	413.2031	207.1052	412.2191	206.6132	**4**
**14**	1544.6628	772.8351	1527.6363	764.3218	1526.6523	763.8298	**G**	***333.1769***	167.0921	316.1503	158.5788	315.1663	158.0868	**3**
**15**	1673.7054	837.3564	1656.6789	828.8431	1655.6949	828.3511	**E**	***276.1554***	138.5813	259.1288	130.0681	258.1448	129.5761	**2**
**16**							**K**	***147.1128***	74.0600	130.0863	65.5468			**1**

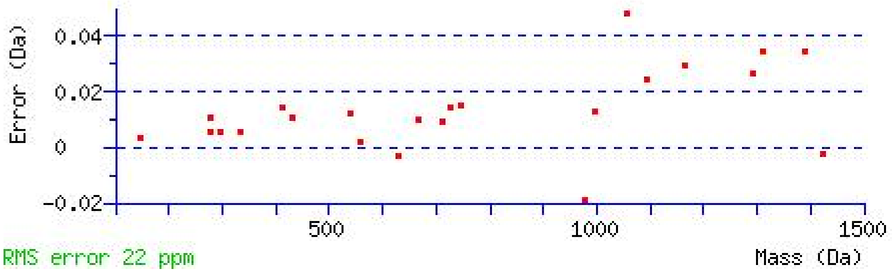

36_MS/MS Fragmentation of **TPVRPVTQGGSLQSPPR**

Found in **O95180**, Voltage-dependent T-type calcium channel subunit alpha-1H OS=Homo sapiens GN=CACNA1H PE=1 SV=4

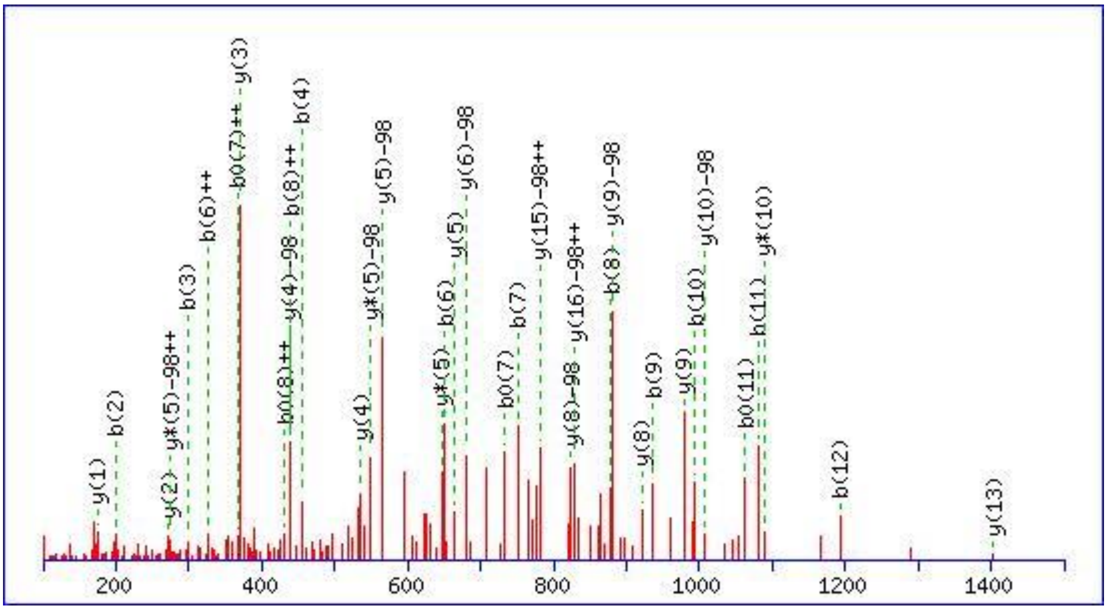

Monoisotopic mass of neutral peptide Mr(calc): 1855.9306

Variable modifications:

S14 : Phospho (ST), with neutral losses 0.0000(shown in table), 97.9769

Ions Score: 91 Expect: 2.2e-006

Matches : 36/276 fragment ions using 44 most intense peaks (help)

#	b	b^{++}	b*	b^{*++}	b^0	b^{0++}	Seq.	y	y^{++}	y*	y^{*++}	y^0	y^{0++}	#
1	102.0550	51.5311			84.0444	42.5258	T							17
2	199.1077	100.0575			181.0972	91.0522	P	1755.8902	878.4487	1738.8636	869.9354	1737.8796	869.4434	16
3	298.1761	149.5917			280.1656	140.5864	V	1658.8374	829.9223	1641.8108	821.4091	1640.8268	820.9171	15
4	454.2772	227.6423	437.2507	219.1290	436.2667	218.6370	R	1559.7690	780.3881	1542.7424	771.8749	1541.7584	771.3828	14
5	551.3300	276.1686	534.3035	267.6554	533.3194	267.1634	P	1403.6679	702.3376	1386.6413	693.8243	1385.6573	693.3323	13
6	650.3984	325.7028	633.3719	317.1896	632.3879	316.6976	V	1306.6151	653.8112	1289.5886	645.2979	1288.6045	644.8059	12
7	751.4461	376.2267	734.4196	367.7134	733.4355	367.2214	T	1207.5467	604.2770	1190.5201	595.7637	1189.5361	595.2717	11
8	879.5047	440.2560	862.4781	431.7427	861.4941	431.2507	Q	1106.4990	553.7531	1089.4725	545.2399	1088.4885	544.7479	10
9	936.5261	468.7667	919.4996	460.2534	918.5156	459.7614	G	978.4404	489.7239	961.4139	481.2106	960.4299	480.7186	9

10	993.5476	497.2774	976.5211	488.7642	975.5370	488.2722	G	921.4190	461.2131	904.3924	452.6999	903.4084	452.2078	8
11	1080.5796	540.7935	1063.5531	532.2802	1062.5691	531.7882	S	864.3975	432.7024	847.3710	424.1891	846.3869	423.6971	7
12	1193.6637	597.3355	1176.6372	588.8222	1175.6531	588.3302	L	777.3655	389.1864	760.3389	380.6731	759.3549	380.1811	6
13	1321.7223	661.3648	1304.6957	652.8515	1303.7117	652.3595	Q	664.2814	332.6443	647.2549	324.1311	646.2709	323.6391	5
14	1488.7206	744.8640	1471.6941	736.3507	1470.7101	735.8587	S	536.2228	268.6151	519.1963	260.1018	518.2123	259.6098	4
15	1585.7734	793.3903	1568.7469	784.8771	1567.7628	784.3851	P	369.2245	185.1159	352.1979	176.6026			3
16	1682.8262	841.9167	1665.7996	833.4034	1664.8156	832.9114	P	272.1717	136.5895	255.1452	128.0762			2
17							R	175.1190	88.0631	158.0924	79.5498			1

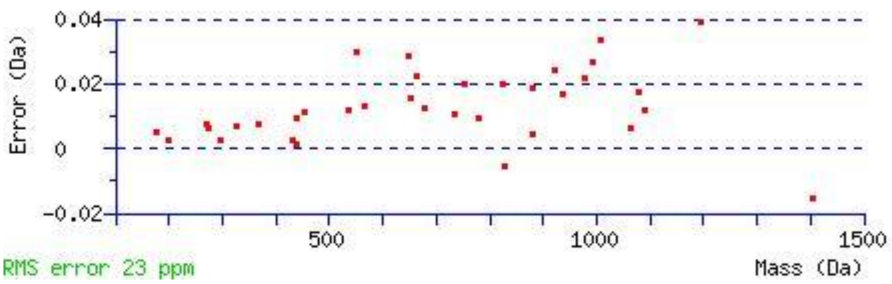

37_MS/MS Fragmentation of **LYSVDAQGFLDKPGR**

Found in **O95180**, Voltage-dependent T-type calcium channel subunit alpha-1H OS=Homo sapiens GN=CACNA1H PE=1 SV=4

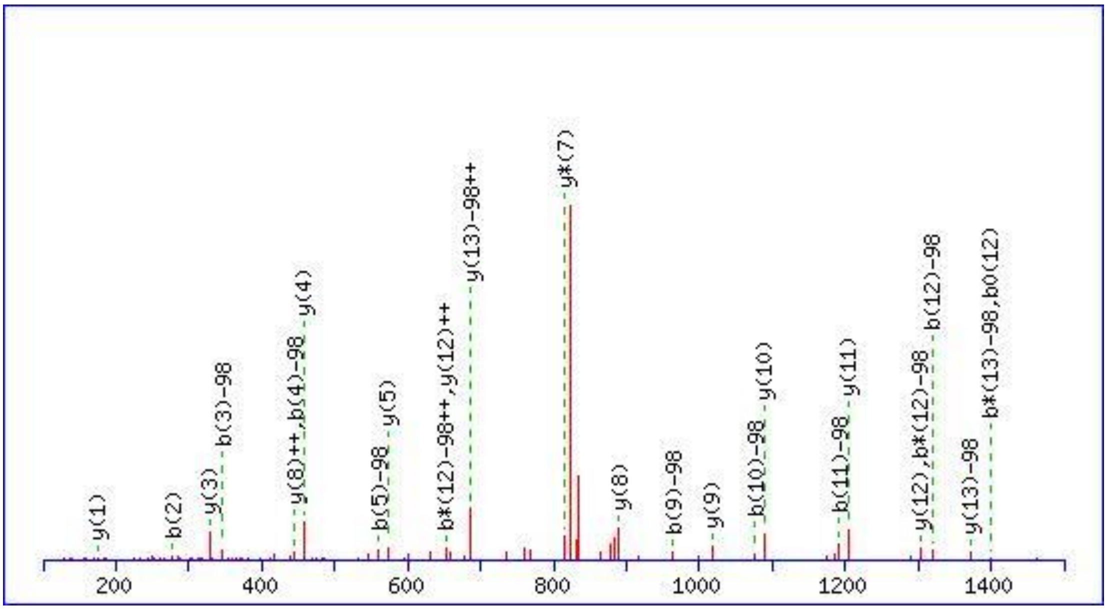

Monoisotopic mass of neutral peptide Mr(calc): 1744.8185

Variable modifications:

S3 : Phospho (ST), with neutral losses 97.9769(shown in table), 0.0000

Ions Score: 92 Expect: 1.8e-006

Matches : 26/220 fragment ions using 27 most intense peaks (help)

#	b	b^{++}	b*	b^{*++}	b^{0}	b^{0++}	Seq.	y	y^{++}	y*	y^{*++}	y^{0}	y^{0++}	#
1	114.0913	57.5493					L							15
2	**277.1547**	139.0810					Y	1534.7649	767.8861	1517.7383	759.3728	1516.7543	758.8808	14
3	**346.1761**	173.5917			328.1656	164.5864	S	**1371.7015**	**686.3544**	1354.6750	677.8411	1353.6910	677.3491	13
4	**445.2445**	223.1259			427.2340	214.1206	V	**1302.6801**	**651.8437**	1285.6535	643.3304	1284.6695	642.8384	12
5	**560.2715**	280.6394			542.2609	271.6341	D	**1203.6117**	602.3095	1186.5851	593.7962	1185.6011	593.3042	11
6	631.3086	316.1579			613.2980	307.1527	A	**1088.5847**	544.7960	1071.5582	536.2827	1070.5742	535.7907	10
7	759.3672	380.1872	742.3406	371.6740	741.3566	371.1819	Q	**1017.5476**	509.2774	1000.5211	500.7642	999.5370	500.2722	9
8	816.3886	408.6980	799.3621	400.1847	798.3781	399.6927	G	**889.4890**	**445.2482**	872.4625	436.7349	871.4785	436.2429	8
9	**963.4571**	482.2322	946.4305	473.7189	945.4465	473.2269	F	832.4676	416.7374	**815.4410**	408.2241	814.4570	407.7321	7

10	**1076.5411**	538.7742	1059.5146	530.2609	1058.5306	529.7689	**L**	685.3991	343.2032	668.3726	334.6899	667.3886	334.1979	**6**
11	**1191.5681**	596.2877	1174.5415	587.7744	1173.5575	587.2824	**D**	***572.3151***	286.6612	555.2885	278.1479	554.3045	277.6559	**5**
12	**1319.6630**	660.3352	1302.6365	651.8219	1301.6525	651.3299	**K**	***457.2881***	229.1477	440.2616	220.6344			**4**
13	1416.7158	708.8615	1399.6892	700.3483	1398.7052	699.8563	**P**	***329.1932***	165.1002	312.1666	156.5870			**3**
14	1473.7373	737.3723	1456.7107	728.8590	1455.7267	728.3670	**G**	232.1404	116.5738	215.1139	108.0606			**2**
15							**R**	***175.1190***	88.0631	158.0924	79.5498			**1**

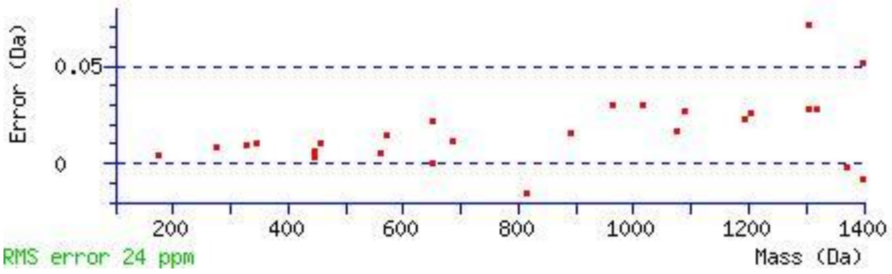

38_MS/MS Fragmentation of **KMSPPCISVEPPAEDEGSARPSAAEGGSTTLR**

Found in **O95180**, Voltage-dependent T-type calcium channel subunit alpha-1H OS=Homo sapiens GN=CACNA1H PE=1 SV=4

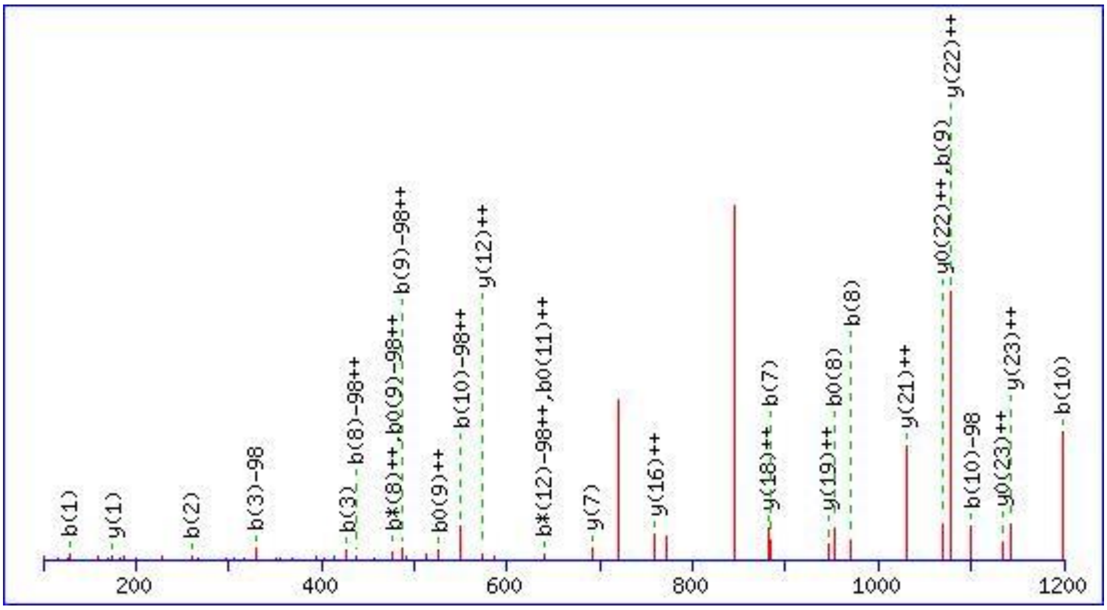

Monoisotopic mass of neutral peptide Mr(calc): 3352.4774

Variable modifications:

S3 : Phospho (ST), with neutral losses 0.0000(shown in table), 97.9769

C6 : Methylthio (C)

Ions Score: 42 Expect: 0.26

Matches : 29/550 fragment ions using 43 most intense peaks (help)

#	b	b++	b*	b*++	b0	b0++	Seq.	y	y++	y*	y*++	y0	y0++	#
1	***129.1022***	65.0548	112.0757	56.5415			K							32
2	***260.1427***	130.5750	243.1162	122.0617			M	3225.3898	1613.1985	3208.3633	1604.6853	3207.3792	1604.1933	31
3	***427.1411***	214.0742	410.1145	205.5609	409.1305	205.0689	S	3094.3493	1547.6783	3077.3228	1539.1650	3076.3388	1538.6730	30
4	524.1938	262.6006	507.1673	254.0873	506.1833	253.5953	P	2927.3510	1464.1791	2910.3244	1455.6658	2909.3404	1455.1738	29
5	621.2466	311.1269	604.2201	302.6137	603.2360	302.1217	P	2830.2982	1415.6527	2813.2717	1407.1395	2812.2876	1406.6475	28
6	770.2435	385.6254	753.2170	377.1121	752.2330	376.6201	C	2733.2454	1367.1264	2716.2189	1358.6131	2715.2349	1358.1211	27
7	***883.3276***	442.1674	866.3010	433.6542	865.3170	433.1621	I	2584.2485	1292.6279	2567.2220	1284.1146	2566.2380	1283.6226	26
8	***970.3596***	485.6834	953.3331	477.1702	952.3490	476.6782	S	2471.1645	1236.0859	2454.1379	1227.5726	2453.1539	1227.0806	25
9	***1069.4280***	535.2176	1052.4015	526.7044	1051.4175	526.2124	V	2384.1324	1192.5699	2367.1059	1184.0566	2366.1219	1183.5646	24

**10**	***1198.4706***	599.7389	1181.4441	591.2257	1180.4601	590.7337	**E**	2285.0640	1143.0356	2268.0375	1134.5224	2267.0535	1134.0304	**23**
**11**	1295.5234	648.2653	1278.4968	639.7521	1277.5128	639.2600	**P**	2156.0214	1078.5144	2138.9949	1070.0011	2138.0109	1069.5091	**22**
**12**	1392.5761	696.7917	1375.5496	688.2784	1374.5656	687.7864	**P**	2058.9687	1029.9880	2041.9421	1021.4747	2040.9581	1020.9827	**21**
**13**	1463.6133	732.3103	1446.5867	723.7970	1445.6027	723.3050	**A**	1961.9159	981.4616	1944.8894	972.9483	1943.9053	972.4563	**20**
**14**	1592.6559	796.8316	1575.6293	788.3183	1574.6453	787.8263	**E**	1890.8788	945.9430	1873.8522	937.4298	1872.8682	936.9378	**19**
**15**	1707.6828	854.3450	1690.6562	845.8318	1689.6722	845.3398	**D**	1761.8362	881.4217	1744.8096	872.9085	1743.8256	872.4165	**18**
**16**	1836.7254	918.8663	1819.6988	910.3531	1818.7148	909.8610	**E**	1646.8093	823.9083	1629.7827	815.3950	1628.7987	814.9030	**17**
**17**	1893.7469	947.3771	1876.7203	938.8638	1875.7363	938.3718	**G**	1517.7667	759.3870	1500.7401	750.8737	1499.7561	750.3817	**16**
**18**	1980.7789	990.8931	1963.7523	982.3798	1962.7683	981.8878	**S**	1460.7452	730.8762	1443.7186	722.3630	1442.7346	721.8710	**15**
**19**	2051.8160	1026.4116	2034.7894	1017.8984	2033.8054	1017.4064	**A**	1373.7132	687.3602	1356.6866	678.8469	1355.7026	678.3549	**14**
**20**	2207.9171	1104.4622	2190.8906	1095.9489	2189.9065	1095.4569	**R**	1302.6761	651.8417	1285.6495	643.3284	1284.6655	642.8364	**13**
**21**	2304.9699	1152.9886	2287.9433	1144.4753	2286.9593	1143.9833	**P**	1146.5749	573.7911	1129.5484	565.2778	1128.5644	564.7858	**12**
**22**	2392.0019	1196.5046	2374.9753	1187.9913	2373.9913	1187.4993	**S**	1049.5222	525.2647	1032.4956	516.7515	1031.5116	516.2594	**11**
**23**	2463.0390	1232.0231	2446.0125	1223.5099	2445.0284	1223.0179	**A**	962.4902	481.7487	945.4636	473.2354	944.4796	472.7434	**10**
**24**	2534.0761	1267.5417	2517.0496	1259.0284	2516.0656	1258.5364	**A**	891.4530	446.2302	874.4265	437.7169	873.4425	437.2249	**9**
**25**	2663.1187	1332.0630	2646.0922	1323.5497	2645.1082	1323.0577	**E**	820.4159	410.7116	803.3894	402.1983	802.4054	401.7063	**8**
**26**	2720.1402	1360.5737	2703.1136	1352.0605	2702.1296	1351.5684	**G**	**691.3733**	346.1903	674.3468	337.6770	673.3628	337.1850	**7**
**27**	2777.1616	1389.0845	2760.1351	1380.5712	2759.1511	1380.0792	**G**	634.3519	317.6796	617.3253	309.1663	616.3413	308.6743	**6**
**28**	2864.1937	1432.6005	2847.1671	1424.0872	2846.1831	1423.5952	**S**	577.3304	289.1688	560.3039	280.6556	559.3198	280.1636	**5**

**29**	2965.2414	1483.1243	2948.2148	1474.6110	2947.2308	1474.1190	**T**	490.2984	245.6528	473.2718	237.1396	472.2878	236.6475	**4**
**30**	3066.2890	1533.6482	3049.2625	1525.1349	3048.2785	1524.6429	**T**	389.2507	195.1290	372.2241	186.6157	371.2401	186.1237	**3**
**31**	3179.3731	1590.1902	3162.3465	1581.6769	3161.3625	1581.1849	**L**	288.2030	144.6051	271.1765	136.0919			**2**
**32**							**R**	**175.1190**	88.0631	158.0924	79.5498			**1**

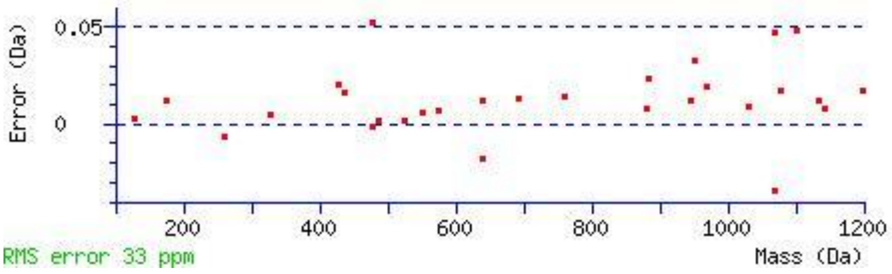

39_MS/MS Fragmentation of **RTPSCEATPHR**

Found in **O95180**, Voltage-dependent T-type calcium channel subunit alpha-1H OS=Homo sapiens GN=CACNA1H PE=1 SV=4

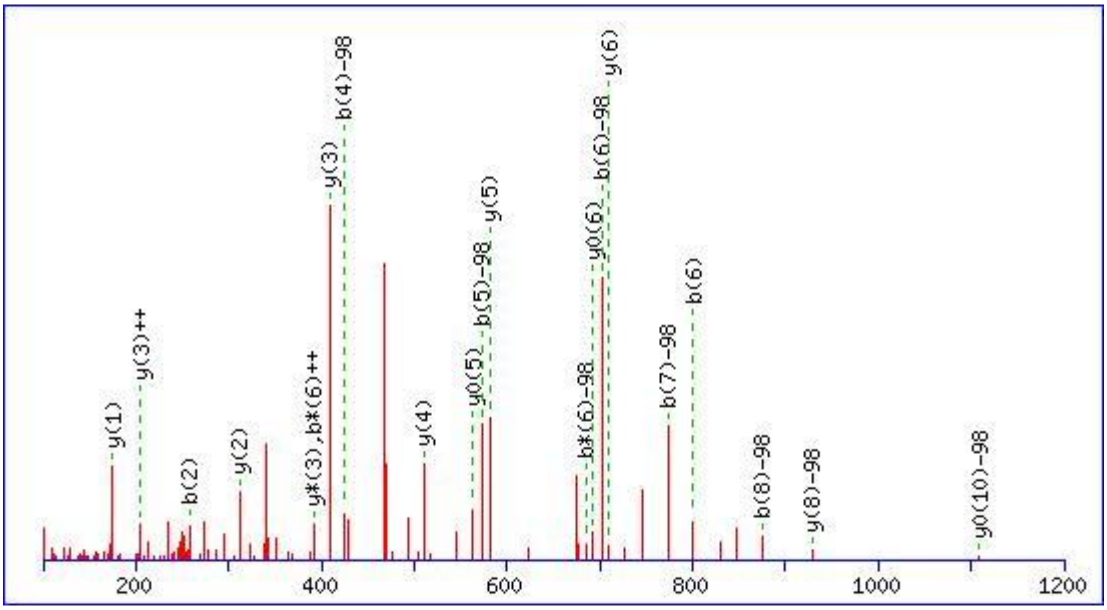

Monoisotopic mass of neutral peptide Mr(calc): 1379.5475

Variable modifications:

S4 : Phospho (ST), with neutral losses 97.9769(shown in table), 0.0000

C5 : Methylthio (C)

Ions Score: 46 Expect: 0.036

Matches : 22/172 fragment ions using 34 most intense peaks (help)

#	b	b^{++}	b*	b^{*++}	b^{0}	b^{0++}	Seq.	y	y^{++}	y*	y^{*++}	y^{0}	y^{0++}	#
1	157.1084	79.0578	140.0818	70.5446			R							11
2	**258.1561**	129.5817	241.1295	121.0684	240.1455	120.5764	T	1126.4768	563.7421	1109.4503	555.2288	1108.4663	554.7368	10
3	355.2088	178.1081	338.1823	169.5948	337.1983	169.1028	P	1025.4291	513.2182	1008.4026	504.7049	1007.4186	504.2129	9
4	**424.2303**	212.6188	407.2037	204.1055	406.2197	203.6135	S	**928.3764**	464.6918	911.3498	456.1786	910.3658	455.6865	8
5	**573.2272**	287.1172	556.2006	278.6040	555.2166	278.1120	C	859.3549	430.1811	842.3284	421.6678	841.3444	421.1758	7
6	**702.2698**	351.6385	685.2432	343.1253	684.2592	342.6333	E	**710.3580**	355.6826	693.3315	347.1694	692.3474	346.6774	6
7	**773.3069**	387.1571	756.2804	378.6438	755.2963	378.1518	A	**581.3154**	291.1613	564.2889	282.6481	563.3049	282.1561	5
8	**874.3546**	437.6809	857.3280	429.1677	856.3440	428.6756	T	**510.2783**	255.6428	493.2518	247.1295	492.2677	246.6375	4
9	971.4073	486.2073	954.3808	477.6940	953.3968	477.2020	P	**409.2306**	205.1190	392.2041	196.6057			3

**10**	***1108.4663***	554.7368	1091.4397	546.2235	1090.4557	545.7315	**H**	***312.1779***	156.5926	295.1513	148.0793			**2**
**11**							**R**	***175.1190***	88.0631	158.0924	79.5498			**1**

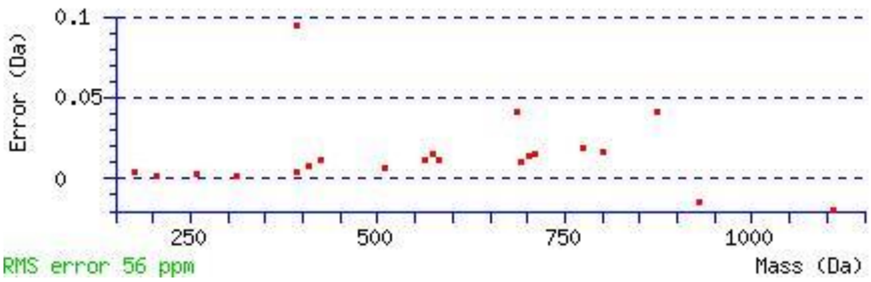

Supplement: Supplementary file 1 [file biomedicines-11-02891-s001.zip › biomedicines-2582185-supplementary.pdf]
